# Tunable Supramolecular Ag^+^-Host Interactions in Pillar[*n*]arene[*m*]quinones and Ensuing Specific Binding to 1-Alkynes

**DOI:** 10.3390/molecules28207009

**Published:** 2023-10-10

**Authors:** Yumei Zhu, Jorge Escorihuela, Haiying Wang, Andrew C.-H. Sue, Han Zuilhof

**Affiliations:** 1 School of Pharmaceutical Science & Technology, Tianjin University, 92 Weijin Road, Nankai District, Tianjin 300072, China; 2 Departamento de Química Orgánica, Universitat de València, Avda. Vicente Andrés Estellés s/n, 46100 València, Spain; 3 College of Chemistry and Chemical Engineering, Xiamen University, 422 Siming South Road, Siming District, Xiamen 361005, China; 4 Laboratory of Organic Chemistry, Wageningen University, Stippeneng 4, 6708 WE Wageningen, The Netherlands

**Keywords:** pillararenes, host–guest interactions, metal ion inclusion, tunable cavity, supramolecular chemistry

## Abstract

We developed an improved, robust synthesis of a series of pillar[*6*]arenes with a varying number (0–3) of quinone moieties in the ring. This easy-to-control variation yielded a gradually less electron-rich cavity in going from zero to three quinone units, as shown from the strength of host–guest interactions with silver ions. Such macrocycle-Ag_2_ complexes themselves were shown to display an unprecedented, sharp distinction between terminal alkynes, which strongly bound to such complexes, and internal alkynes, internal alkenes and terminal alkenes, which do hardly bind.

## 1. Introduction

Tunability in host–guest chemistry is typically achieved with one or a few hosts and a series of gradually varied guest molecules [1]. This is a highly useful approach especially if the synthesis of the host molecules is relatively complex, if the aim is to achieve maximum association constants or if the specific guest is not so important and simply association constants in a certain range are required. Examples of these abound; in many materials science applications, host–guest interactions are maximized to give maximum overall strength to still-dynamic covalent materials, while tunability is an asset if the balance between binding and exchange kinetics demands attention to both. Sometimes, in contrast, it is a fixed class of guests that is focused on, and the hosts are tuned to a particular interaction. Along those latter lines, we study a series of pillararene host molecules used to study tunability toward Ag^+^ and 1-alkynes in host–guest chemistry.

Pillar[*n*]arenes are macrocyclic hosts with a cylindrical, pillar-like rigid structure that have been investigated extensively with respect to their host–guest chemistry [1,2]. Pillar[*n*]arenes are composed of *n* hydroquinone monomers covalently connected with methylene bridges at the 2,5-*para* positions; at present, pillar[*5*]arenes (P5s) and pillar[*6*]arenes (P6s) have attracted most attention, due to their relative ease of formation [3]. Pillar[*5*]arenes display a 5-fold symmetry and a cavity diameter of about 5 Å, i.e., similar to that of α-cyclodextrin [4]. Analogously, pillar[*6*]arenes have a hexagon-like structure with a cavity size of, ca. 6.7 Å [5]. The cavities of pillar[*n*]arenes are electron-rich due to the electron-donating hydroxyl or alkoxy groups on both rims. This particular feature has driven most of the aforementioned rich host–guest chemistry, and is, for example, used in a wide variety of separation and extraction applications [1,6,7,8]. In the last 5 years, our group has worked on the systematic development of synthetic methods that can change the characteristics of P5s and P6s, including the synthesis of rim-differentiated pillararenes and tiararenes [9,10,11] (in which one of the rims only has -H as substituents, rather than, e.g., alkoxy groups), and have studied a wide range of supramolecular complexation behaviors of these materials [12,13,14]. In our studies, we had thus prepared both electron-rich and ‘neutral’ cavities, but did not delve into electron-poor cavities.

Fortunately, other groups did. The partial oxidation of P5s to provide one quinone ring (pillar[*5*]arene[*1*]quinones, or P5Q1) [15] allowed the study of the effects of a slightly more electron-withdrawing cavity on the association constants to guest molecules such as terminal dibromo- or dicyanoalkanes [16]. These findings were underscored by a series of DFT calculations of pillar[*n*]quinones (*n* = 3–7) [17]. More recently, for P6s, the gradual oxidation [18], even up to the full, sixfold hydroquinone [19], has been reported, but no binding trend studies for P6Q*ns* have been reported by comparing their host–guest interactions with guests.

We are also interested in Ag(I) complexation, partially in view of our use of Ag^+^ in binding to specific natural alkenes [20,21]. In 2019, Huang and co-workers reported the single-crystal structure of a peralkylated P5 with the silver salt CF_3_COOAg, revealing that this silver salt formed a unique binuclear structure that inserted directly into the cavity [22]. We hypothesized that P6s could complex with silver ions at least as strongly, due to an increased rotational freedom and the larger Ag∙∙∙Ag distances that could be combined with cavity-centered binding. Based on this, we thus reasoned that P6Q*n* compounds could function as a tunable platform to bind Ag^+^ and still display an open Ag∙∙∙alkene/alkyne binding site, the electronics of which can hopefully be tuned via the number *n* of quinone groups.

**Figure 1:**
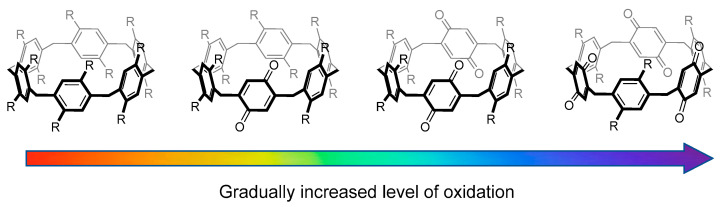
Gradual oxidation of a series of pillar[*6*]arenes to contain 0–3 quinone moieties allows for fine-tuned host–guest interactions between silver ions and pillararene hosts.

We thus report here the complexation of Ag^+^ by a series of P6Q*n* compounds (Figure 1), obtained via slightly improved robust synthetic methods, using a combination of nuclear magnetic resonance (NMR), mass spectrometry (MS) and density functional theory (DFT) studies, and present preliminary experimental and computational studies of the complexation of the resulting [P6Q*n*∙∙∙Ag]^+^ hosts to alkynes and alkenes.

## 2. Results and Discussion

### 2.1. Synthesis of Different Pillararene Hosts

**Figure 2:**
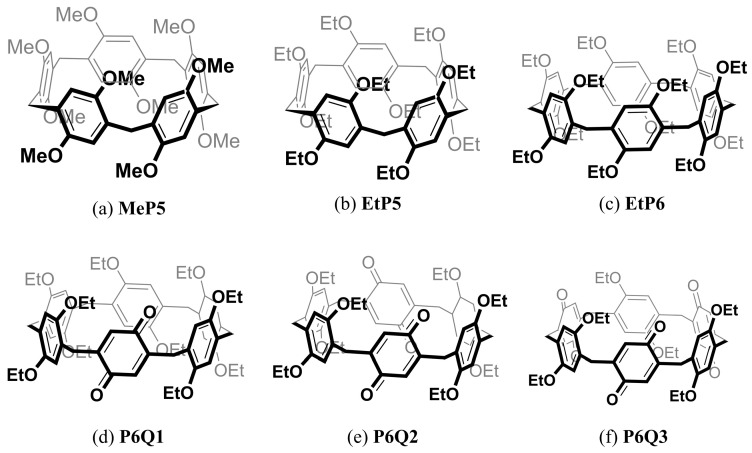
Structure of compounds under current study.

For the first part (study of [P6Q*n*∙∙∙Ag]^+^ complexation), we needed at least 2 g of each of the P6 derivatives under study (Figure 2). Compound methoxy-pillar[*5*]arene (**MeP5**) was synthesized on a multigram scale via a literature method (see Supporting Information) [23]. Such an efficient route was also desired for ethoxy-pillar[*6*]arene (**EtP6**), as needed for the subsequent synthesis and follow-up studies of pillar[*6*]arene[*1*]quinone, pillar[*6*]arene[*2*]quinone and pillar[*6*]arene[*3*]quinone (**P6Q1**, **P6Q2** and **P6Q3**, respectively). For **EtP6,** a variety of synthetic procedures have also been reported, e.g., using a catalyst template [11,24,25] or solvent templates such as chloroform [26,27] and chlorocyclohexane [28] or an acid as a catalyst [29,30]. For example, with chlorocyclohexane as a solvent, the reported yields of P6s reached as high as 87%. However, this was only for 1,4-bis(methyl-cyclohexyl ether) benzene used as the monomer in the pillararene synthesis, which is geometrically somewhat similar to the solvent. Repetition of this procedure using 1,4-ethoxybenzene as the monomer of interest and chloroform as the solvent provided only unstable yields and required large amounts of anhydrous chloroform, thus prohibiting the synthesis on a multi-gram scale. After investigation of a variety of methods, we optimized the synthesis of **EtP6** using [ChCl][FeCl_3_]_2_ as the catalyst template [24]. As the eventually reached optimized procedure, a 2:1 mixture of ferric chloride and choline chloride was ground and heated to 120 °C with stirring to yield a dark liquid. To this, a solution of 1,4-diethoxybenzene and paraformaldehyde dissolved in dichloromethane was added and stirred for a week at room temperature. This procedure increased the yield of **EtP6** to a robust 35% (6.20 g; 5.83 mmol), produced **EtP5** as minor side-product (yield: 7%), and thus provided a reliable method to synthetize this P6 derivative (Scheme S2). The ratio **EtP6**/**EtP5** was consistently high during this synthesis, but the formation of **EtP6** continued to increase up to at least five days, so seven days was chosen as the standard reaction time.

From **EtP6**, the different oxidation products **P6Q1**, **P6Q2** and **P6Q3** were synthesized with another modification from the literature [18], which uses overnight reactions after addition of the required number of equivalents of Ce(IV) ammonium nitrate (NH_4_)_2_[Ce(NO_3_)_6_] in DCM/THF 1:1 solvent mixtures (Scheme S3). This procedure gave consistently low yields (ca. 10–20%, despite literature values of 62–72% at smaller scales). However, changing the solvent composition to increased DCM fractions markedly increased the isolated yield of **P6Q1**, **P6Q2** and **P6Q3** (Table 1) to 30–50% (typically 350–500 mg of desired product per reaction, with smaller amounts of other oxidized products). Here, the DCM/THF ratios were optimized to accommodate the increasing amounts of Ce(IV) salts, which are poorly soluble in DCM.

### 2.2. Determining Association Constants of Four Hosts with CF_3_COOAg via NMR Titration Experiments

Next, we investigated the complexation of all six compounds (a)–(f) (see Figure 2) with CF_3_COOAg via NMR titration experiments. In line with single-crystal structures from Huang’s research [22], and based on confirmation via Job Plot analysis, both **MeP5** and **EtP5** form complexes with two Ag ions. We then analyzed the analogous **EtP6** and P6Q*ns* accordingly. For **EtP6** and **P6Q1,** maxima in the Job Plot were observed at the same host/Ag^+^ ratios as for the P5s, strongly suggesting that both **EtP6** and **P6Q1** form 1:2 complexes with silver trifluoroacetate (Figures S8–S11). In contrast, the ^1^H NMR spectra of **P6Q2** and **P6Q3** did not display any obvious chemical shifts (∆δ < 0.001 ppm) after the addition of CF_3_COOAg (Figures S12 and S13). Therefore, we inferred that **P6Q2** and **P6Q3** do not yield significant complex formation with CF_3_COOAg in CDCl_3_/THF-d_8_ (1:2, *v*/*v*).

The observed downfield shifts in the ^1^H NMR titration experiments of **MeP5**, **EtP5**, **EtP6** and **P6Q1** allowed us to calculate the binding strengths. Different protons show different chemical shifts, as the guest molecules in the cavity have different effects on them. Protons on the **EtP6** benzene ring displayed the largest chemical shift (∆δ up to 0.005 ppm), while the largest chemical shift for **P6Q1** was, ca. 0.002 ppm. We then used the observed ∆δ values of the peaks for the protons in the benzene ring to calculate the binding constants for **EtP6** and **P6Q1** (Figures S18–S21). Since no complexes with one Ag^+^ were observed and binding of the second Ag^+^ seems to be catalyzed by complexation of the first Ag^+^ (or, phrased differently, CF_3_COOAg inside P5s or P6s strongly prefers dimer formation; see below), we calculated the binding constant by taking the combination of two CF_3_COOAg as one guest for the calculation, so the data were set into a 1:1 complex calculation system, in which the guest concentration taken into account in the binding constant calculations was half of that of the experimental CF_3_COOAg concentration [31,32].

From these data, binding constants can be obtained, which allow for a detailed comparison of the host–guest interactions. First, the difference in association constants for **MeP5** and **EtP5** with CF_3_COOAg is not negligible. The ethoxy groups at the rims of **EtP5** influence the cavity in a number of ways differently from analogous methoxy groups; they shelter the cavity more from the solvent, allowing greater interaction of the Ag^+^ with the rings; the additional ethylene moiety will likely also strengthen the interaction with the trifluoroacetate anion. Furthermore, the binding constants of **EtP5** and **EtP6** with CF_3_COOAg are nearly identical (Table 2). Apparently, the complex balance between freedom to bind (favoring complexation of P6), loss of rotational freedom (disfavoring for P6), optimal Ag∙∙∙ring distances, Ag^+^∙∙∙Ag^+^ interactions and solvent release from the cavity turns out to yield a near-unity ratio, perhaps slightly favoring **EtP5**. For **P6Q1**, the Ag∙∙∙host association is significantly weaker, and for **P6Q2** and **P6Q3,** no association complex could even be calculated in this manner; the hypothesized lower electron density indeed seems to reveal its presence here.

### 2.3. Complexation of the Host Molecule with Silver Trifluoroacetate by ESI-MS

From the NMR titration experiments, we obtained a binding trend with two silver trifluoroacetate: **EtP5** ≈ **EtP6** > **MeP5** > **P6Q1**. To confirm this and to probe for the presence of any weakly bound Ag∙∙∙host complexes with **P6Q2** and **P6Q3** as well, we conducted electrospray ionization mass spectrometry (ESI-MS) experiments, in which we studied the complexation of the host molecule with silver trifluoroacetate (1:1 ratio; 1.0 mmol/L of host). Using the nominal level (NL) value as a semi-quantitative intensity indicator, the [host + CF_3_COOAg + Ag]^+^ peak gives the same trend in binding strength as that of the NMR titration experiment, but due to the sensitivity of the ESI-MS experiment also, data for the **P6Q2** and **P6Q3** hosts could be obtained, **EtP5** ≈ **EtP6** > **MeP5** > **P6Q1** > **P6Q2** > **P6Q3**, using NL values of 1.58, 1.55, 1.43, 1.05, 0.86 and 0.15 × 10^9^, respectively (Figure S28).

**Figure 3:**
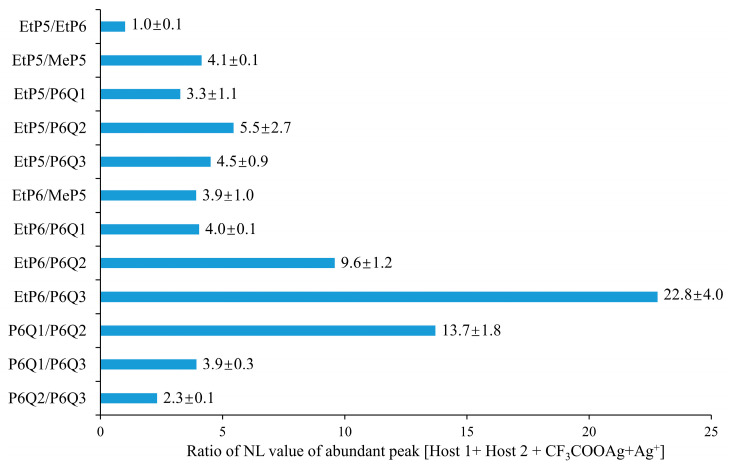
Histogram of competitive complexation of two different pillararenes and 2 equiv. CF_3_COOAg.

Since both the NMR-based K values and the ESI-MS-based NL values indicate Ag^+^ binding for these compounds, we obtained further information about the relative binding strengths of the various P5 and P6 derivatives from competition experiments. In such an experiment, we mixed two different hosts and two equivalents of guest (1.0 mmol/L of each host, 2.0 mmol/L of CF_3_COOAg) and compared the ratios of the NL values of different [host + CF_3_COOAg + Ag]^+^ peaks in ESI-MS experiments. The resulting ratios are shown in Figure 3. These relative values should not be taken as equilibrium constants, as, e.g., substrate-specific differences in the ESI process also play a role in determining the intensity of the peaks, but taken in combination with the above, they confirm the following trend: **EtP5** ≈ **EtP6** > **MeP5** > **P6Q1** > **P6Q2** > **P6Q3**. The occurrence of this trend thus strongly supports the hypothesis that the binding strength toward the silver ions can be precisely tuned via the number of quinone moieties in the P6Q*n* host.

### 2.4. DFT-Optimized Structures for Six Different Pillararene-Ag_2_ Complexes

**Figure 4:**
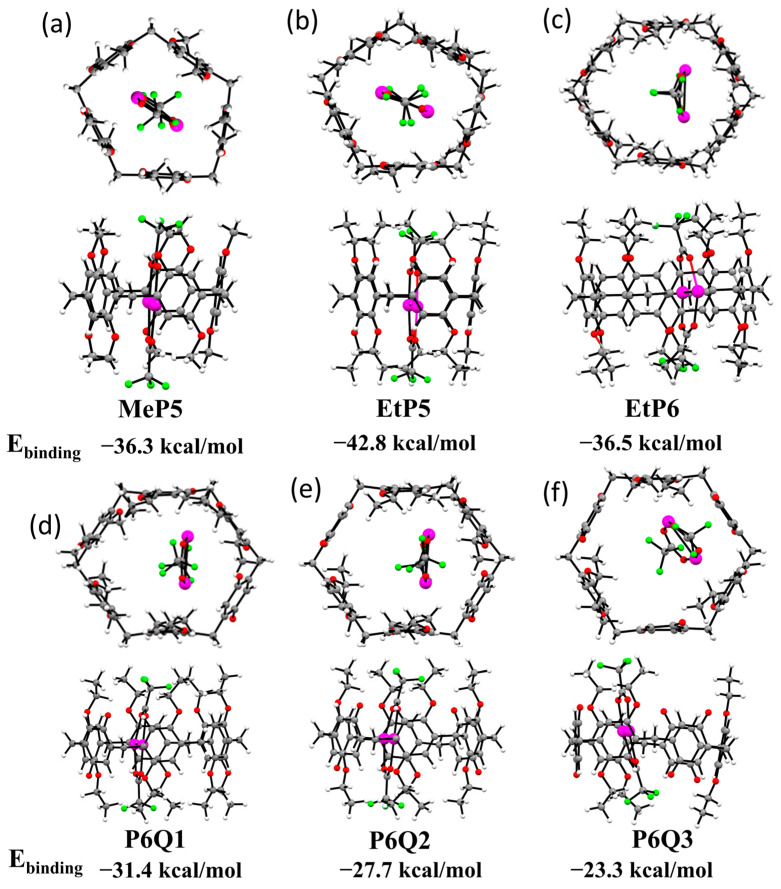
Top and side views of optimized structures for (**a**) **MeP5**, (**b**) **EtP5**, (**c**) **EtP6**, (**d**) **P6Q1**, (**e**) **P6Q2** and (**f**) **P6Q3** with two CF_3_COOAg in the cavity and their computational calculated binding energies. Color legend: grey = C, white = H, red = O, green = F, purple = Ag.

To further understand the differences between the various host–guest complexes, DFT calculations were performed at the wB97XD/Def2TZV level of theory [33]. These calculations led to the structures (top view and side view) as depicted in Figure 4. It should be noted that the binding of two CF_3_COOAg entities is strongly preferred over binding only one; while the binding of one CF_3_COOAg follows roughly the same trend over the various hosts as two CF_3_COOAg units, the values are much smaller: from +14 to +21 kcal/mol for one CF_3_COOAg, to −23 to −43 kcal/mol for two CF_3_COOAg. These data confirm the earlier observation that only complexes with two CF_3_COOAg units are observed, both for P5 by Huang et al. [16] and for the entire range of P5 and P6 hosts by us. In all six host–guest complexes, the two Ag ions form a planar complex with two trifluoromethyl carboxylate groups, in which each of the Ag ions forms a linear O∙∙∙Ag∙∙∙O interaction to couple to the carboxylate O atoms. The resulting planar eight-membered ring is oriented parallel to the aromatic rings of the P5 hosts. As can be seen from the top views, for the P5 structures, the Ag ions are positioned slightly out of the center of the cavity, in line with, e.g., the crystal structures reported by Huang and co-workers, but the ring itself maintains its five-fold symmetry. For the P6 structures, the positioning of the Ag dimer away from the center of the ring is more pronounced. In the **EtP6**, **P6Q1** and **P6Q2** structures, each of the Ag ions interacts with two oppositely placed aromatic rings, and these interactions really distort the overall shape of the ring, pulling two of its sides closer together. This is not the case anymore for the Ag_2_∙∙∙**P6Q3** system, in which the Ag ions interact with non-adjacent rings, but now, the remaining aromatic ring (i.e., the one not bound to Ag) rotates slightly (see, e.g., the bottom right ethoxy group in the structure of Figure 4f). Such a rotation increases the stabilization via interactions with one of the CF_3_ groups, while only for **P6Q3,** such a rotation is not hampered by increased steric interactions between, e.g., adjacent ethoxy groups, as—within this series—only this host molecule has no adjacent alkoxy groups. Finally, the interaction strength of the two Ag ions toward the P6 host is quite significant, especially for **EtP6** and **P6Q1**, although it is likely overestimated (as no translation entropy is included in our calculations, which, of course, would promote having two loose entities rather than a bound one).

### 2.5. Specific Binding of 1-Alkynes to Silver-Loaded Pillararenes

Finally, we obtained preliminary data on the complexation of a series of internal and terminal alkenes and alkynes with the silver-loaded cavities of **EtP6** and **P6Q1**. First, we used both standard ^1^H NMR and DOSY NMR to, respectively, confirm that the structure and the potential of **EtP6/P6Q1** and the guest molecule were moving together in the case of complexation. These combined NMR spectra (see Supporting Information Figures S31–S35) display a striking preference for the binding of terminal alkynes (1-heptyne and 1-hexyne as examples), while there is no complexation observable for either internal and terminal alkenes nor for internal alkynes (Figures S36–S38). This exclusive behavior is likely caused by the strong in-cavity complexation of C≡C bonds to Ag^+^ [20,21], while the other molecules (internal alkynes, terminal and internal alkenes) likely due to steric effects within the **EtP6** or **P6Q1** cavity cannot achieve such interactions.

As kindly suggested by a reviewer, we also studied the process using UV-Vis absorption spectroscopy, specifically to find out more about the Ag∙∙∙alkyne interactions. Since there is no peak for the alkyne proton on NMR spectra, it was considered that—rather than peak broadening—it could perhaps be possible that silver σ-binds with alkyne anions by deprotonating the alkyne. This could also explain why no alkene or internal alkyne binding occurs and would potentially suggest that **EtP6**/**P6Q1** is not involved. However, this line of thought is incorrect for two reasons. First, alkyne anions display a maximum in the UV–Vis absorption around 270 nm [34], which is close to the minimum absorption of P6 compounds. The absorption around this minimum is basically unchanged upon the first addition of Ag^+^ to **EtP6** and subsequently the addition of 1-hexyne. In other words, we see no evidence for the formation of the alkyne anion, although future studies would be warranted in which the respective barriers to σ-type or π-type binding of various alkynes with Ag^+^ are investigated. Second, such a deprotonation can be considered generally unlikely, as there is strong evidence from the wide body of argentation chromatography that the Ag^+^∙∙∙alkyne is (reversible) π-binding, rather than (at least partially irreversible) σ-type binding. (If it would be even partially irreversible, it would fully invalidate argentation chromatography for terminal alkynes.) Taken together, we would argue that σ-type binding/alkyne anion formation is unlikely.

**Figure 5:**
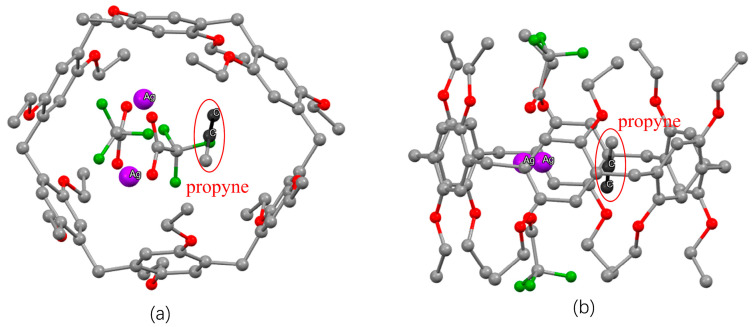
(**a**) Top and (**b**) side views of optimized structures for **EtP6** with two CF_3_COOAg and one propyne in the cavity. Color legend: grey (carbon), red (oxygen), green (fluorine), purple (silver). Hydrogen atoms have been omitted for clarity.

To study the resulting complex in some further detail, we again used wB97XD/def2TZVP optimizations to obtain the binding free energy; here, 1-propyne was chosen for computational efficiency. The optimized structure of **EtP6** with two CF_3_COOAg and one propyne in the cavity (Figure 5) indeed confirms such an Ag^+^∙∙∙alkyne interaction, and yields ∆G = −5.8 kcal/mol for the in-cavity binding of propyne. In other words, the confinement of the silver-loaded cavity provides highly selective Ag∙∙∙alkyne interactions, which—to the best of our knowledge—has not been observed before.

## 3. Materials and Methods

### 3.1. Materials

The experimental procedures employed in this study adhered to rigorous protocols. All initial starting materials, reagents and solvents were procured from established commercial suppliers and were utilized in their as-received state, unless specifically indicated otherwise. The synthesis of compounds including **MeP5**, **EtP5**, **EtP6** and **P6Qns** was conducted following the methods elucidated below and in the Supplementary Information.

#### General Procedures

**Synthesis of MeP5**: To a solution of 1,4-dimethoxybenzene (2.77 g, 20.0 mmol) and paraformaldehyde (0.6 g, 20.0 mmol) in 1,2-dichloroethane (200 mL), trifluoroacetic acid (10 mL) was added. The reaction mixture was refluxed for 2 h. After cooling, the reaction mixture was poured into methanol. The resulting precipitate was collected through filtration. The crude product was subjected to column chromatography purification to afford **MeP5** (2,35 g, 2.13 mmol, 80%) [15].

**Synthesis of EtP5 and EtP6**: A mixture of ferric chloride (FeCl_3_) and choline chloride (ChCl) with a molar ratio of 2:1 was mixed well and heated to 120 °C with stirring until a dark brown liquid formed. To the solution of 1,4-diethoxybenzene (16.6 g, 100 mmol) in dichloromethane (1500 mL), paraformaldehyde (9.0 g, 300 mmol) was added. Then, this solution was added to the dark brown liquid (7.0 g, 15 mmol). The mixture was stirred at 25 °C for one week and quenched by addition of water. The crude product was purified via column chromatography to yield **EtP5** (1.25 g, 1.4 mmol, 7%) and—in larger quantities—**EtP6** (6.2 g, 5.83 mmol, 35%) [17].

**Synthesis of P6Q1**, **P6Q2** and **P6Q3**: To a solution of **EtP6** (1.06 g, 1 mmol) in DCM/THF (100 mL, 9:1, 8:2, 7:3, *v*/*v*, respectively, for the synthesis of **P6Q1**, **P6Q2** and **P6Q3**), an aqueous solution of (NH_4_)_2_[Ce(NO_3_)_6_] (2.2, 4.4 or 6.6 equiv., respectively) in water was added. The resulting red-colored mixture was stirred at room temperature for 30 min to 3 h, washed with water and concentrated under reduced pressure. The crude product was purified via column chromatography to afford **P6Q1** (0.51 g, 0.50 mmol, 50%), **P6Q2** (0.43 g, 0.45 mmol, 45%) or **P6Q3** (0.27 g, 0.30 mmol, 30%) [10].

### 3.2. Stoichiometry Determination

The stoichiometric ratios underlying the complexation between pillar[*n*]arenes and CF_3_COOAg were elucidated using the well-established methodology of Job Plots. To ascertain these ratios, the combined concentration of the host and guest species ([host + guest]) was held constant at 2.0 mM. The host-to-guest ratio was then systematically varied to create a spectrum of ratios, ranging from 9:1 to 1:9. Importantly, the silver ion-pair was treated as a singular guest entity, effectively halving the [Ag^+^] concentration, as each ‘guest’ now comprised two Ag^+^ ions. This reevaluation allowed us to explore the range from 0.2 mM of the Ag_2_^2+^ guest (resulting from 0.4 mM of Ag^+^ in a 1.8 mM pillararene solution) to 1.8 mM of the Ag_2_^2+^ guest (achieved by introducing 3.6 mM of Ag^+^ into a 0.2 mM pillararene solution).

### 3.3. Binding Constants Determination

The quantification of binding constants was facilitated through NMR titration, a robust method employed within the experimental framework. The concentration of the pillararene hosts was consistently maintained at 2.00 mM. The guest concentration, comprising CF_3_COOAg, was meticulously varied across a spectrum encompassing increments of 0.50 mM, spanning from 0 mM to 20.00 mM (0 mM to 0.50 mM, 1.00 mM, 1.50 mM, 2.00 mM, 2.50 mM, 3.00 mM, 3.50 mM, 4.00 mM, 6.00 mM, 8.00 mM, 10.00 mM, 12.00 mM, 14.00 mM, 16.00 mM, 18.00 mM and 20.00 mM). The resulting binding data were subjected to mathematical analysis, employing an equation that effectively models the observed changes in chemical shifts as a function of host and guest concentrations. The *Origin 2016 Pro* software package was used for this analysis. The equation is given below:

Y = Y_0_ + DY × ((K_a_ × (P + x) + 1) − SQRT(((K_a_ × (P + x) + 1)^2^) − 4 × K_a_ × K_a_ × P × x))/(2 × K_a_ × P)

Y = measured chemical shift;

Y_0_ = chemical shift pertaining to the solution devoid of host molecules;

DY = maximal change in chemical shift, encapsulating the difference between a fully occupied host and an unoccupied one;

K_a_ = binding constant;

P = total concentration of host species;

x = cumulative concentration of guest species.

### 3.4. ESI-MS Competition Experiments

In the competitive ESI-MS competition experiments, two distinct host species were combined with two equivalents of the guest, CF_3_COOAg, translating to a concentration of 1.0 mmol/L for each host and 2.0 mmol/L for CF_3_COOAg. The experimental metric employed involved the comparison of NL values derived from the [host + CF_3_COOAg + Ag]^+^ peaks. This analysis enabled the exploration of relative ratios between different peak configurations, thereby offering information regarding the relative binding strengths.

### 3.5. Optimized Geometry Structures of Pillararenes-Ag^+^ and Silver-Loaded-1-Alkyne Complexes

All DFT calculations were carried out using the Gaussian 16 suite of computational programs [35]). The geometries of all stationary points were optimized using the wB97XD hybrid functional using the def2TZVP basis set (in vacuum) [33]. All geometry optimizations were performed without symmetry constraints. Vibrational frequencies were analytically computed at the same level of theory to obtain the Gibbs free energies and to confirm whether the structures were minima. The binding energy of CF_3_COOAg with pillararenes is defined as

$$E_{binding}=E_{\left(P+n*{CF}_{3}COOAg\right)}-E_{P}-{n*E}_{{CF}_{3}COOAg}$$

where $E_{\left(P+n*{CF}_{3}COOAg\right)}$ refers to the free energy of a stable complex of pillararenes and one or two CF_3_COOAg, E_p_ is the free energy of pillararenes and $E_{{CF}_{3}COOAg}$ is the free energy of CF_3_COOAg.

## 4. Conclusions

A series of NMR titration and ESI-MS experiments in combination with high-end DFT data show that the host–guest interactions between pillar[*6*]arene hosts and silver ions are strong, and can be tuned in detail via partial oxidation of the pillararene aromatic rings. Such host–guest, Ag^+^∙∙∙π complexes themselves give rise to the exclusive and unprecedented in-cavity complexation of terminal alkynes over internal alkynes, internal alkenes and terminal alkenes. This sharp distinction provides a novel basis for specific separations of terminal alkynes from other compounds and will be studied further in our laboratories.

## Figures and Tables

**Table 1:** Synthesis of **P6Q1**, **P6Q2** and **P6Q3**.

Main Product	Solvent	Equiv. Oxidant	Reaction Time	Isolated Yield
**P6Q1**	DCM/THF (9:1)	2.2	30 min	50%
**P6Q2**	DCM/THF (8:2)	4.4	3 h	45%
**P6Q3**	DCM/THF (7:3)	6.6	3 h	30%

**Table 2:** Association constants of **MeP5**, **EtP5**, **EtP6** and **P6Q1** with CF_3_COOAg in CDCl_3_:THF-d_8_ = 1:2 at 298 K.

Host	Association Constant/M^−1^
**MeP5**	(1.41 ± 0.04) × 10^3^
**EtP5**	(2.24 ± 0.25) × 10^3^
**EtP6**	(2.04 ± 0.41) × 10^3^
**P6Q1**	(0.66 ± 0.08) × 10^3^

## Data Availability

Not applicable.

## Supplementary Materials

The following supporting information can be downloaded at https://www.mdpi.com/article/10.3390/molecules28207009/s1. Materials and General Methods (Schemes S1–S3, Figure S1–S6) [18,23,24]; Stoichiometry and association constant determination for the complexation between Pillar[*n*]arenes and CF_3_COOAg (Figure S7–S21) [11,22,31,35,36,37,38]; ESI-MS spectra of mixtures of Pillar[*n*]arenes and CF_3_COOAg (Figure S22–S28); Optimized geometry structures of Pillararenes-Ag+ complexes (Table S1, Figures S29 and S30) [33]; Selective binding of terminal alkynes by silver-loaded cavities (Figure S31–S39); Cartesian coordinates and energies of optimized structures.
