# Supplementary material for: Tunable Supramolecular Ag+-Host Interactions in Pillar[n]arene[m]quinones and Ensuing Specific Binding to 1-Alkynes
Source: Molecules. 2023 Oct 10;28(20):7009. doi: 10.3390/molecules28207009 (PMC10609613; doi:10.3390/molecules28207009)
Supplement: Supplementary file 1 [file molecules-28-07009-s001.zip › molecules-2625339-SI.pdf]

Supporting Information

Tunable Supramolecular Ag+-Host Interactions in Pillar[*n*]arene[*m*]quinones and Ensuing Specific Binding to 1-Alkynes

Yumei Zhu [1], Jorge Escorihuela [2], Haiying Wang [1], Andrew C.-H. Sue [3] and Han Zuilhof [1,4,*]

[1] School of Pharmaceutical Science & Technology, Tianjin University, 92 Weijin Road, Nankai District, Tianjin 300072, China; 1019213019@tju.edu.cn (Y.Z.); hywang@nsmc.edu.cn (H.W.)
[2] Departamento de Química Orgánica, Universitat de València, Avda, Vicente Andrés Estellés s/n, 46100 València, Spain; jorge.escorihuela@uv.es
[3] College of Chemistry and Chemical Engineering, Xiamen University , 422 Siming South Road, Siming District, Xiamen 361005, China; andrewsue@xmu.edu.cn
[4] Laboratory of Organic Chemistry, Wageningen University, Stippeneng 4, 6708 WE Wageningen, The Netherlands
* Correspondence: han.zuilhof@wur.nl

Table of Contents

1. Materials and General Methods P1

2. Stoichiometry and association constant determination for the complexation between Pillar[*n*]arenes and CF_3COOAg P7

3. ESI-MS spectra of mixtures of Pillar[*n*]arenes and CF_3COOAg........................P21

4. Optimized geometry structures of Pillararenes-Ag^+ complexes P28

5. Selective binding of terminal alkynes by silver-loaded cavities.........................P33

6. Cartesian coordinates and energies of optimized structures P41

7. References ………………………………………………………………… P92

1. Materials and General Methods

Materials: All starting materials, reagents, and solvents were purchased from commercial vendors and used as received, unless otherwise noted. Compounds **MeP5**, **EtP5**, **EtP6** and **P6Qns** were synthesized as specified per compound below. Analytical thin-layer chromatography (TLC) was performed on aluminum sheets, precoated with silica gel GF_{254}. Flash column chromatography was performed over silica gel (200−300 mesh).

Methods: All NMR data were recorded on a Bruker Advance 400 MHz spectrometer at room temperature, unless otherwise noted. ESI-MS measurements were performed on a Q Exactive™ HF/UltiMate™ 3000 RSLCnano at 298 K.

Synthetic Procedures:

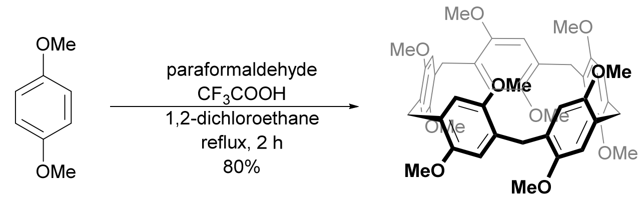

Scheme S1. Synthetic route of **MeP5**.

MeP5: To a solution of 1,4-dimethoxybenzene (2.77 g, 20 mmol) and paraformaldehyde (0.6 g, 20 mmol) in 1,2-dichloroethane (200 mL), trifluoroacetic acid (10 mL) was added. The reaction mixture was refluxed for 2 h. After cooling, the reaction mixture was poured into methanol. The resulting precipitate was collected by filtration. The crude product was subjected to column chromatography (*n*-hexane/EtOAc = 96:4 to 80:20, v/v) to afford **MeP5** (2.35 g, 3.13 mmol, 80%). The ^{1}H and ^{13}C NMR data (**Figure S1**) were in accordance with literature.[1]

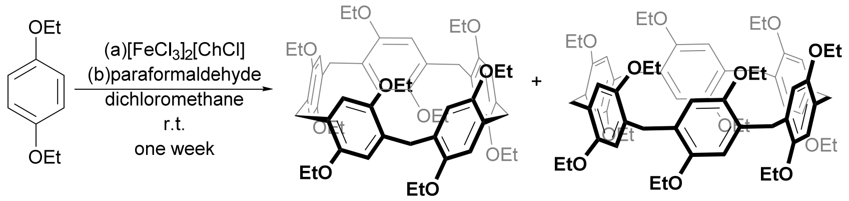

Scheme S2. Synthetic route of **EtP5** and **EtP6**.

EtP5 and **EtP6**: A mixture of the ferric chloride ($FeCl_3$) and choline chloride (ChCl) with a molar ratio of 2:1 was mixed well and heated to 120 °C with stirring until a dark brown liquid formed. To the solution of 1,4-diethoxybenzene (16.6 g, 100 mmol) in dichloromethane (1500 mL) was added paraformaldehyde (9.0 g, 300 mmol). Then this solution was added to the dark brown liquid (7.0 g, 15 mmol). The mixture was

stirred at 25 °C for one week, and quenched by addition of water. The organic phase was separated and washed with saturated aqueous $NaHCO_3$, H_2O and brine. The crude product was purified by column chromatography to yield **EtP5** (1.25 g, 1.4 mmol, 7%) and **EtP6** (6.20 g, 5.83 mmol, 35%) (*n*-hexane/EtOAc = 96:4 to 80:20, v/v). The ^{1}H and ^{13}C NMR data (**Figure S2&S3**) were in accordance with literature.[2]

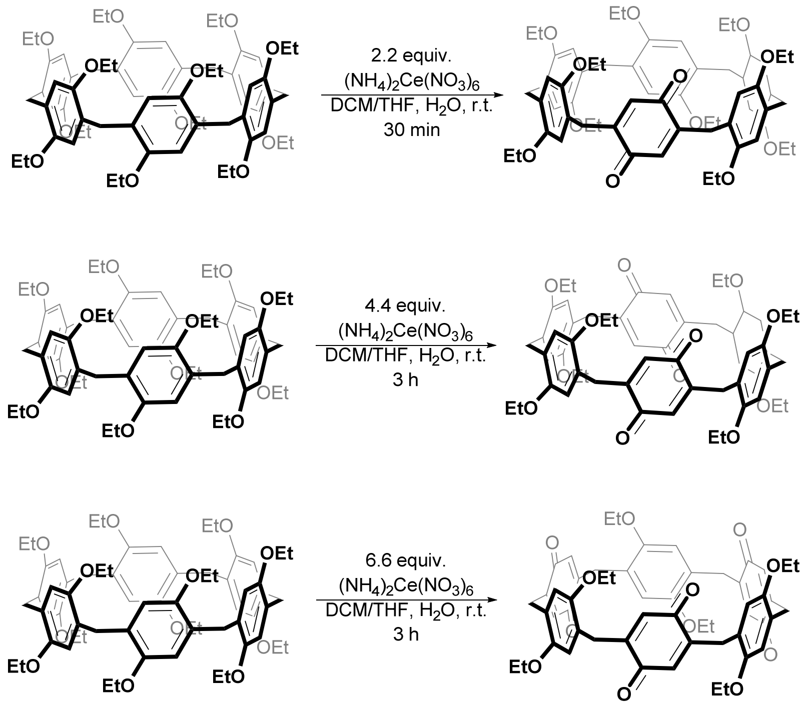

Scheme S3. Synthetic route towards **P6Qns**.

P6Q1 (pillar[6]arene[1]quinone): To a solution of **EtP6** (1.06 g, 1 mmol) in DCM/THF (100 mL, 9:1, v/v), an aqueous solution of $(NH_4)_2[Ce(NO_3)_6]$ (1.33 g, 2.2 mmol) in 10 mL water was added. The resulting red-colored mixture was stirred at room temperature for 30 min, washed with water (3 × 100 mL), and concentrated under reduced pressure. The crude product was purified by column chromatography ((*n*-hexane/EtOAc = 9:1, v/v) to afford **P6Q1** product as a red solid (0.51 g, 0.50 mmol, 50%). The ^{1}H and ^{13}C NMR data (**Figure S4**) is in accordance with literature.[3]

P6Q2 (pillar[6]arene[2]quinone): To a solution of **EtP6** (1.06 g, 1 mmol) in DCM/THF (100 mL, 8:2, v/v), an aqueous solution of $(NH_4)_2[Ce(NO_3)_6]$ (2.66 g, 4.4 mmol) in 20 mL water was added. The resulting red-colored mixture was stirred at room temperature for 3 h, washed with water (3 × 100 mL), and concentrated under reduced pressure. The crude product was purified by column chromatography ((*n*-hexane/EtOAc = 9:1, v/v) to afford **P6Q2** product as a red solid (0.43 g, 0.45 mmol, 45%). The ^{1}H and ^{13}C NMR data (**Figure S5**) is in accordance with literature.[3]

P6Q3 (pillar[6]arene[3]quinone): To a solution of **EtP6** (1.06 g, 1 mmol) in DCM/THF (100 mL, 7:3, v/v), an aqueous solution of $(NH_4)_2[Ce(NO_3)_6]$ (3.99 g, 6.6 mmol) in 30 mL water was added. The resulting red-colored mixture was stirred at room temperature for 3 h, washed with water (3 × 100 mL), and concentrated under reduced pressure. The crude product was purified by column chromatography ((*n*-hexane/EtOAc = 9:1, v/v) to afford **P6Q3** product as a red solid (0.27 g, 0.30 mmol, 30%). The ^{1}H and ^{13}C NMR data (**Figure S6**) is in accordance with literature.[3]

Note: the solvent ratios DCM/THF were optimized to balance the solubility of all the reactants, and yielded the high amount of products.

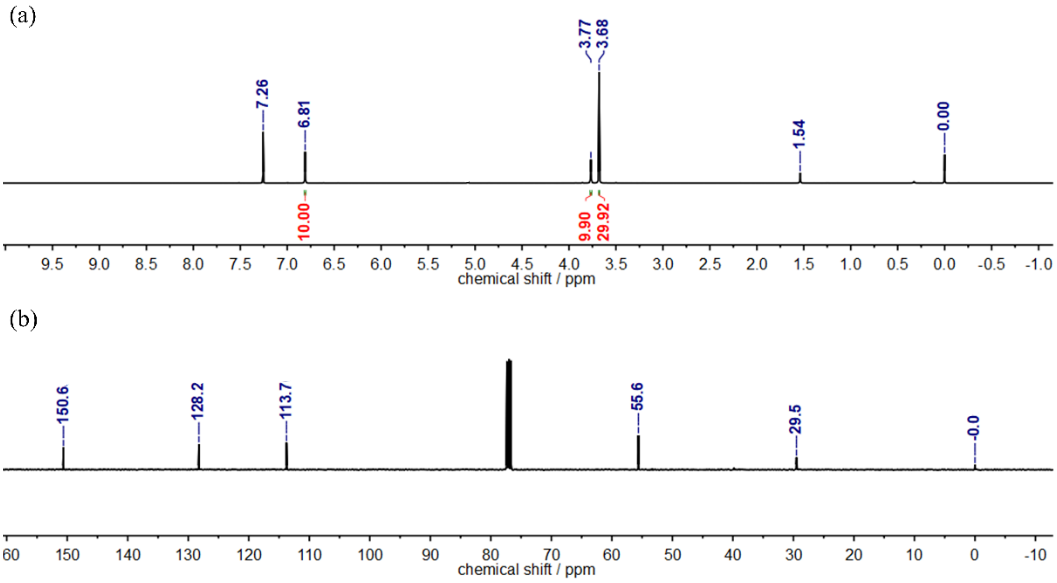

Figure S1. Partial ^{1}H and ^{13}C NMR spectra (400 MHz, $CDCl_3$, room temperature) of **MeP5**.

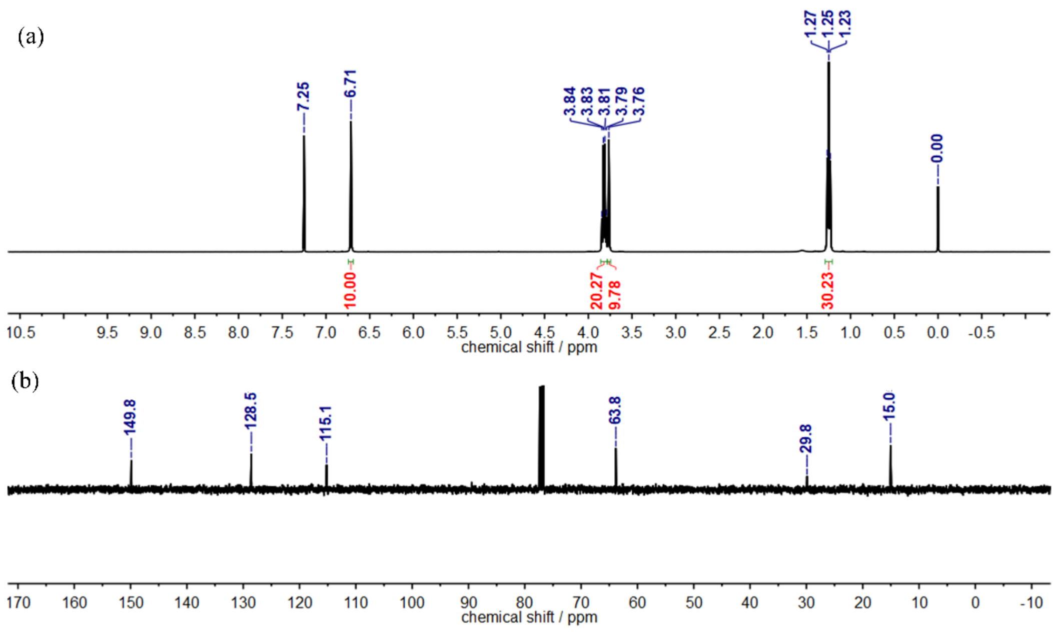

Figure S2. Partial ^{1}H and ^{13}C NMR spectra (400 MHz, $CDCl_3$, room temperature) of **EtP5**.

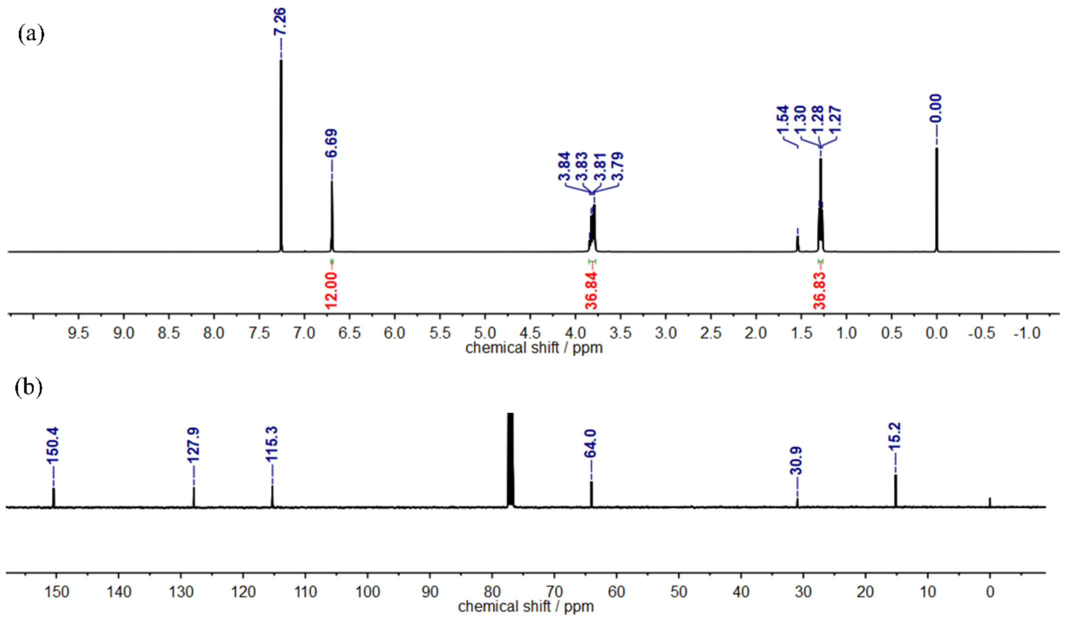

Figure S3. Partial ^{1}H and ^{13}C NMR spectra (400 MHz, $CDCl_3$, room temperature) of **EtP6**.

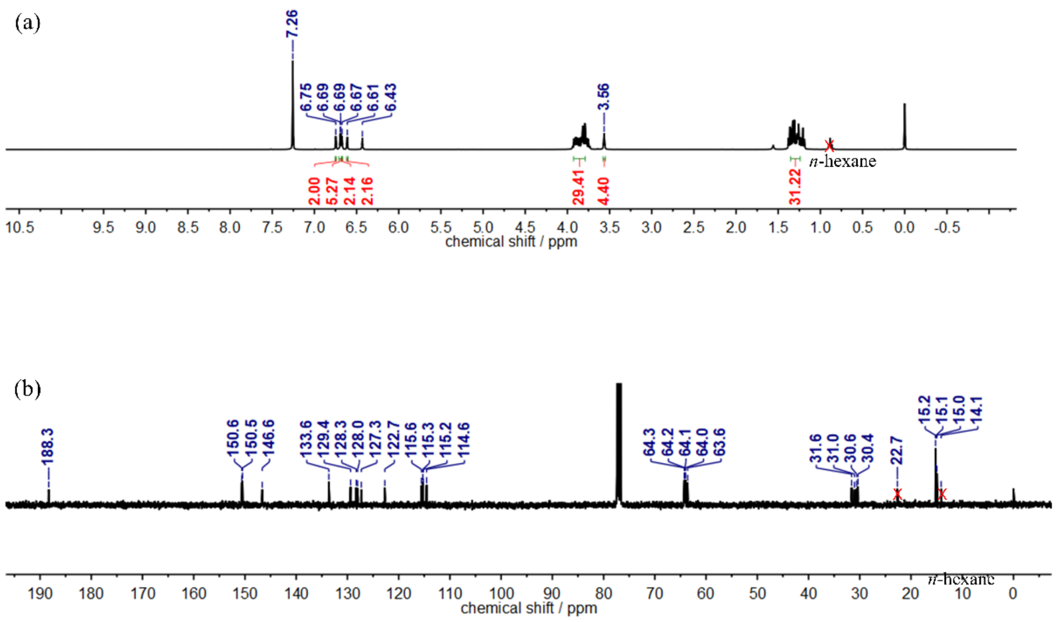

Figure S4. Partial ^{1}H and ^{13}C NMR spectra (400 MHz, $CDCl_3$, room temperature) of **P6Q1**.

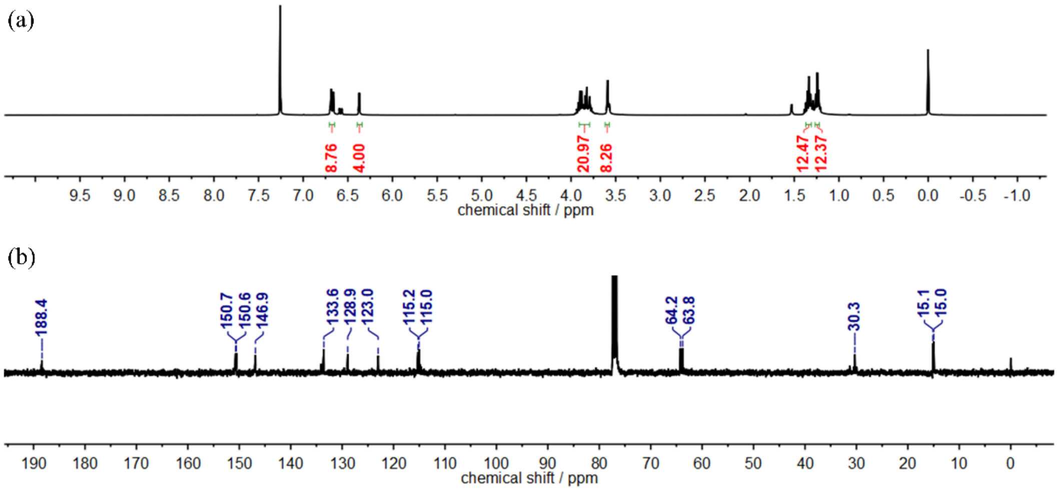

Figure S5. Partial ^{1}H and ^{13}C NMR spectra (400 MHz, $CDCl_3$, room temperature) of **P6Q2**.

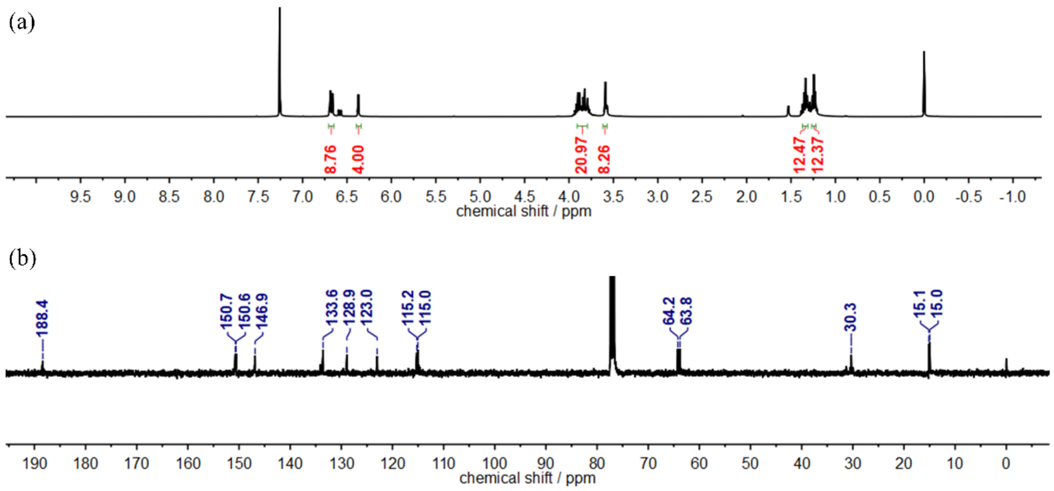

Figure S6. Partial ^{1}H and ^{13}C NMR spectra (400 MHz, $CDCl_3$, room temperature) of **P6Q3**.

2. Stoichiometry and association constant determination for the complexation between Pillar[n]arenes and CF_3COOAg

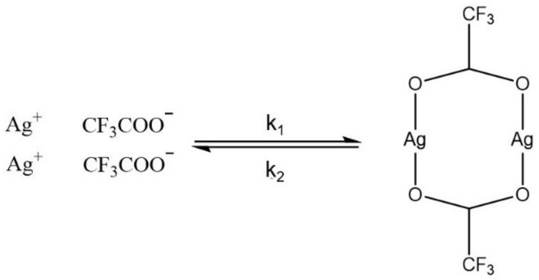

Figure S7. The possible equilibria existing in silver ion-pair recognition system.[4]

Job plots are – within limitations [5-7] – useful to determine the stoichiometry of the host-guest complexes (generally: it works well if only one type of complex is present, but is difficult to interpret if multiple different complexes are present). Here, the Job

plot and the reported crystal structures of P5s and P6s with 2 CF_3COOAg [4,8] were combined to obtain the stoichiometry.

The method of Job plot was used to determine the stoichiometry of complexation of Pillar[n]arenes and CF_3COOAg (**Figure S8-S11).** In this, we fixed the total concentration of [host + guest] at 2.0 mM, and varied the host/guest concentration to yield different ratios (host/guest) as 9:1 (pillararene : Ag^+ = 1.8 mM : 0.2 mM), 8:2 (pillararene : Ag^+ = 1.6 mM : 0.4 mM), 7:3 (pillararene : Ag^+ = 1.4 mM : 0.6 mM), 6:4 (pillararene : Ag^+ = 1.2 mM : 0.8 mM), 5:5 (pillararene : Ag^+ = 1.0 mM : 1.0 mM), 4:6 (pillararene : Ag^+ = 0.8 mM : 1.2 mM), 3:7 (pillararene : Ag^+ = 0.6 mM : 1.4 mM), 2:8 (pillararene : Ag^+ = 0.4 mM : 1.6 mM) and 1:9 (pillararene : Ag^+ = 0.2 mM : 1.8 mM). From **Figure S8-S11** (left parts of these graphs) the highest y value in these figures are all found around X_{host} is 0.3, which indicates that **MeP5**, **EtP5**, **EtP6** and **P6Q1** each form complexes to CF_3COOAg with the ratio of 1:2 (X_{host} = 0.33).

To make sure that we can indeed use the Job plot as indicator of the stoichiometry of the complex, we treated the silver ion-pair as one guest (right parts of **Figure S8-S11**). In other words: the guest concentration is now half of the [Ag^+], as only 2 Ag^+ ions are considered to be one guest. For this, we then again systematically varied the host/guest ratios from 9:1 to 1:9, i.e., from 1.8 mM pillararene & 0.4 mM of Ag^+ (yielding: 0.2 mM of the Ag_2^{2+} guest) to 0.2 mM pillararene & 3.6 mM of Ag^+ (yielding: 1.8 mM of the Ag_2^{2+} guest). This yielded the right parts in **Figure S8-S11**), and these figures indicate that **MeP5**, **EtP5**, **EtP6** and **P6Q1** each complex to the silver ion pair guest Ag_2^{2+} in a ratio of 1:1.

This set of experiments thus confirms the 1:2 ratio of the pillararenes : Ag^+.

For **P6Q2** and **P6Q3** (**Figure S12&S13**), there is no chemical shift in ^{1}H NMR spectra for any of the protons after adding excess CF_3COOAg.

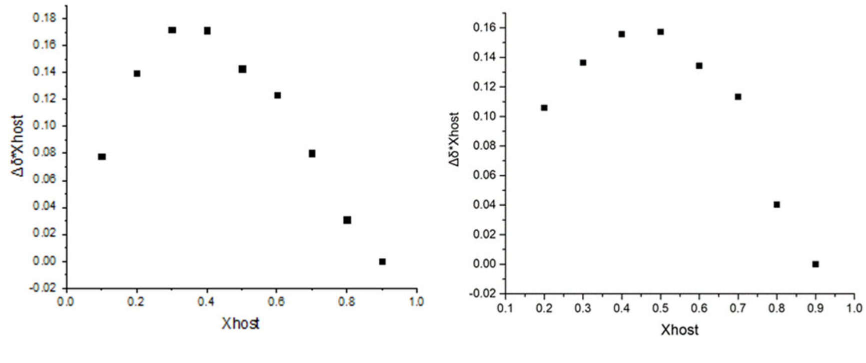

Figure S8. Job plot analysis of CF_3COOAg and **MeP5** (left) and silver ion-pair ($2CF_3COOAg$) and **MeP5** (right).

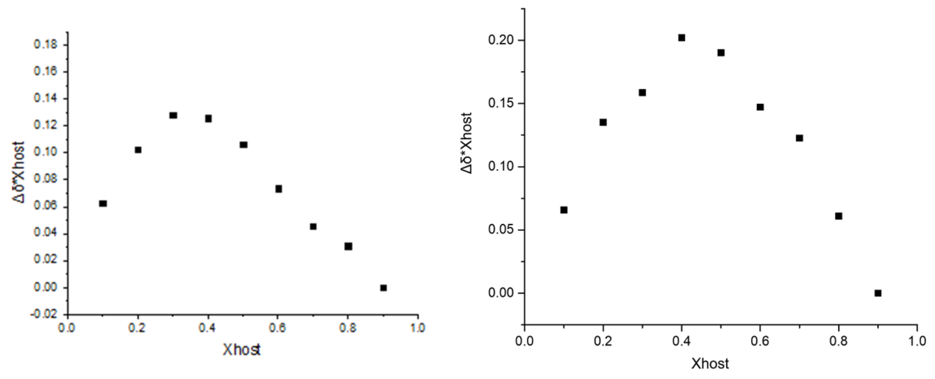

Figure S9. Job plot analysis of CF_3COOAg and **EtP5** (left) and silver ion-pair ($2CF_3COOAg$) and **EtP5** (right).

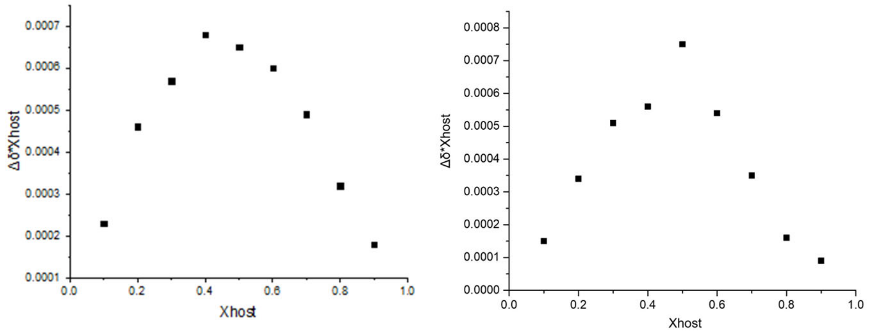

Figure S10. Job plot analysis of CF_3COOAg and **EtP6** (left) and silver ion-pair ($2CF_3COOAg$) and **EtP6** (right).

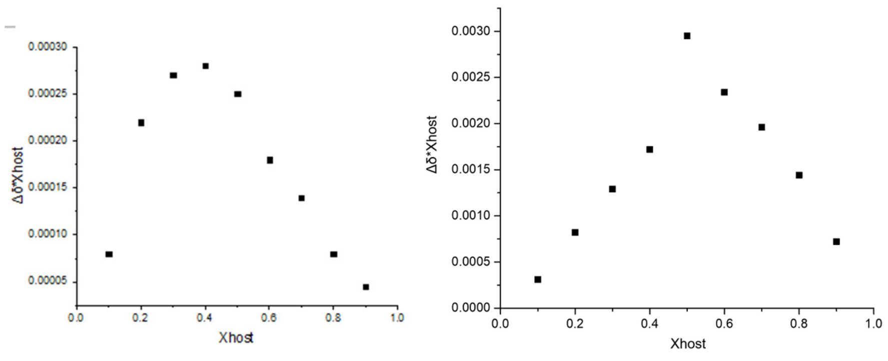

Figure S11. Job plot analysis of CF_3COOAg and **P6Q1** (left) and silver ion-pair ($2CF_3COOAg$) and **P6Q1** (right).

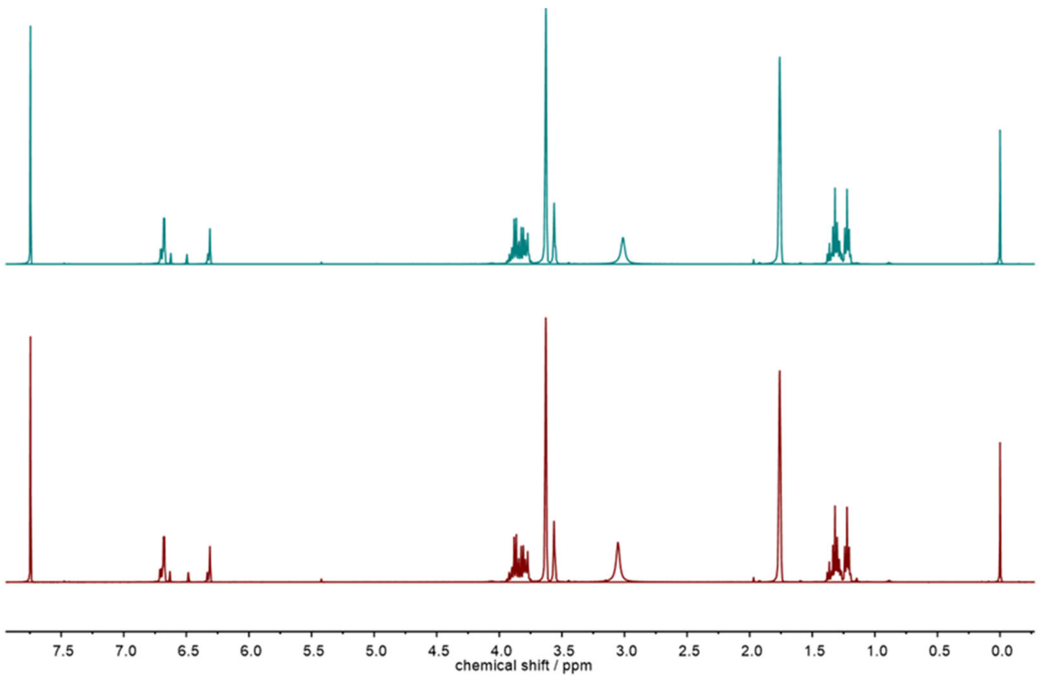

Figure S12. Partial ^{1}H NMR spectra (400 MHz, $CDCl_3$: THF-d_8 = 1:2, v/v, room temperature) of **P6Q2** at a concentration of 2.00 mM (top) and 2.00 mM **P6Q2** with 10.0 mM CF_3COOAg (bottom).

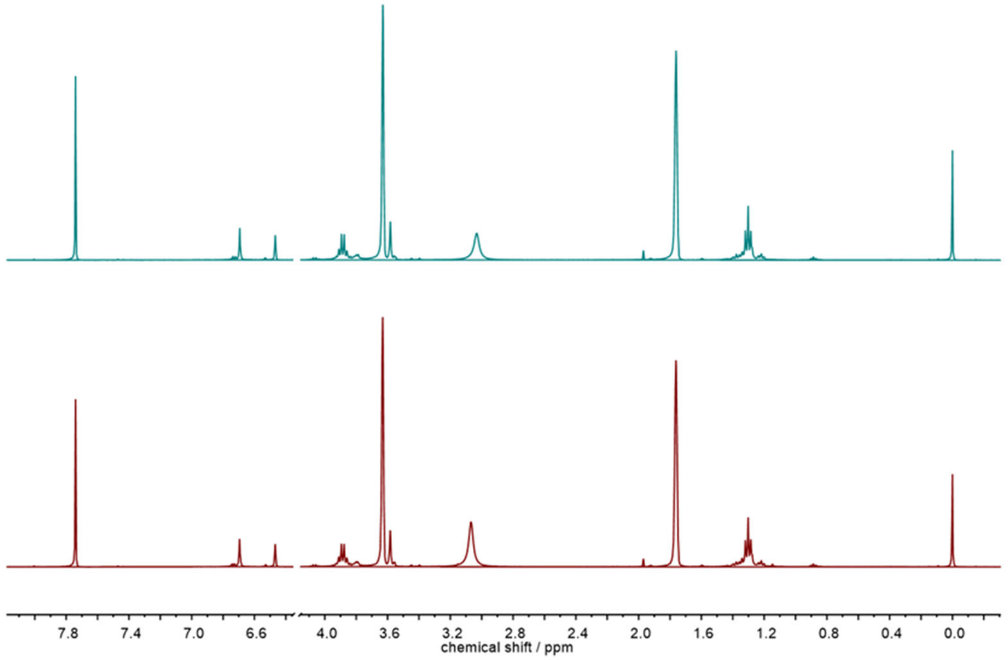

Figure S13. Partial ^{1}H NMR spectra (400 MHz, $CDCl_3$: THF-d_8 = 1:2, v/v, room temperature) of **P6Q3** at a concentration of 2.00 mM (top) and 2.00 mM **P6Q3** with 10.0 mM CF_3COOAg (bottom).

An NMR titration method was used to obtain the binding constants.[9] Consider the equilibrium between the host H and a guest G that can form host-guest complex HG:

$$\mathrm{H} + \mathrm{G} \leftrightarrows \mathrm{HG} \quad \text{(S1)}$$

The equilibrium binding constant K for this equilibrium is defined as:

$$\mathrm{K} = \frac{[HG]}{[H][G]} \quad \text{(S2)}$$

From S2, the equilibrium concentration of the host-guest complex [HG] can be expressed as:

$$[\mathrm{HG}] = \mathrm{K}\,[\mathrm{H}][\mathrm{G}] \quad \text{(S3)}$$

The mass balance for the total guest ($[G]_0$) and total host ($[H]_0$) concentration can be written as:

$$[\mathrm{G}]_0 = [\mathrm{G}] + [\mathrm{HG}] = [\mathrm{G}]\ \mathrm{K}[\mathrm{H}][\mathrm{G}] \quad \text{(S4)}$$

$$[\mathrm{H}]_0 = [\mathrm{H}] + [\mathrm{HG}] = [\mathrm{H}]\ \mathrm{K}[\mathrm{H}][\mathrm{G}] = [\mathrm{H}]\ \mathrm{x}\ (1 + \mathrm{K}[\mathrm{G}]) \quad \text{(S5)}$$

The fraction of occupied host could be defined as：

$$\mathrm{y} = \frac{[HG]}{[H]_0} = \frac{K[H][G]}{[H]\times(1+K[G])} = \frac{K[G]}{1+K[G]} \quad \text{(S6)}$$

Equation S6 could be used to determine the binding constant K if the fraction of occupied host is known as a function of free guest concentration at equilibrium ([G]). From S3 and S4, this free guest concentration at equilibrium is equal to:

$$[G] = [G]_0 - [HG] = [G]_0 - \mathrm{K}[\mathrm{H}][G] \quad \text{(S7)}$$

From S5 and S6, the free host concentration at equilibrium is equal to:

$$[\mathrm{H}] = (1\text{-y})[\mathrm{H}]_0 \quad \text{(S8)}$$

S7 and S8 were combined to give：

$$[G] = [G]_0 - \mathrm{K}(1\text{-y})[\mathrm{H}]_0[G] \quad \text{(S9)}$$

Rearranging S9 gives:

$$(1 + K(1 - y)[H]_0 \times [G] = [G]_0 \qquad (S10)$$

From S10， the free guest concentration at equilibrium is equal to:

$$[G] = \frac{[G]_0}{1 + K(1 - y)[H]_0} \qquad (S11)$$

Rearranging S6 gives:

$$y + yK[G] = K[G] \qquad (S12)$$

Mixing S11 and S12 yields:

$$y + yK\left(\frac{[G]_0}{1 + K(1 - y)[H]_0}\right) = K\left(\frac{[G]_0}{1 + K(1 - y)[H]_0}\right) \qquad (S13)$$

Rearranging S13 yields:

$$y(1 + K(1 - y)[H]_0) + yK[G]_0 = K[G]_0 \qquad (S14)$$

Rearranging S4 gives:

$$y(1 + K[H]_0 - Ky[H]_0) + yK[G]_0 - K[G]_0 = 0 \qquad (S15)$$

Rearranging S15 gives:

$$-K[H]_0y^2 + (1 + K[H]_0 + [G]_0))y - K[G]_0 = 0 \qquad (S16)$$

S16 could be rewritten as:

$$K[H]_0y^2 - (1 + K[H]_0 + [G]_0))y + K[G]_0 = 0 \qquad (S17)$$

Equation S17 is a quadratic equation in y, which can be solved yielding two roots:

$$y = \frac{[1 + K([H]_0 + [G]_0)] \pm \sqrt{[1 + K([H]_0 + [G]_0)]^2 - 4K^2[H]_0[G]_0}}{2K[H]_0} \qquad (S18)$$

From the two roots of S18, it was found that the physically meaningful root is given by:

$$y = \frac{[1+K([H]_0+[G]_0)]-\sqrt{[1+K([H]_0+[G]_0)]^2-4K^2[H]_0[G]_0}}{2K[H]_0} \qquad \text{(S19)}$$

Equation S19 was used to fit the binding data, using Origin. So S19 was adapted to give:

Y =

$$Y_0 + DY \times ((Ka \times (P+x)+1) \text{-} SQRT(((Ka \times (P+x)+1)\,\hat{}\,2) - 4 \times Ka \times Ka \times P \times x)) / (2 \times Ka \times P)$$

(S20)

Y Measured chemical shift

Y_0 Chemical shift of empty host solution

DY Maximal change in chemical shift: the difference in chemical shift of a fully occupied host and an empty host

Ka Binding constant

P Total host concentration

x Total guest concentration

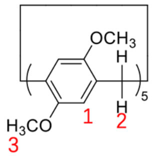

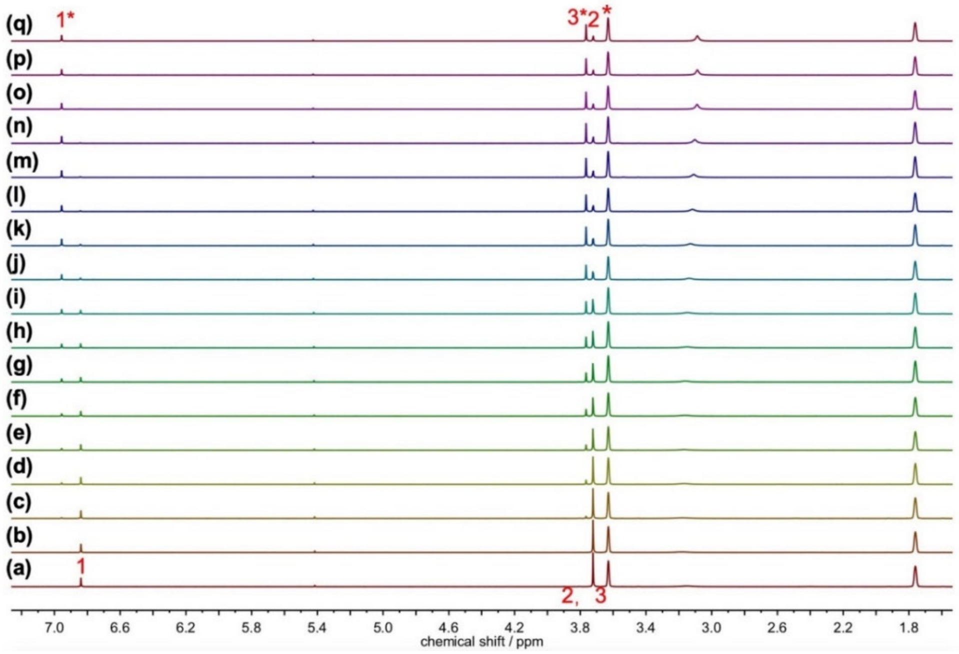

Figure S14. Partial ^{1}H NMR spectra (400 MHz, $CDCl_3$: THF-d_8 = 1:2, v/v, room temperature) of **MeP5** at a concentration of 2.00 mM with different concentrations of CF_3COOAg: (a) 0 mM, (b) 0.50 mM, (c) 1.00 mM, (d) 1.50 mM, (e) 2.00 mM, (f) 2.50 mM, (g) 3.00 mM, (h) 3.50 mM, (i) 4.00 mM, (j) 6.00 mM, (k) 8.00 mM, (l) 10.00 mM, (m) 12.00 mM, (n) 14.00 mM, (o) 16.00 mM, (p) 18.00 mM and (q) 20.00 mM.

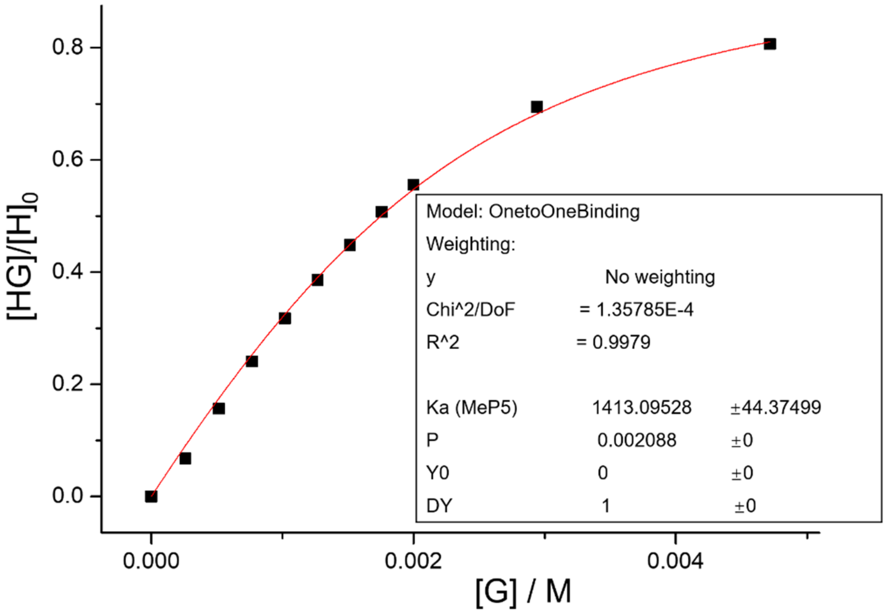

Figure S15. Data fitting for the titration of the slow exchanging $[CF_3COOAg]_2$ guest into a solution of **MeP5**. A binding constant of $1.41 \times 10^3 \pm 0.04 \times 10^3$ M^{-1} was determined for $[CF_3COOAg]_2 \subset$ **MeP5**.

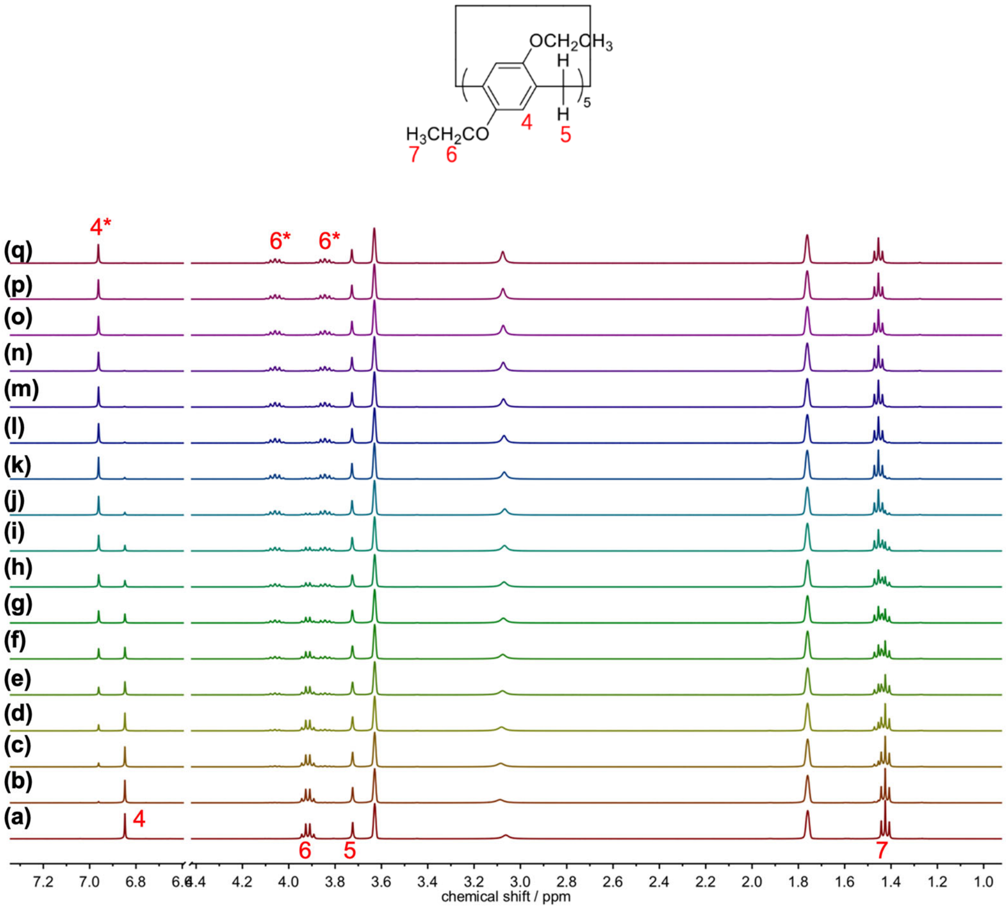

Figure S16. Partial ^{1}H NMR spectra (400 MHz, $CDCl_3$: THF-d_8 = 1:2, v/v, room temperature) of **EtP5** at a concentration of 2.00 mM with different concentrations of CF_3COOAg: (a) 0 mM, (b) 0.50 mM, (c) 1.00 mM, (d) 1.50 mM, (e) 2.00 mM, (f) 2.50 mM, (g) 3.00 mM, (h) 3.50 mM, (i) 4.00 mM, (j) 6.00 mM, (k) 8.00 mM, (l) 10.00 mM, (m) 12.00 mM, (n) 14.00 mM, (o) 16.00 mM, (p) 18.00 mM and (q) 20.00 mM.

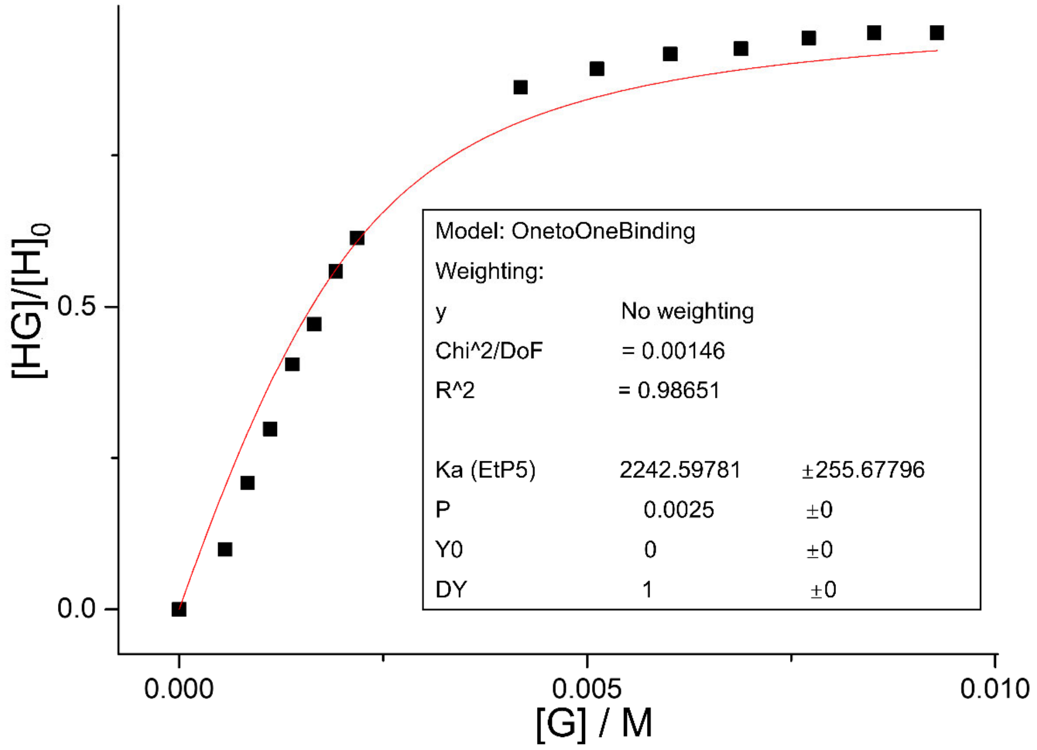

Figure S17. Data fitting for the titration of the slow exchanging $[CF_3COOAg]_2$ guest into a solution of **EtP5**. A binding constant of 2.24 × 10^3 ± 0.25 × 10^3 M^{-1} was determined for $[CF_3COOAg]_2 \subset$ **EtP5**.

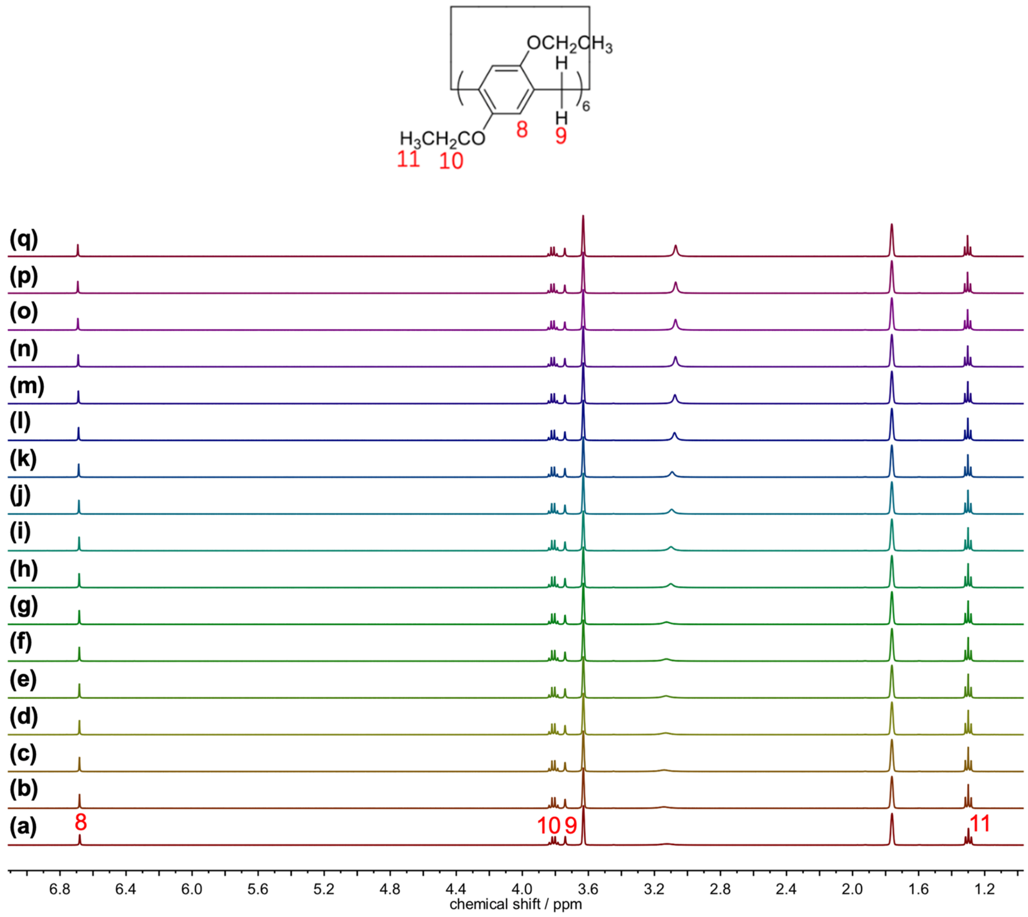

Figure S18. Partial ^{1}H NMR spectra (400 MHz, $CDCl_3$: THF-d_8 = 1:2, v/v, room temperature) of **EtP6** at a concentration of 2.00 mM with different concentrations of CF_3COOAg: (a) 0 mM, (b) 0.50 mM, (c) 1.00 mM, (d) 1.50 mM, (e) 2.00 mM, (f) 2.50 mM, (g) 3.00 mM, (h) 3.50 mM, (i) 4.00 mM, (j) 6.00 mM, (k) 8.00 mM, (l) 10.00 mM, (m) 12.00 mM, (n) 14.00 mM, (o) 16.00 mM, (p) 18.00 mM and (q) 20.00 mM.

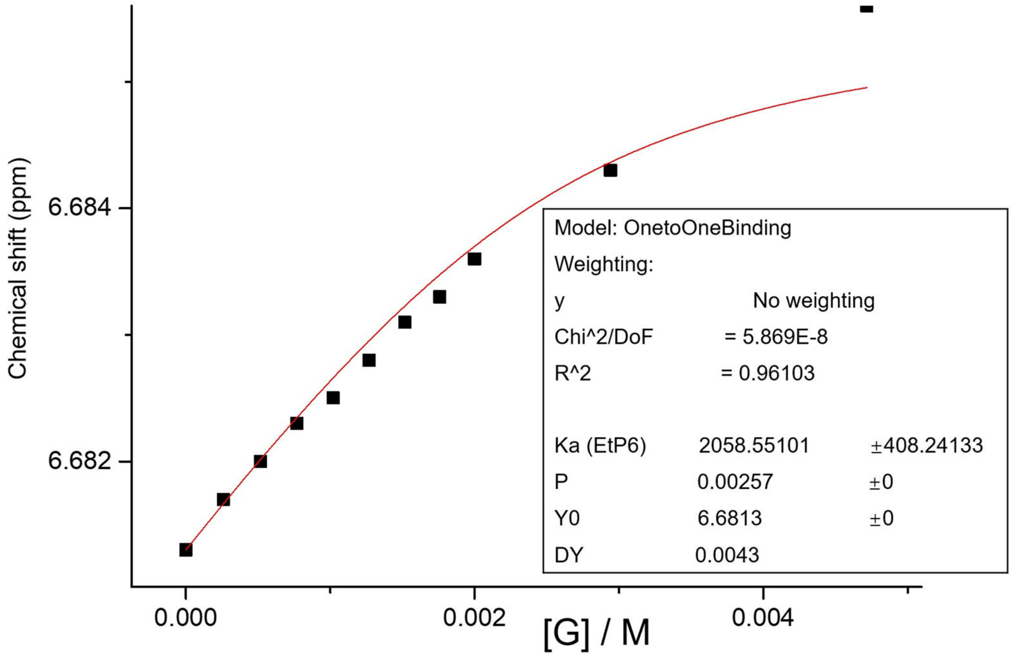

Figure S19. Data fitting for the titration of the fast exchanging $[CF_3COOAg]_2$ guest into a solution of **EtP6**. A binding constant of $2.06 \times 10^3 \pm 0.41 \times 10^3$ M^{-1} was determined for $[CF_3COOAg]_2 \subset$ **EtP6**.

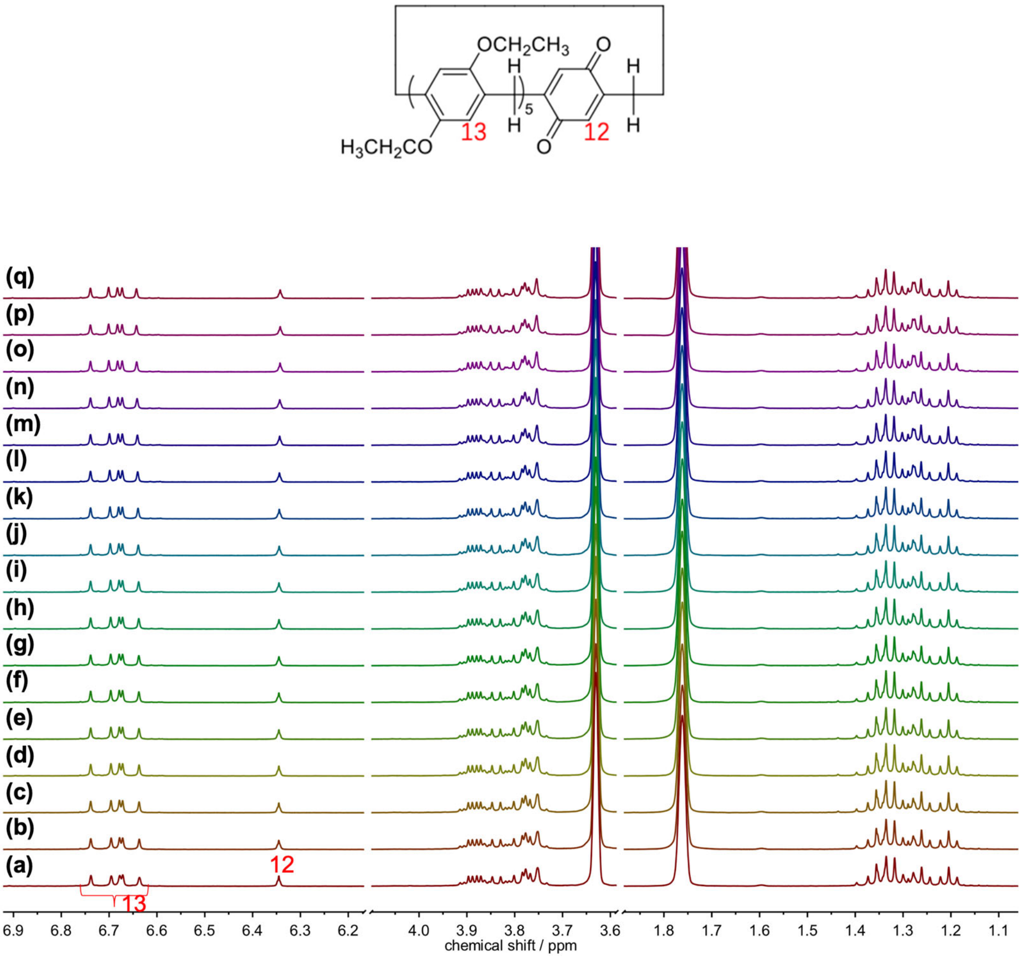

Figure S20. Partial ^{1}H NMR spectra (400 MHz, $CDCl_3$: THF-d_8 = 1:2, v/v, room temperature) of **P6Q1** at a concentration of 2.00 Mm with different concentrations of CF_3COOAg: (a) 0 mM, (b) 0.50 mM, (c) 1.00 mM, (d) 1.50 mM, (e) 2.00 mM, (f) 2.50 mM, (g) 3.00 mM, (h) 3.50 mM, (i) 4.00 mM, (j) 6.00 mM, (k) 8.00 mM, (l) 10.00 mM, (m) 12.00 mM, (n) 14.00 mM, (o) 16.00 mM, (p) 18.00 mM and (q) 20.00 mM.

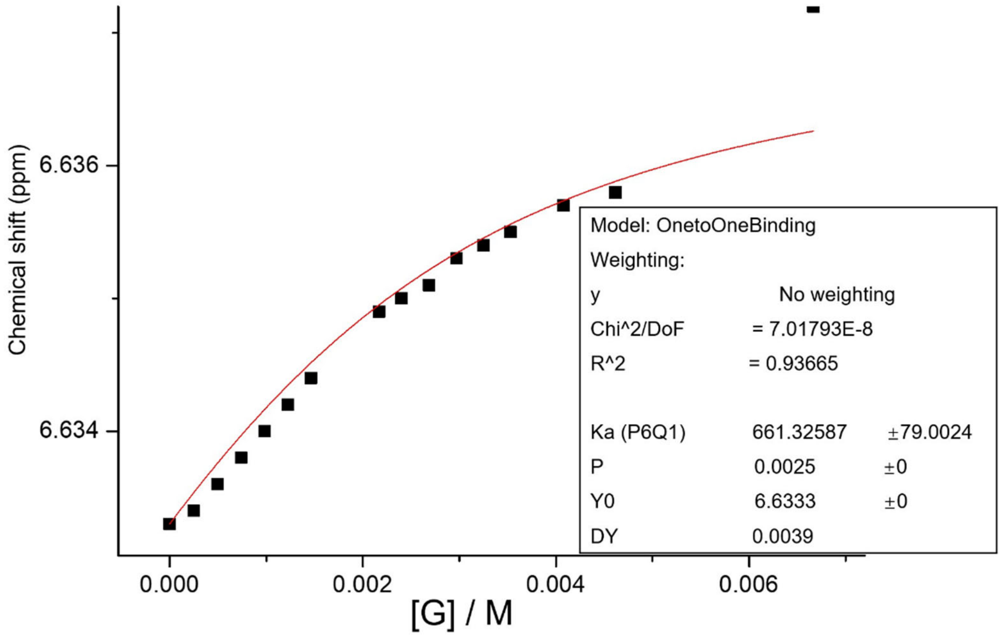

Figure S21. Data fitting for the titration of the fast exchanging $[CF_3COOAg]_2$ guest into a solution of **P6Q1**. A binding constant of $6.61 \times 10^2 \pm 0.79 \times 10^2$ M^{-1} was determined for $[CF_3COOAg]_2 \subset$ **P6Q1**.

3. ESI-MS spectra of mixtures of Pillar[*n*]arenes and CF_3COOAg

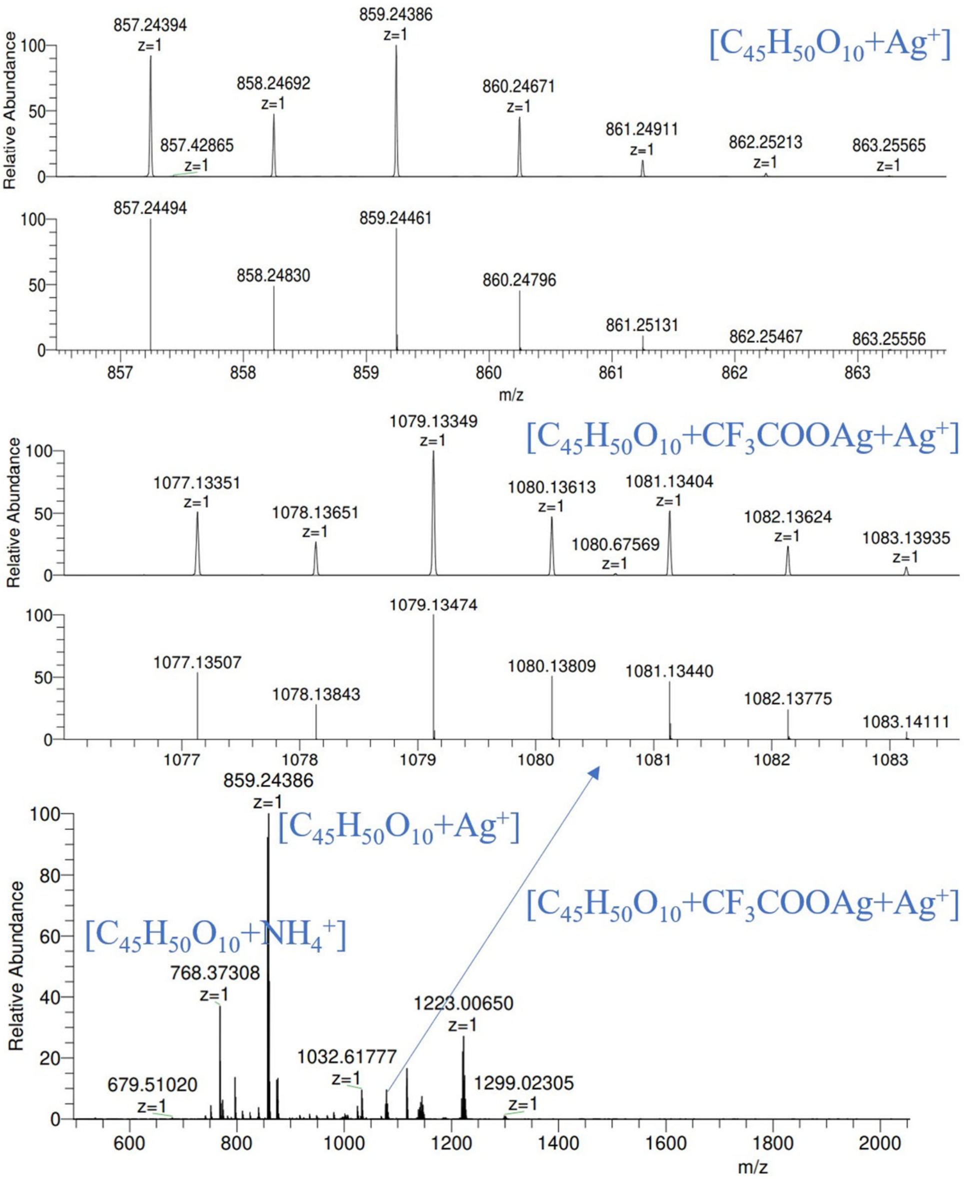

Figure S22. Electrospray ionization mass spectrum of **MeP5** and CF_3COOAg (molar ratio: 1/1). Assignment of main peaks: *m*/*z* 768.37 [**MeP5** + NH_4]$^+$; *m*/*z* 859.24 [**MeP5** + Ag]$^+$; 1079.13 [**MeP5** + CF_3COOAg + Ag]$^+$.

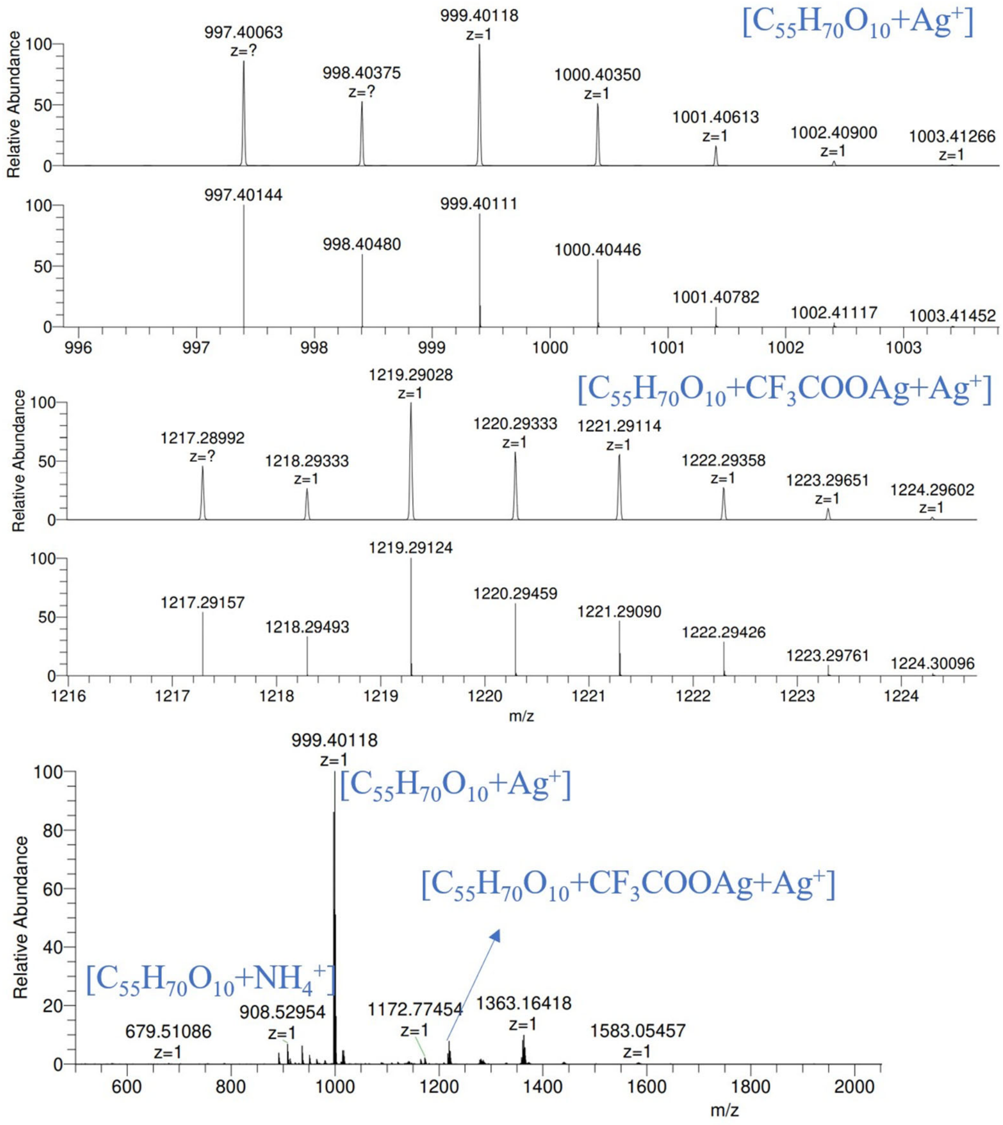

Figure S23. Electrospray ionization mass spectrum of **EtP5** and CF_3COOAg (molar ratio: 1/1). Assignment of main peaks: *m*/*z* 908.53 [**EtP5** + NH_4]$^+$; *m*/*z* 999.40 [**EtP5** + Ag]$^+$; 1219.29 [**EtP5** + CF_3COOAg + Ag]$^+$.

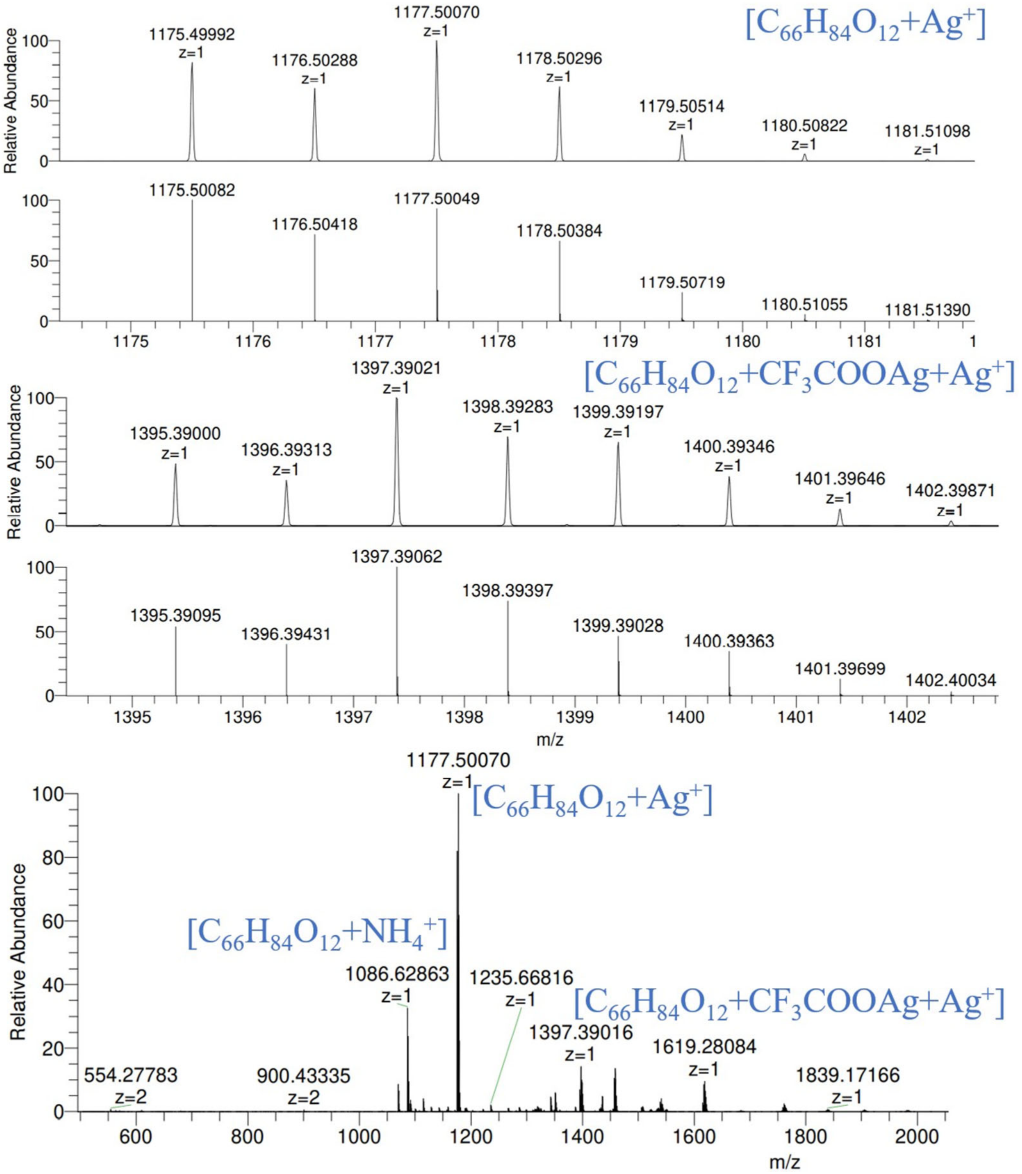

Figure S24. Electrospray ionization mass spectrum of **EtP6** and CF_3COOAg (molar ratio: 1/1). Assignment of main peaks: *m/z* 1086.63 [**EtP6** + NH_4]$^+$; *m/z* 1177.50 [**EtP6** + Ag]$^+$; 1397.39 [**EtP6** + CF_3COOAg + Ag]$^+$.

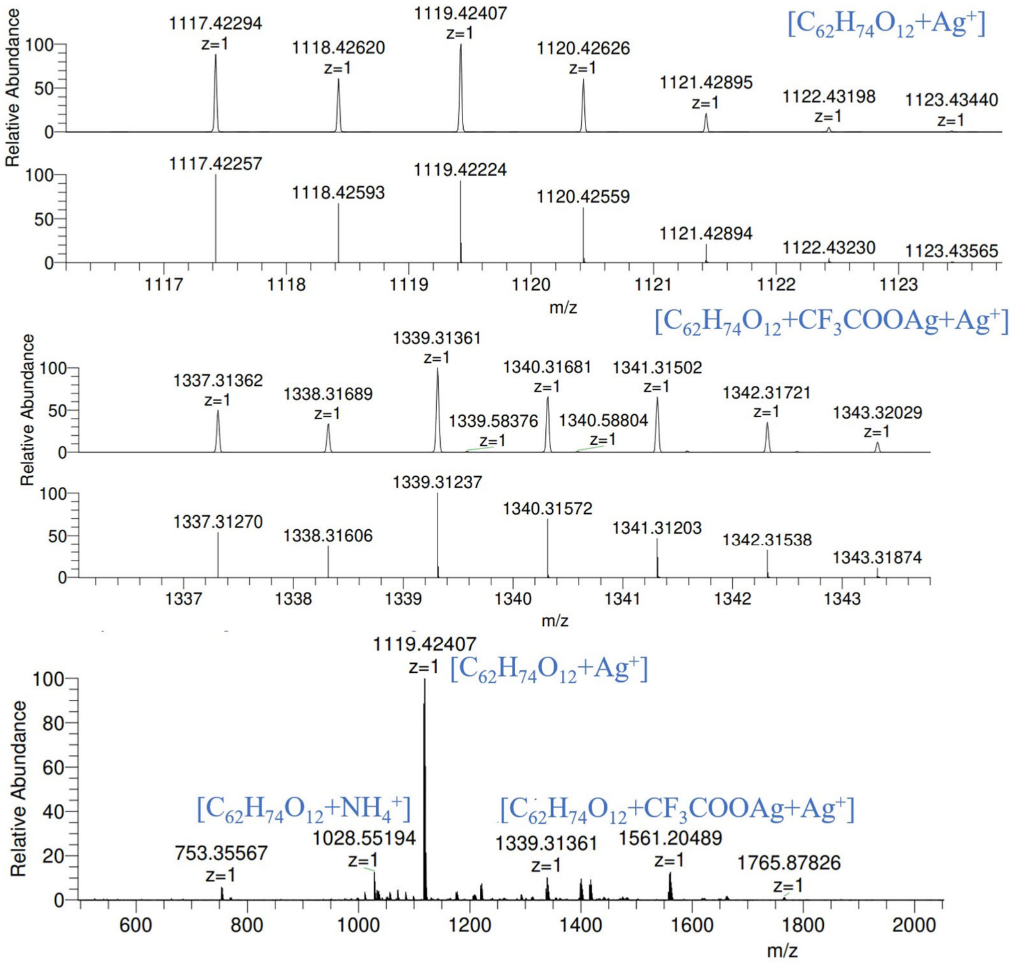

Figure S25. Electrospray ionization mass spectrum of **P6Q1** and CF_3COOAg (molar ratio: 1/1). Assignment of main peaks: *m*/*z* 1028.55 [**P6Q1** + NH_4]$^+$; *m*/*z* 1119.42 [**P6Q1** + Ag]$^+$; 1339.31 [**P6Q1** + CF_3COOAg + Ag]$^+$.

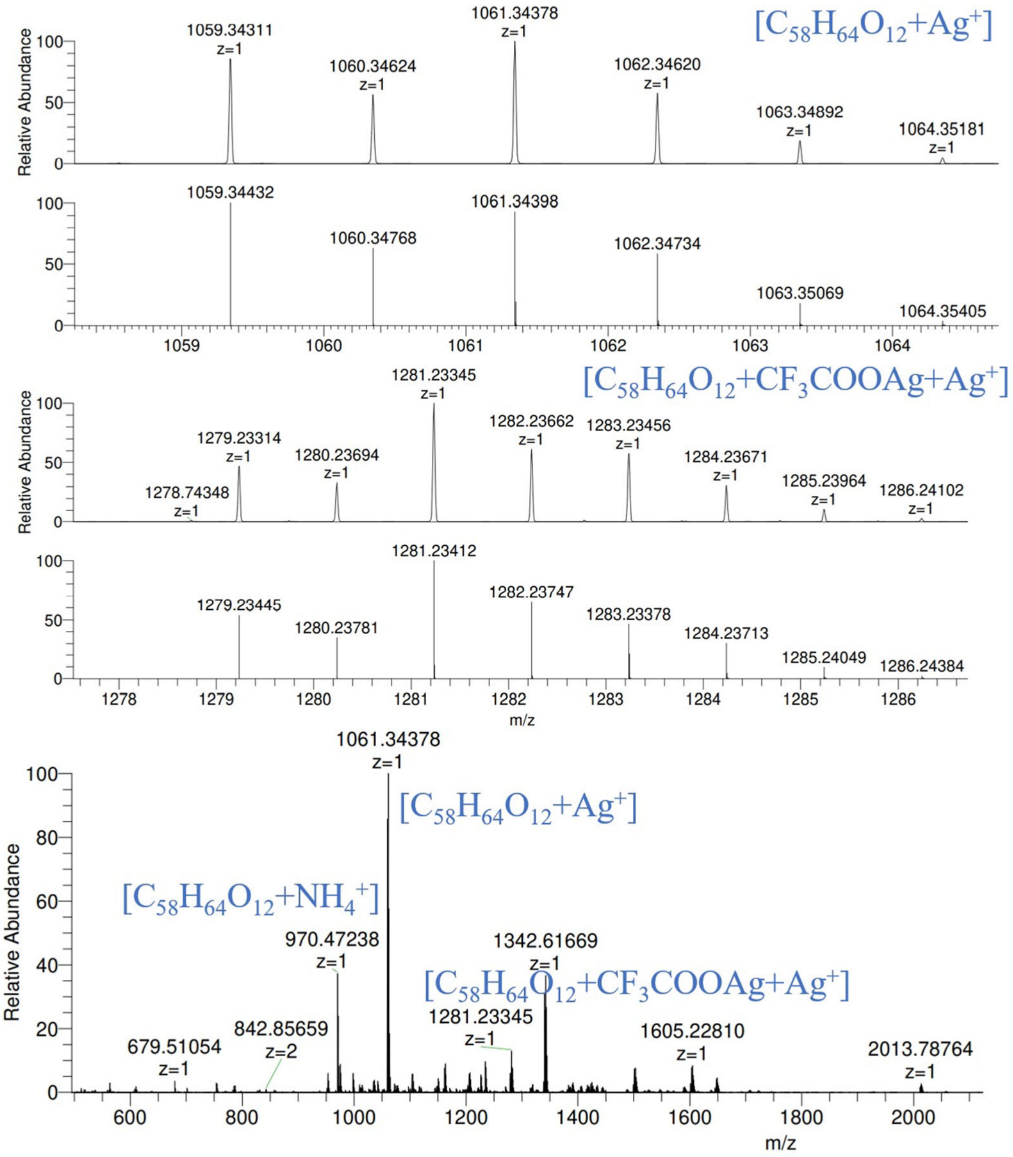

Figure S26. Electrospray ionization mass spectrum of **P6Q2** and CF_3COOAg (molar ratio: 1/1). Assignment of main peaks: *m*/*z* 970.47 [**P6Q2** + NH_4]$^+$; *m*/*z* 1061.34 [**P6Q2** + Ag]$^+$; 1281.23 [**P6Q2** + CF_3COOAg + Ag]$^+$.

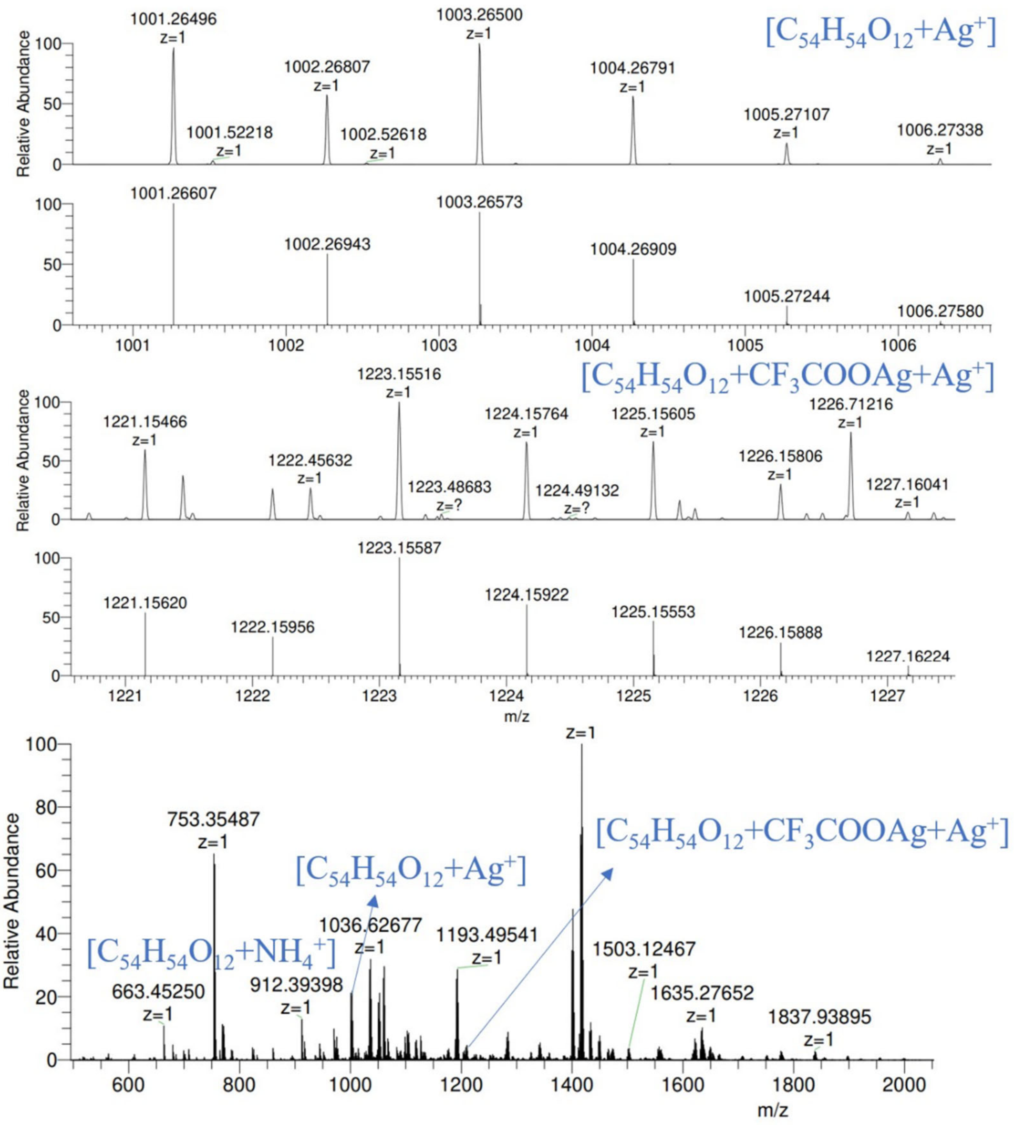

Figure S27. Electrospray ionization mass spectrum of **P6Q3** and CF_3COOAg (molar ratio: 1/1). Assignment of main peaks: *m*/*z* 912.39 [**P6Q3** + NH_4]$^+$; *m*/*z* 1003.26 [**P6Q3** + Ag]$^+$; 1223.15 [**P6Q3** + CF_3COOAg + Ag]$^+$.

As shown in **Figure S22-S27**, peaks at *m*/*z* 1079.13, 1219.29, 1397.39, 1339.31, 1281.23 and 1223.15 were observed, corresponding to [**MeP5** + CF_3COOAg + Ag]$^+$, [**EtP5** + CF_3COOAg + Ag]$^+$, [**EtP6** + CF_3COOAg + Ag]$^+$, [**P6Q1** + CF_3COOAg + Ag]$^+$, [**P6Q2** + CF_3COOAg + Ag]$^+$ and [**P6Q3** + CF_3COOAg + Ag]$^+$,respectively. The results obtained from ESI-MS spectra further proved that pillararenes formed host−guest complexes with CF_3COOAg. The samples used in ESI-MS are mixtures of host and guest with the ratio of 1:1, and the concentrations of both host and guest are 1 mmol/L .

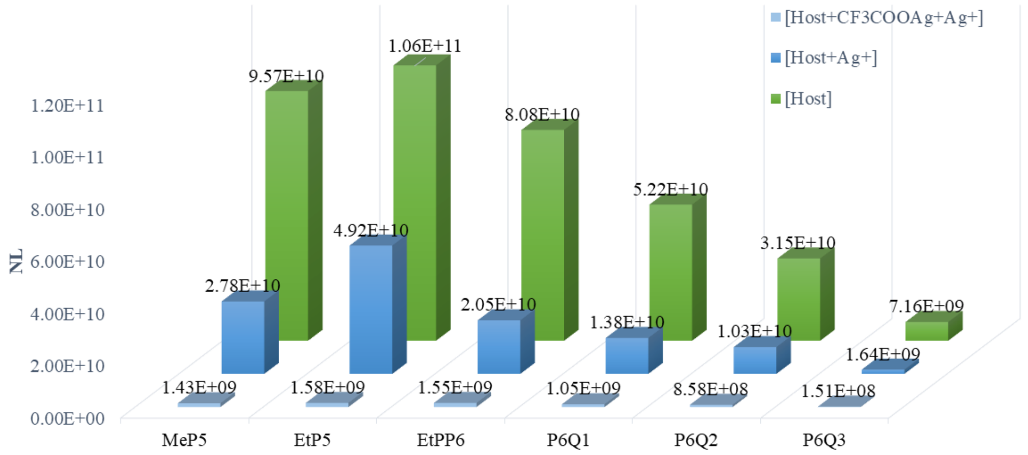

Figure S28. Histogram of NL values in ESS-MS experiments for single-host samples: [**MeP5** + 1 equiv. CF_3COOAg], [**EtP5** + 1 equiv. CF_3COOAg], [**EtP6** + 1 equiv. CF_3COOAg], [**P6Q1** + 1 equiv. CF_3COOAg], [**P6Q2** + 1 equiv. CF_3COOAg] and [**P6Q3** + 1 equiv. CF_3COOAg].

4. Optimized geometry structures of Pillararenes-Ag^+ complexes

All DFT calculations were carried out with the Gaussian 16 suite of computational programs.[10] The geometries of all stationary points were optimized using the wB97XD hybrid functional using the def2TZVP basis set.[11] All geometry optimizations were performed without symmetry constrains. Vibrational frequencies were analytically computed at the same level of theory to obtain the Gibbs free energies and to confirm whether the structures are minima.

The theoretical calculations were performed using the Density Functional Theory by wB97XD/Def2TZV level of theory (in vacuum). The binding energy of CF_3COOAg with pillararenes was defined as:

$$E_{binding} = E_{(P+n*CF3COOAg)} - E_P - n * E_{CF3COOAg}$$

where $E_{(P+CF3COOAg)}$ refers to the free energy of stable complex of pillararenes and one or two CF_3COOAg, E_P is the free energy of pillararenes, and $E_{CF3COOAg}$ is the free energy of CF_3COOAg.

Host	$E_{binding}$ with 1 CF_3COOAg (kcal/mol)	$E_{binding}$ with 2 CF_3COOAg (kcal/mol)
MeP5	13.9	-36.3
EtP5	15.3	-42.8
EtP6	13.4	-36.5
P6Q1	20.4	-31.4
P6Q2	21.2	-27.7
P6Q3	20.3	-23.3

Table S1. Calculated binding energy of **MeP5**, **EtP5**, **EtP6**, **P6Q1**, **P6Q2** and **P6Q3** with one CF_3COOAg and two CF_3COOAg.

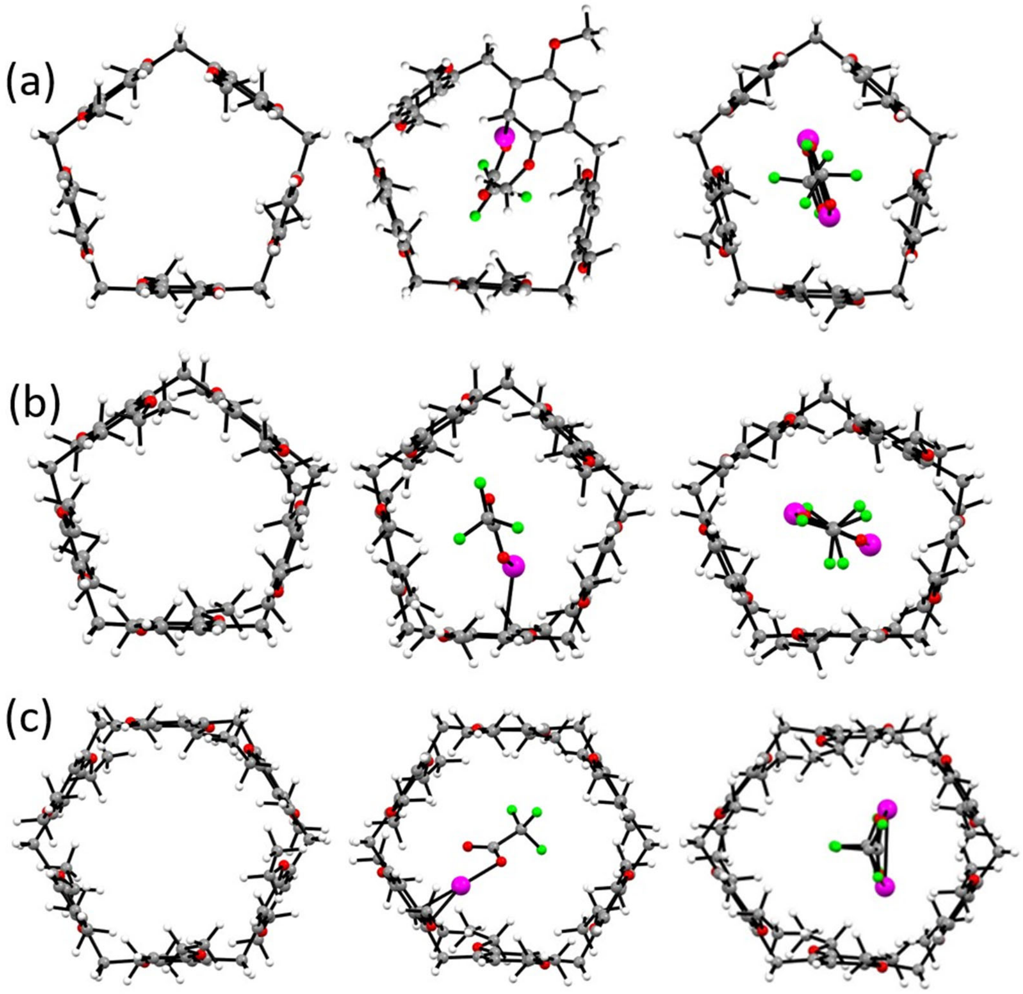
(a)
(b)
(c)

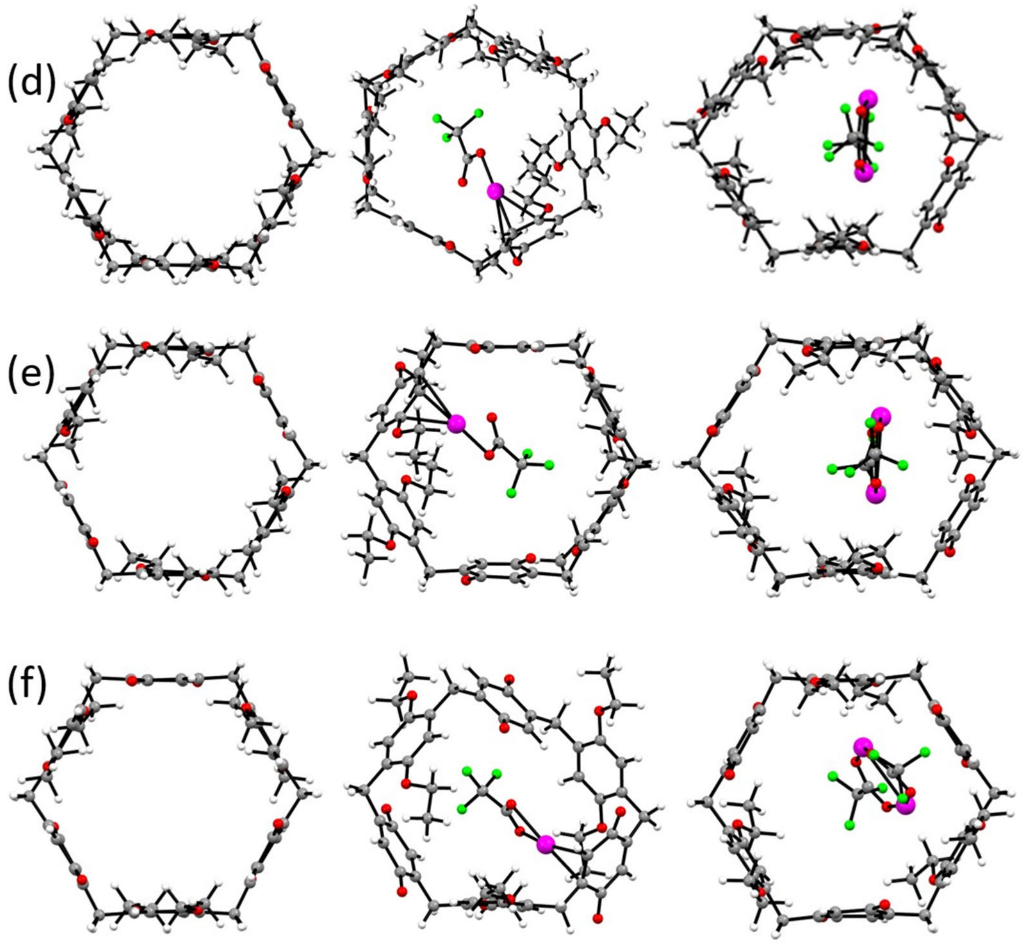

Figure S29. Top view of optimized structures of host itself, complexes with one CF_3COOAg and complexes with two CF_3COOAg in cavities of (a) **MeP5**, (b) **EtP5**, (c) **EtP6**, (d) **P6Q1**, (e) **P6Q2** and (f) **P6Q3**.

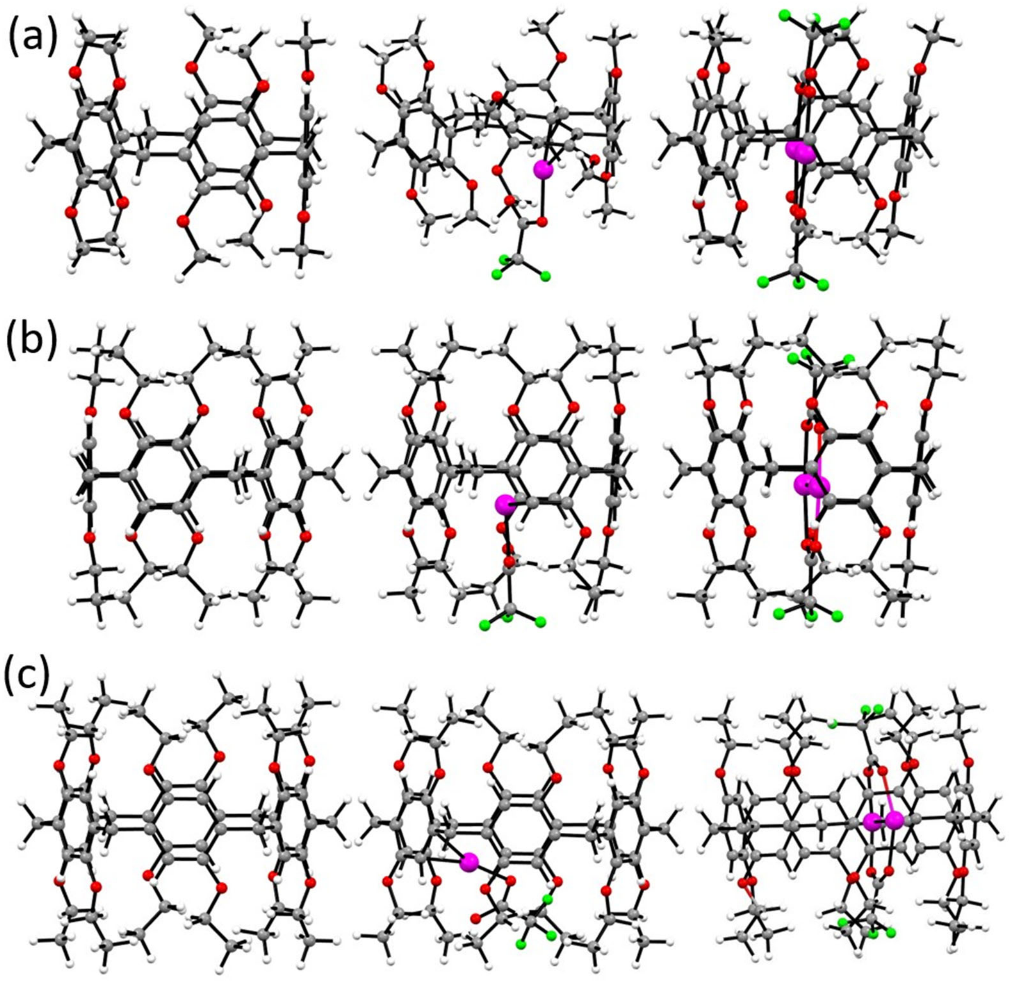
(a)
(b)
(c)

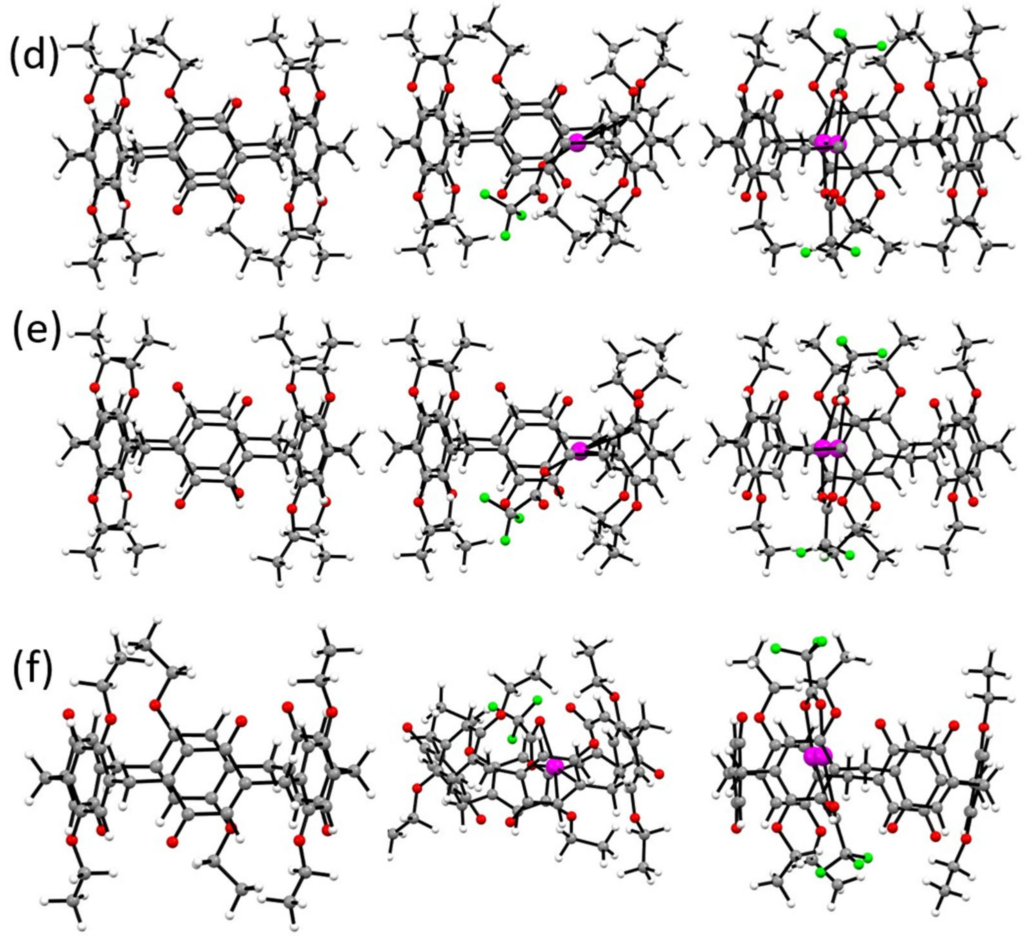

Figure S30. Side view of optimized structures of host itself, complexes with one CF_3COOAg and complexes with two CF_3COOAg in cavities of (a) **MeP5**, (b) **EtP5**, (c) **EtP6**, (d) **P6Q1**, (e) **P6Q2** and (f) **P6Q3**.

5. Selective binding of terminal alkynes by silver-loaded cavities

We did researches on the binding between 1-heptyne, 1-hexene, 3-hexene, 1-hexyne and 3-hexyne with silver-loaded cavity of **EtP6**/**P6Q1**. When silver ions were added to 1-heptyne, peaks of protons close to the alkyne group on ^{1}H NMR undergo a very significant chemical shift (**Figure S31** (c) & (d)). However, there is no binding between alkyne and EtP6 (**Figure S31** (c) & (e)). When we mixed alkyne, **EtP6** and CF_3COOAg in proportion, the proton peaks on **EtP6** have little chemical shifts, partial proton peaks on the alkyne have very obvious chemical shifts (**Figure S31** (f)). This is also the case in ^{13}C spectra (**Figure S32**). At the same time, we also characterized the DOSY spectra (**Figure S33** (c)) and found that the proton peaks of alkyne bound to silver ions and the proton peaks of **EtP6** have the same diffusion coefficient which means both 1-heptyne which complexed with silver was in the cavity of **EtP6**. The combination of these results shows that alkyne can bind in the cavity of **EtP6** with the exist of silver ions. The same situation is true for 1-hexyne and **P6Q1**(**Figure S33&S35**). 1-heptyne that bound to silver ions were accounted for 74% and 68% for **EtP6** and **P6Q1**, respectively (**Figure S33** (a)&(b)). Alkenes and internal alkynes can theoretically also complex with silver ions, but our experimental results show that there is no significant chemical shift in the proton peaks on NMR spectra. We also characterized the DOSY spectra (**Figure S36-38**) and found that the proton peaks of 1-hexene, 3-hexene, 3-hexyne and the proton peaks of **EtP6** have different diffusion coefficient which means either of them were loaded into the cavity of **EtP6**. Therefore, it can be concluded that only terminal alkyne could bind to silver-loaded **EtP6**.

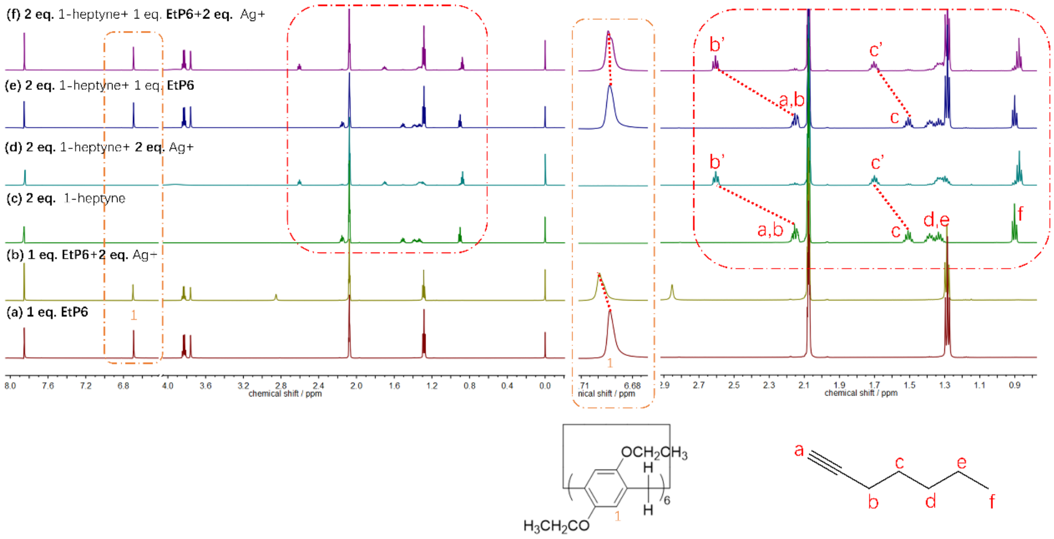

Figure S31. ^{1}H NMR spectra (400 MHz, $CDCl_3$: acetone-d_6 = 1:2, v/v, room temperature) of (a) 1 equiv. **EtP6**, (b) 1 equiv. **EtP6**+2 equiv. Ag^+, (c) 2 equiv. 1-heptyne, (d) 2 equiv. 1-heptyne + 2 equiv. Ag^+, (e) 2 equiv. 1-heptyne + 1 equiv. **EtP6** and (f) 2 equiv. 1-heptyne + 1 equiv. **EtP6** + 2 equiv. Ag^+.

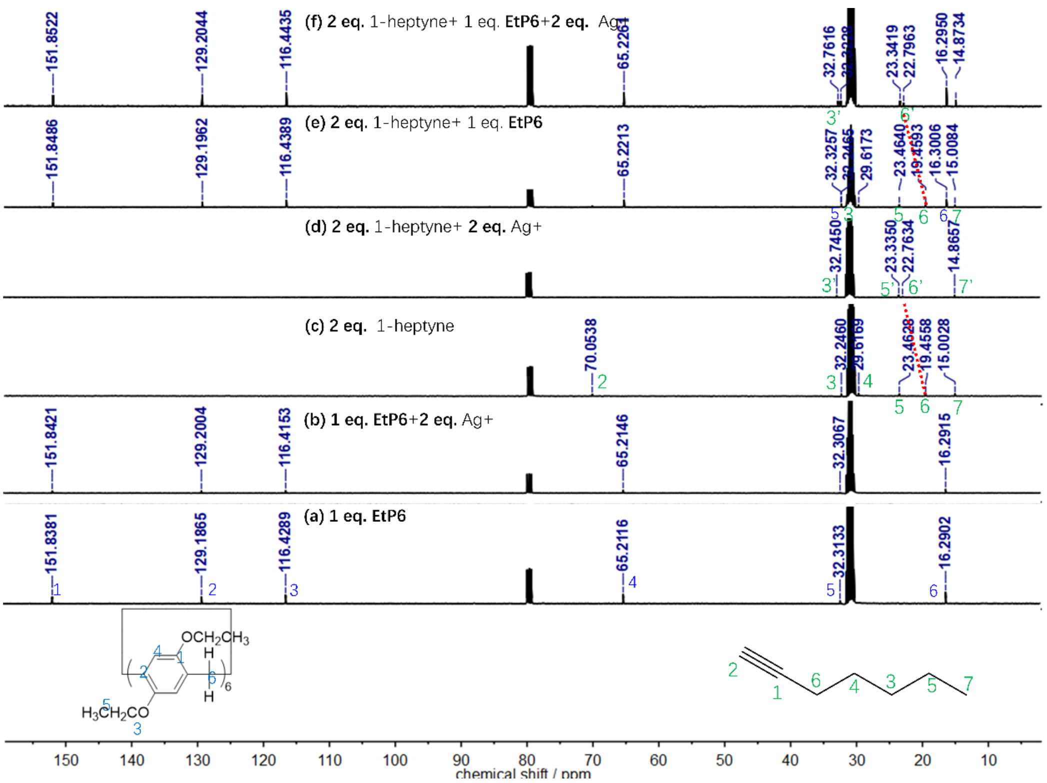

Figure S32. ^{13}C NMR spectra (101 MHz, $CDCl_3$: acetone-d_6 = 1:2, v/v, room temperature) of (a) 1 equiv. **EtP6**, (b) 1 equiv. **EtP6**+2 equiv. Ag^+, (c) 2 equiv. 1-heptyne, (d) 2 equiv. 1-heptyne + 2 equiv. Ag^+, (e) 2 equiv. 1-heptyne + 1 equiv. **EtP6** and (f) 2 equiv. 1-heptyne + 1 equiv. **EtP6** + 2 equiv. Ag^+.

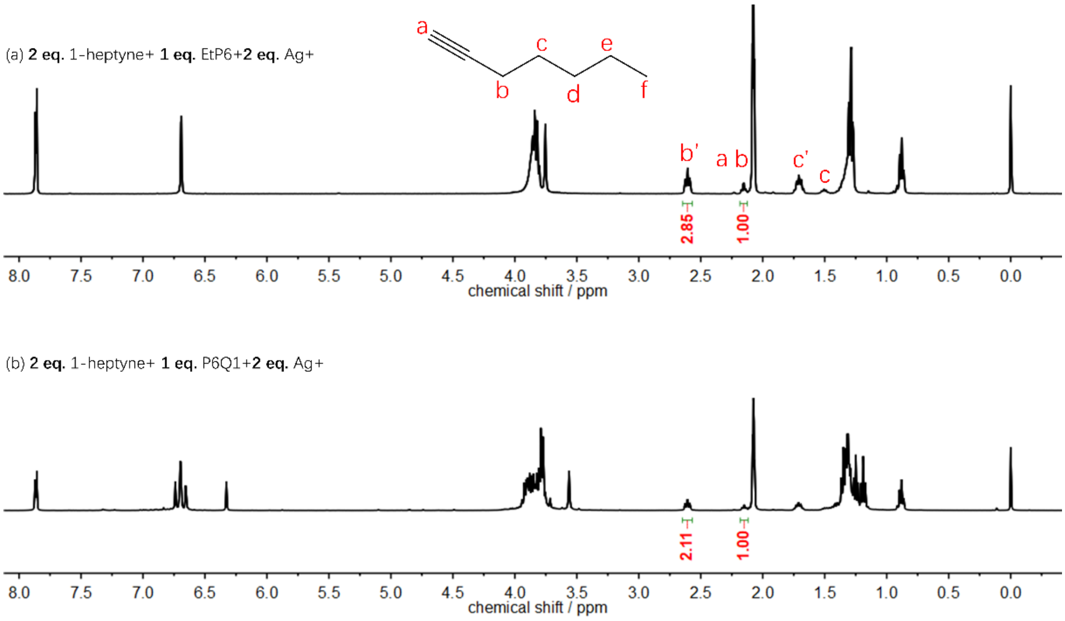

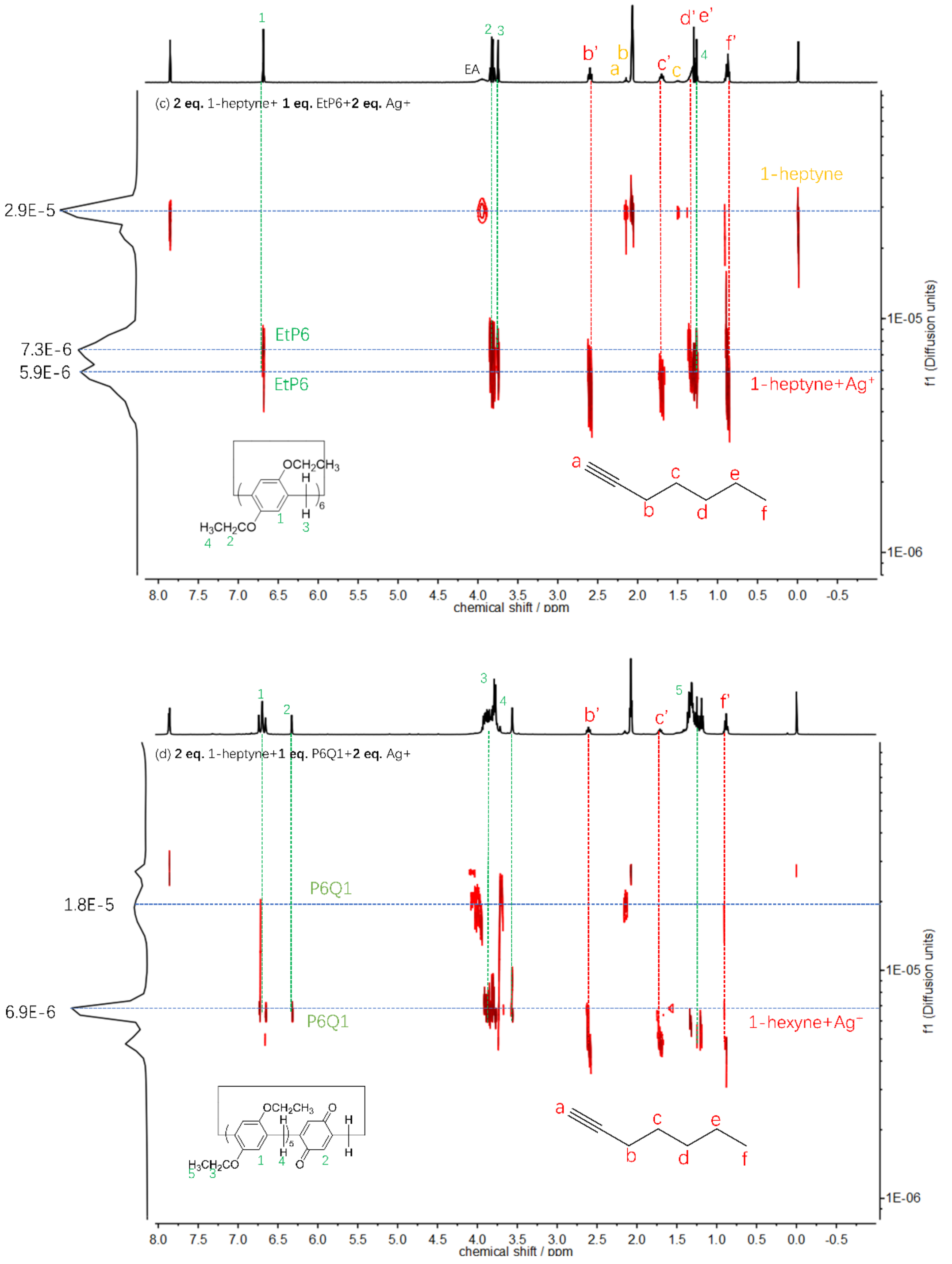

Figure S33. ¹H NMR spectra (400 MHz, $CDCl_3$: acetone-d_6 = 1:2, v/v, room temperature) of (a) 2 equiv. 1-heptyne + 1 equiv. **EtP6** + 2 equiv. Ag^+ and (b) 2 equiv. 1-heptyne + 1 equiv. **P6Q1** + 2 equiv. Ag^+; 2D DOSY NMR spectra ($CDCl_3$: acetone-d_6 = 1:2, v/v, room temperature) of (c) 2 equiv. 1-heptyne + 1 equiv. **EtP6** + 2 equiv. Ag^+ and (d) 2 equiv. 1-heptyne + 1 equiv. **EtP6** + 2 equiv. Ag^+.

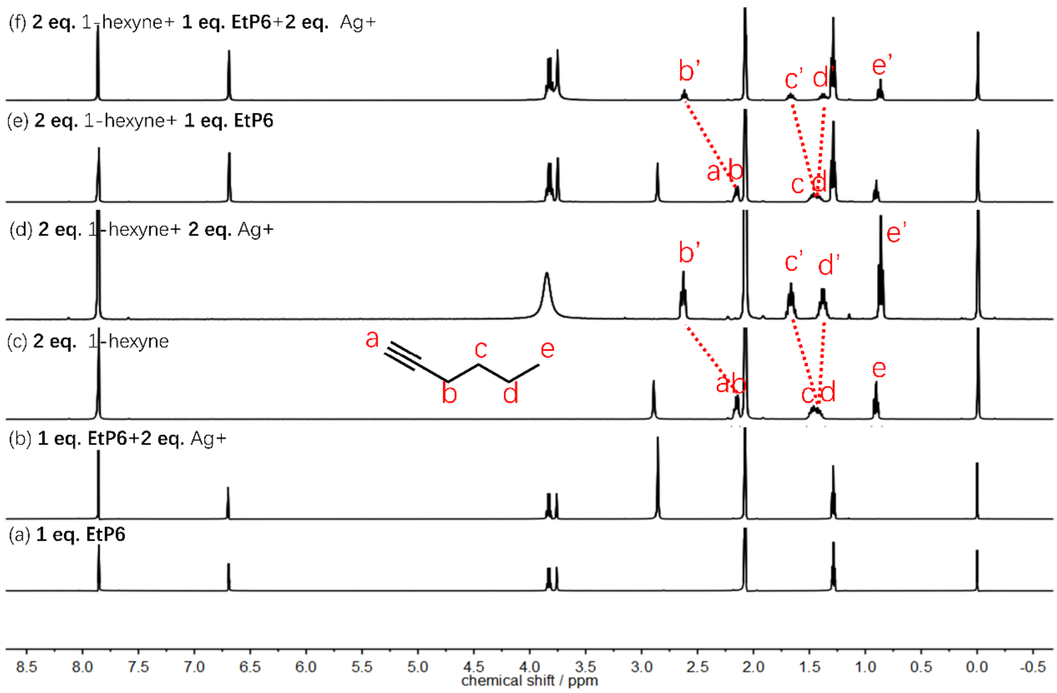

Figure S34. ^{1}H NMR spectra (400 MHz, $CDCl_3$: acetone-d_6 = 1:2, v/v, room temperature) of (a) 1 equiv. **EtP6**, (b) 1 equiv. **EtP6**+2 equiv. Ag^+, (c) 2 equiv. 1-hexyne, (d) 2 equiv. 1-hexyne + 2 equiv. Ag^+, (e) 2 equiv. 1-hexyne + 1 equiv. **EtP6** and (f) 2 equiv. 1-hexyne + 1 equiv. **EtP6** + 2 equiv. Ag^+.

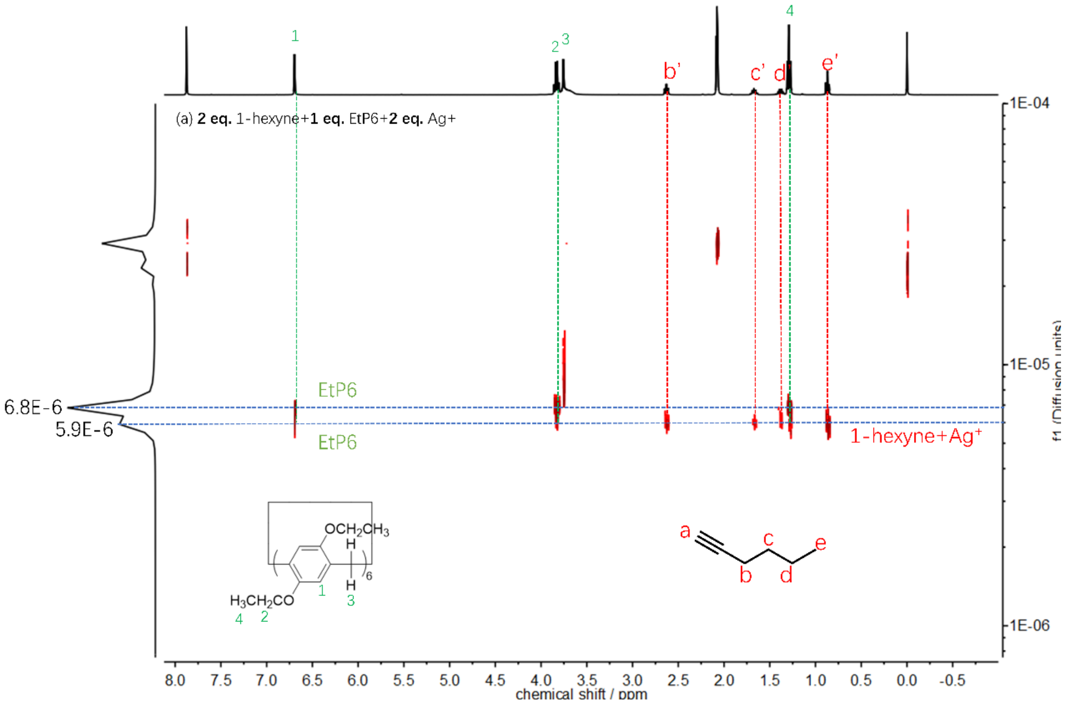

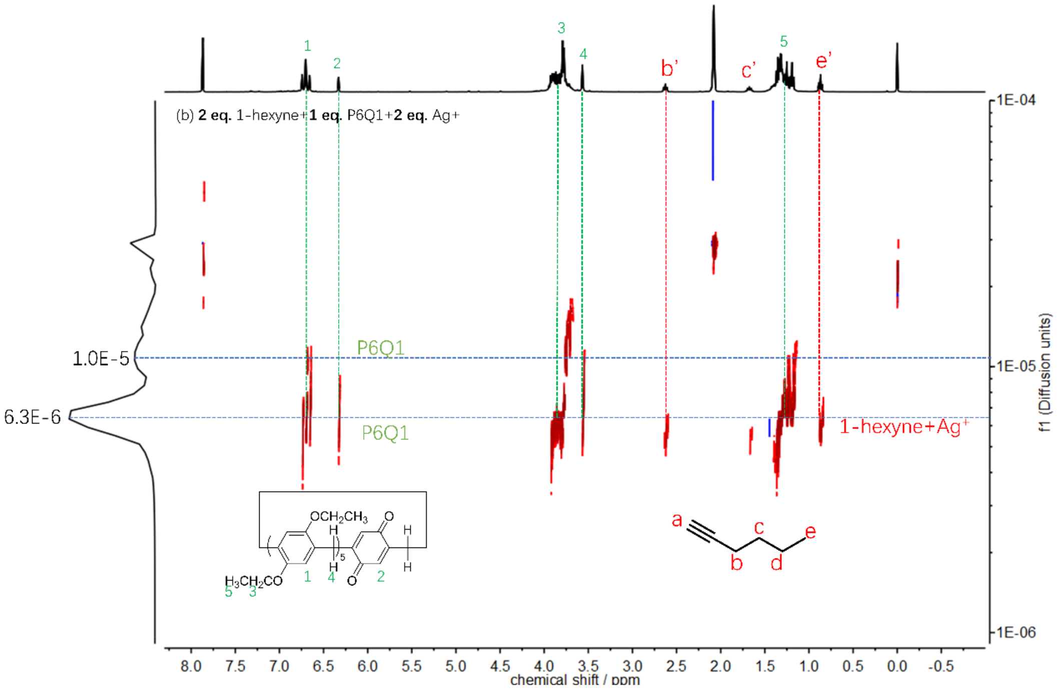

Figure S35. 2D DOSY NMR spectra ($CDCl_3$: acetone-d_6 = 1:2, v/v, room temperature) of (a) 2 equiv. 1-hexyne + 1 equiv. **EtP6** + 2 equiv. Ag$^+$ and (b) 2 equiv. 1-hexyne + 1 equiv. **P6Q1** + 2 equiv. Ag$^+$.

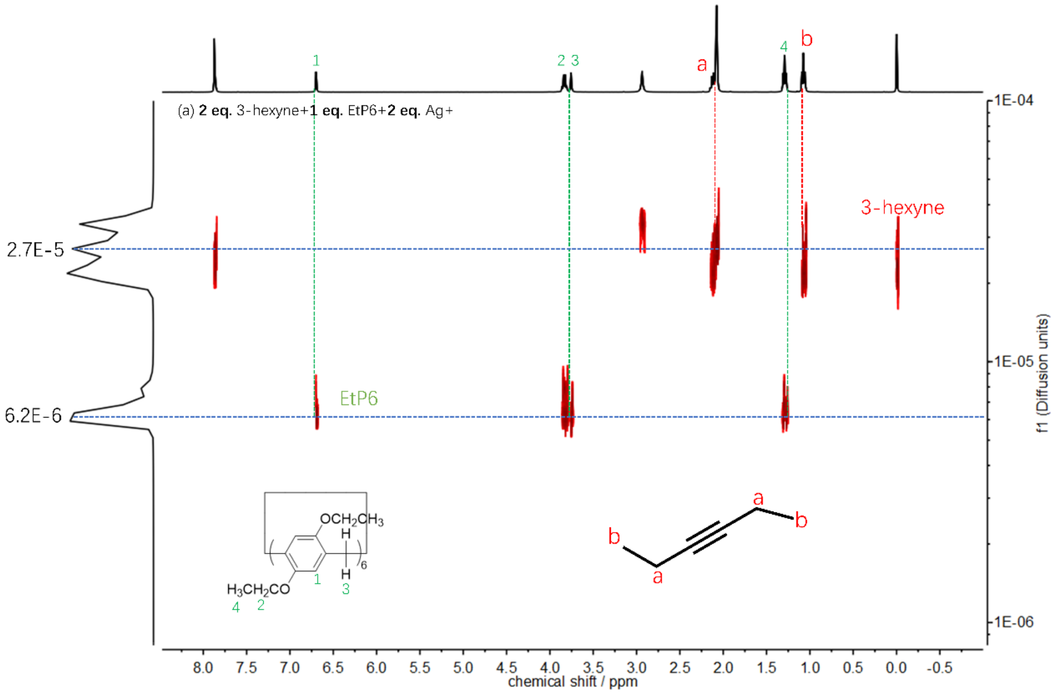

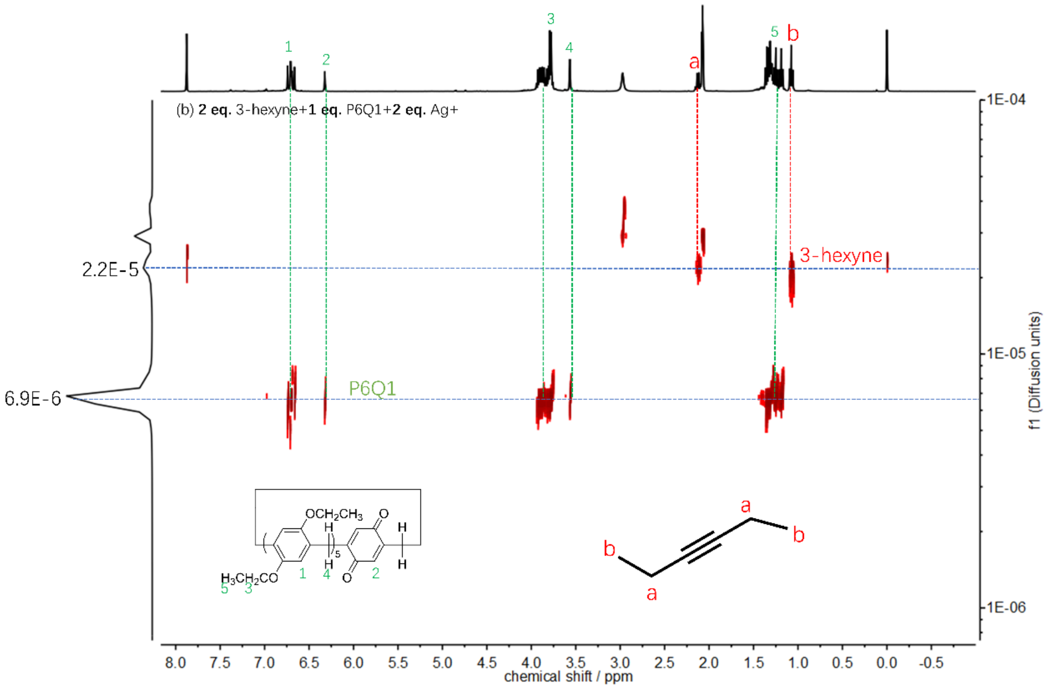

Figure S36. 2D DOSY NMR spectra ($CDCl_3$: acetone-d_6 = 1:2, v/v, room temperature) of (a) 2 equiv. 3-hexyne + 1 equiv. **EtP6** + 2 equiv. Ag^+ and (b) 2 equiv. 3-hexyne + 1 equiv. **P6Q1** + 2 equiv. Ag^+.

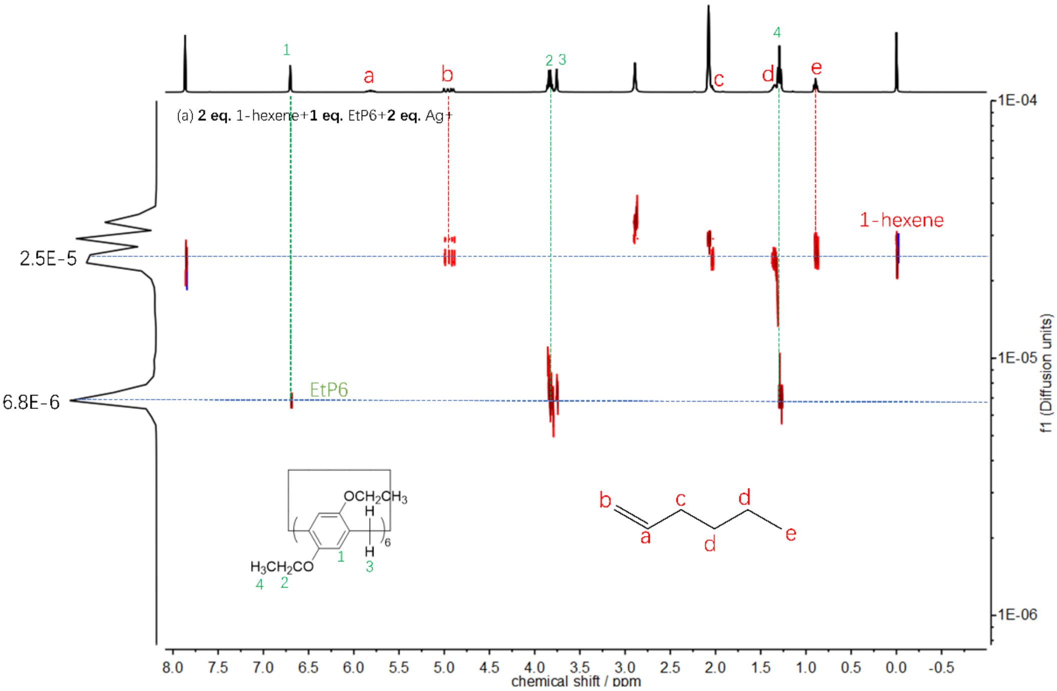

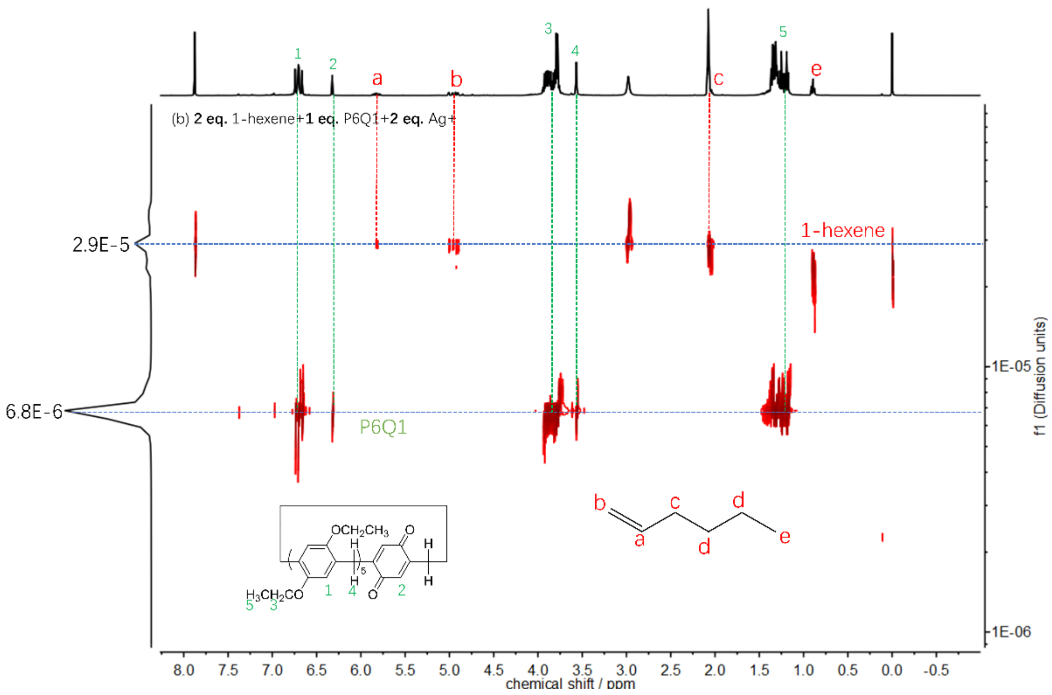

Figure S37. 2D DOSY NMR spectra ($CDCl_3$: acetone-d_6 = 1:2, v/v, room temperature) of (a) 2 equiv. 1-hexene + 1 equiv. **EtP6** + 2 equiv. Ag^+ and (b) 2 equiv. 1-hexene + 1 equiv. **P6Q1** + 2 equiv. Ag^+.

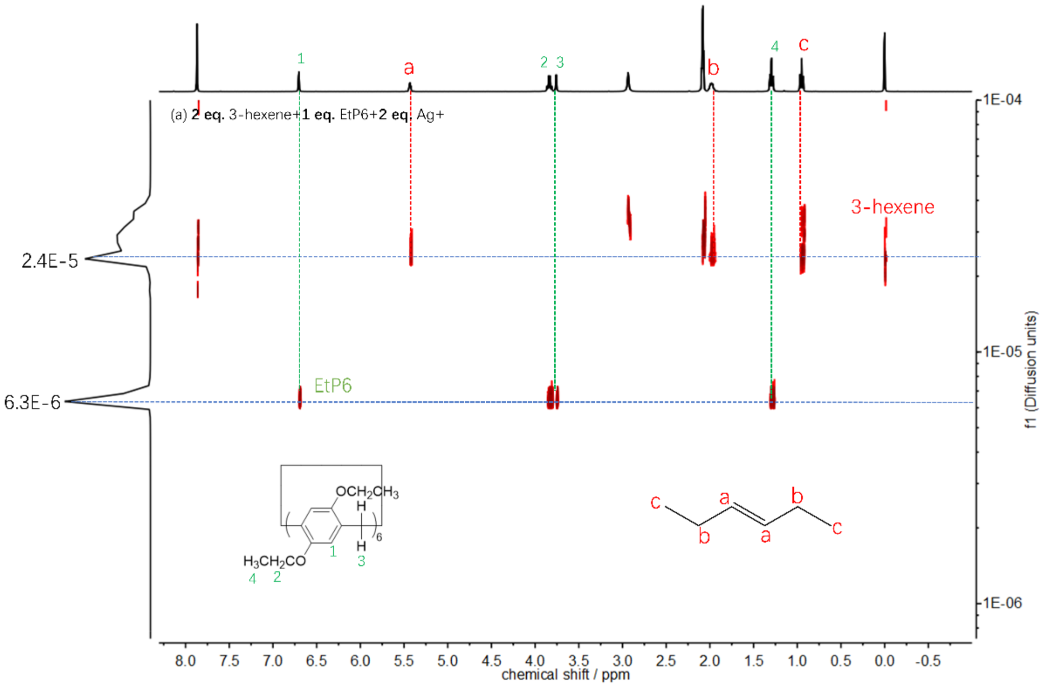

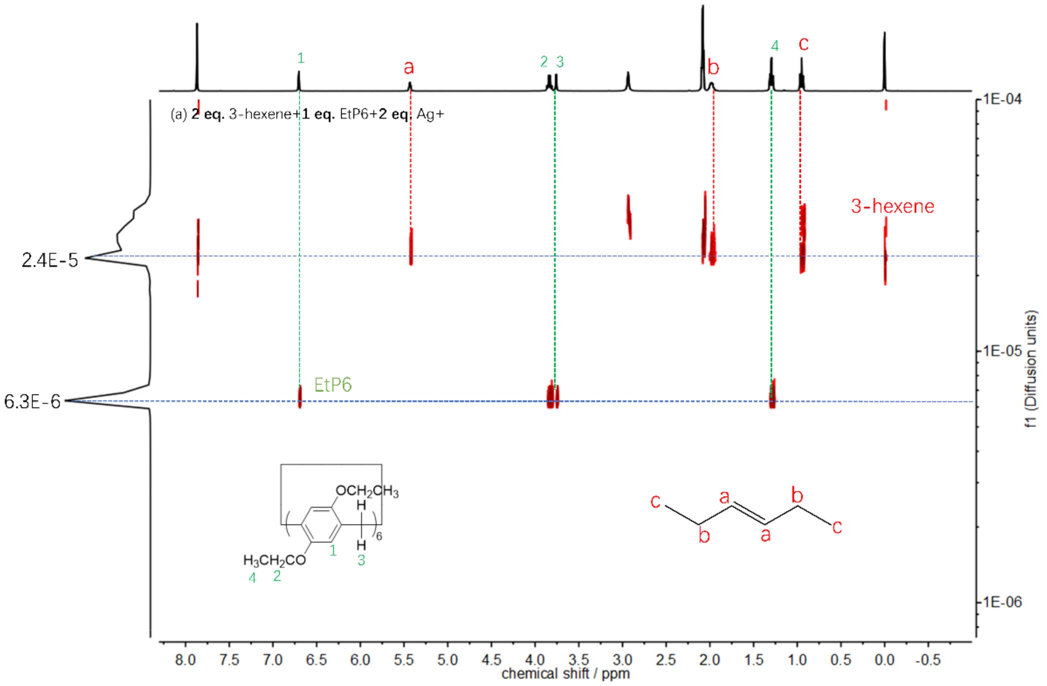

Figure S38. 2D DOSY NMR spectra ($CDCl_3$: acetone-d_6 = 1:2, v/v, room temperature) of (a) 2 equiv. 3-hexene + 1 equiv. **EtP6** + 2 equiv. Ag^+ and (b) 2 equiv. 3-hexene + 1 equiv. **P6Q1** + 2 equiv. Ag^+.

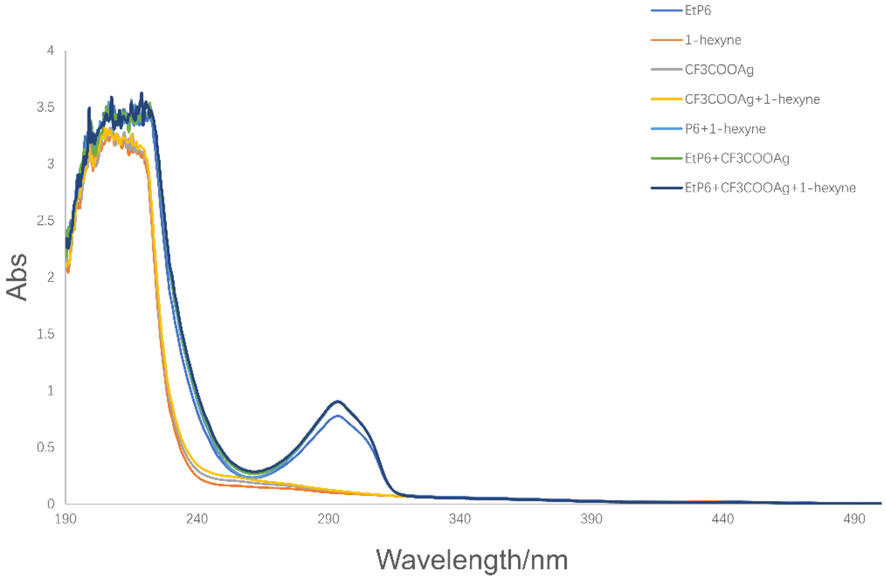

Figure S39. UV–visible spectra of **EtP6**, 1-hexyne, CF_3COOAg, CF_3COOAg+1-hexyne, **EtP6**+1-hexyne, **EtP6**+CF_3COOAg and **EtP6**+CF_3COOAg+1-hexyne.

6. Cartesian coordinates and energies of optimized structures

----

$AgOCOCF_3$

Sum of electronic and zero-point Energies= -673.292386

Sum of electronic and thermal Energies= -673.284456

Sum of electronic and thermal Enthalpies= -673.283511

Sum of electronic and thermal Free Energies= -673.330457

9	2.673302000	-0.763723000	-0.988700000
9	2.642663000	-0.500212000	1.157184000
9	2.732945000	1.215418000	-0.144272000
8	0.088900000	1.134755000	-0.010604000
8	0.097304000	-1.087489000	-0.010408000
6	0.657315000	0.027451000	-0.013665000
6	2.207306000	0.004112000	-0.002174000
47	-1.938671000	-0.002784000	0.000962000

MeP5

Sum of electronic and zero-point Energies= -2496.309013

Sum of electronic and thermal Energies= -2496.256006

Sum of electronic and thermal Enthalpies= -2496.255061

Sum of electronic and thermal Free Energies= -2496.399213

6	4.890499000	0.836266000	-0.002489000
1	5.522291000	0.957286000	0.876717000
1	5.524096000	0.931922000	-0.883513000
6	3.843546000	1.925726000	-0.011682000
6	3.325456000	2.421157000	-1.206113000
6	2.340164000	3.398213000	-1.182365000
1	1.923002000	3.789407000	-2.098990000
6	1.830667000	3.888718000	0.011661000
6	2.340507000	3.384810000	1.206112000
6	3.342023000	2.424367000	1.182353000
1	3.744477000	2.018229000	2.099121000
6	0.715633000	4.908415000	0.002056000
1	0.795278000	5.544358000	0.882946000
1	0.821293000	5.542878000	-0.877205000
6	-0.644266000	4.249922000	-0.009440000
6	-1.273752000	3.924158000	1.183649000
1	-0.763354000	4.178842000	2.101221000
6	-2.497036000	3.269115000	1.205629000
6	-3.133693000	2.943023000	0.010358000
6	-2.509601000	3.278954000	-1.182748000
1	-3.010557000	3.005519000	-2.100136000
6	-1.275824000	3.914017000	-1.204791000
6	-4.447933000	2.197646000	-0.000930000
1	-5.017116000	2.493510000	-0.881415000
1	-5.029738000	2.470507000	0.878750000
6	-4.241524000	0.700905000	-0.010925000
6	-4.113453000	-0.004097000	-1.205498000
6	-3.890746000	-1.373713000	-1.182001000
1	-3.782543000	-1.935038000	-2.098839000
6	-3.767389000	-2.070372000	0.011891000
6	-3.884169000	-1.363418000	1.206499000
6	-4.129459000	0.002358000	1.182937000
1	-4.216553000	0.567373000	2.099734000
6	-3.464421000	-3.550609000	0.001877000

1	-3.922235000	-4.001270000	-0.877935000	1	2.222572000	2.124697000	-3.641032000
1	-3.903079000	-4.019000000	0.882203000	1	3.459082000	3.404410000	-3.737398000
6	-1.977128000	-3.816762000	-0.009355000	1	3.815115000	1.778038000	-4.359552000
6	-1.276566000	-3.923417000	1.183852000	8	2.990008000	-3.063850000	-2.360199000
1	-1.839727000	-3.829893000	2.101116000	6	3.236286000	-2.416434000	-3.580624000
6	0.098157000	-4.112244000	1.205965000	1	2.706659000	-1.459600000	-3.642462000
6	0.805037000	-4.222358000	0.010654000	1	4.305589000	-2.240785000	-3.738588000
6	0.102785000	-4.127118000	-1.182511000	1	2.868401000	-3.081975000	-4.359586000
1	0.668784000	-4.199847000	-2.099941000	8	-1.991214000	-3.794934000	-2.357775000
6	-1.268606000	-3.915517000	-1.204616000	6	-1.299944000	-3.833742000	-3.578387000
6	2.306469000	-4.391877000	-0.000610000	1	-0.552390000	-3.035704000	-3.642961000
1	2.616703000	-4.953904000	0.879549000	1	-0.803978000	-4.797665000	-3.733586000
1	2.592895000	-4.966640000	-0.880617000	1	-2.046696000	-3.690758000	-4.357465000
6	3.019520000	-3.059744000	-0.011811000	8	-4.219500000	0.719840000	-2.359271000
6	3.340239000	-2.427972000	1.181477000	6	-4.038522000	0.050485000	-3.579319000
1	3.079070000	-2.935630000	2.098752000	1	-3.047675000	-0.412375000	-3.641294000
6	3.945016000	-1.179089000	1.203755000	1	-4.800479000	-0.720169000	-3.736544000
6	4.265536000	-0.539364000	0.008552000	1	-4.132365000	0.804677000	-4.358696000
6	3.955210000	-1.176230000	-1.184771000	8	-3.746782000	-2.081845000	2.360477000
1	4.197302000	-0.659225000	-2.102083000	6	-3.802607000	-1.390800000	3.580588000
6	3.330005000	-2.415015000	-1.207018000	1	-3.022285000	-0.624775000	3.645214000
8	-0.620968000	4.242498000	-2.357951000	1	-4.777946000	-0.917358000	3.734746000
6	-1.202393000	3.867359000	-3.578810000	1	-3.642815000	-2.133466000	4.360305000
1	-1.336177000	2.782141000	-3.644095000	8	0.825535000	-4.201205000	2.359004000
1	-2.171037000	4.354078000	-3.733824000	6	0.153345000	-4.036128000	3.579755000
1	-0.514739000	4.192306000	-4.357648000	1	-0.333596000	-3.056894000	3.641678000
8	3.836905000	1.898306000	-2.360078000	1	-0.598264000	-4.816619000	3.738253000
6	3.296225000	2.333007000	-3.579929000	1	0.910496000	-4.111059000	4.358318000

8	4.257212000	-0.516367000	2.356886000
6	3.896387000	-1.106775000	3.577730000
1	2.814762000	-1.267090000	3.642978000
1	4.406694000	-2.063195000	3.732814000
1	4.204321000	-0.411334000	4.356566000
8	1.806594000	3.884403000	2.360163000
6	2.257599000	3.357327000	3.580016000
1	2.076948000	2.278826000	3.643692000
1	3.324794000	3.547931000	3.734923000
1	1.691175000	3.863332000	4.359871000
8	-3.137586000	2.913258000	2.358711000
6	-2.497089000	3.176353000	3.579298000
1	-1.527181000	2.671145000	3.641752000
1	-2.348600000	4.249835000	3.736755000
1	-3.153435000	2.792158000	4.358180000

EtP5

Sum of electronic and zero-point Energies= -2889.246819

Sum of electronic and thermal Energies= -2889.180338

Sum of electronic and thermal Enthalpies= -2889.179394

Sum of electronic and thermal Free Energies= -2889.349321

8	-2.198668000	3.675576000	2.352889000
8	-4.019111000	1.480361000	-2.358248000
8	2.817871000	3.228851000	2.352945000
8	0.166286000	4.278052000	-2.358303000
8	3.939744000	-1.678591000	2.358787000
8	4.122748000	1.160812000	-2.356417000
8	-0.383827000	-4.262148000	2.359028000
8	2.383159000	-3.563175000	-2.350679000
8	-4.178691000	-0.953415000	2.354296000
8	-2.649025000	-3.365328000	-2.354319000
6	-4.951059000	0.318194000	-0.002461000
1	-5.591946000	0.347288000	-0.882786000
1	-5.591046000	0.371416000	0.877409000
6	-4.023609000	1.510944000	-0.011351000
6	-3.574713000	2.059417000	1.181793000
1	-3.931694000	1.614974000	2.099807000
6	-2.678825000	3.119359000	1.201873000
6	-2.228298000	3.673406000	0.005926000
6	-2.683946000	3.130575000	-1.187214000
1	-2.311962000	3.562687000	-2.105183000
6	-3.561181000	2.055095000	-1.207260000
6	-2.477195000	3.035999000	3.576312000
1	-2.142684000	1.991939000	3.532801000
1	-3.557229000	3.032807000	3.767714000
6	-1.752890000	3.786594000	4.669295000
1	-0.676413000	3.779800000	4.493138000
1	-1.947863000	3.320232000	5.636166000
1	-2.088370000	4.823188000	4.710761000
6	-3.439858000	1.869600000	-3.581483000
1	-2.352191000	1.732617000	-3.536128000
1	-3.634871000	2.931475000	-3.775112000
6	-4.043186000	1.018019000	-4.673947000
1	-3.839532000	-0.038655000	-4.495583000
1	-3.618872000	1.293459000	-5.640609000
1	-5.123642000	1.157743000	-4.717424000

6	-1.227237000	4.805066000	-0.002561000	6	4.090139000	0.428345000	-1.204333000
1	-1.397372000	5.424220000	-0.882505000	6	3.786986000	-0.996062000	3.581188000
1	-1.374819000	5.429576000	0.877654000	1	2.902886000	-0.347907000	3.535278000
6	0.193838000	4.292001000	-0.011317000	1	4.658649000	-0.358674000	3.773619000
6	0.854612000	4.035432000	1.181753000	6	3.640200000	-2.027717000	4.675146000
1	0.321777000	4.237631000	2.099864000	1	2.765914000	-2.655477000	4.498005000
6	2.139966000	3.511977000	1.201827000	1	3.521594000	-1.534558000	5.641111000
6	2.806007000	3.254336000	0.005938000	1	4.521010000	-2.668818000	4.719376000
6	2.148526000	3.519248000	-1.187124000	6	3.885899000	0.503086000	-3.579000000
1	2.674224000	3.299361000	-2.105276000	1	2.926528000	-0.027445000	-3.534315000
6	0.854344000	4.020450000	-1.207201000	1	4.669691000	-0.239965000	-3.770466000
6	2.123931000	3.297192000	3.576549000	6	3.872673000	1.545031000	-4.673009000
1	1.234947000	2.655484000	3.534288000	1	3.694208000	1.070927000	-5.639308000
1	1.786220000	4.323298000	3.766651000	1	4.827648000	2.069447000	-4.715555000
6	3.062474000	2.842976000	4.669913000	1	3.084596000	2.278393000	-4.497089000
1	3.389566000	1.817109000	4.495448000	6	3.818249000	-3.166645000	0.005318000
1	2.559001000	2.885508000	5.636893000	1	4.304057000	-3.584172000	0.886365000
1	3.944089000	3.483252000	4.709957000	1	4.319942000	-3.569010000	-0.873881000
6	4.191741000	2.652265000	-0.002884000	6	2.366707000	-3.586090000	-0.003732000
1	4.740961000	2.987052000	0.876241000	6	1.680018000	-3.763909000	1.189048000
1	4.727084000	3.004115000	-0.883896000	1	2.229068000	-3.612390000	2.107381000
6	4.143145000	1.142142000	-0.009401000	6	0.332286000	-4.095262000	1.208368000
6	4.102139000	0.436245000	1.184749000	6	-0.356781000	-4.280734000	0.012129000
1	4.128820000	1.006933000	2.102000000	6	0.332048000	-4.111286000	-1.180651000
6	4.001265000	-0.947919000	1.206743000	1	-0.221746000	-4.244146000	-2.098987000
6	3.963407000	-1.662701000	0.011775000	6	1.673808000	-3.756731000	-1.200028000
6	4.013740000	-0.957405000	-1.182337000	6	0.216240000	-3.906558000	3.582462000
1	3.968088000	-1.526810000	-2.099633000	1	0.558183000	-2.864942000	3.538302000

1	1.092374000	-4.537735000	3.775051000
6	-0.811373000	-4.088191000	4.675035000
1	-1.148382000	-5.124272000	4.716981000
1	-1.678746000	-3.450841000	4.498061000
1	-0.380213000	-3.824514000	5.641950000
6	1.687273000	-3.542176000	-3.574866000
1	0.887791000	-2.791859000	-3.533576000
1	1.221119000	-4.516608000	-3.765350000
6	2.677413000	-3.211858000	-4.667207000
1	2.173706000	-3.189403000	-5.634734000
1	3.469766000	-3.959805000	-4.706397000
1	3.133060000	-2.236352000	-4.492064000
6	-1.831761000	-4.608225000	0.002682000
1	-2.057187000	-5.209689000	-0.877118000
1	-2.079866000	-5.199768000	0.883102000
6	-2.680267000	-3.358094000	-0.007462000
6	-3.064301000	-2.761121000	1.185025000
1	-2.752196000	-3.237093000	2.103587000
6	-3.796566000	-1.582164000	1.203901000
6	-4.184023000	-0.983549000	0.007343000
6	-3.807652000	-1.585351000	-1.185209000
1	-4.103707000	-1.098992000	-2.103660000
6	-3.054973000	-2.751411000	-1.204068000
6	-3.656376000	-1.414099000	3.578367000
1	-2.560014000	-1.417118000	3.535421000
1	-3.985748000	-2.442510000	3.770667000
6	-4.148320000	-0.493060000	4.670312000
1	-5.237870000	-0.493411000	4.711219000
1	-3.810688000	0.528985000	4.493428000
1	-3.764980000	-0.821166000	5.637652000
6	-2.842463000	-2.696739000	-3.578629000
1	-2.376689000	-1.704227000	-3.536107000
1	-3.913014000	-2.555268000	-3.770957000
6	-2.219870000	-3.535455000	-4.670238000
1	-2.685538000	-4.520501000	-4.710608000
1	-1.151500000	-3.666573000	-4.493457000
1	-2.352983000	-3.049189000	-5.637802000
6	0.716226000	3.847929000	-3.581411000
1	1.666477000	4.360935000	-3.773649000
1	0.921157000	2.770961000	-3.536872000
6	-0.278791000	4.160345000	-4.674559000
1	-0.479451000	5.231210000	-4.716709000
1	0.115400000	3.843261000	-5.641213000
1	-1.221149000	3.640087000	-4.498113000

EtP6

Sum of electronic and zero-point Energies= -3467.091734

Sum of electronic and thermal Energies= -3467.011101

Sum of electronic and thermal Enthalpies= -3467.010157

Sum of electronic and thermal Free Energies= -3467.212980

6	-5.681658000	-1.285596000	0.004367000
6	-5.162648000	0.132262000	0.009042000
6	-4.940487000	0.804746000	-1.184348000
6	-4.511857000	2.124955000	-1.203265000
6	-4.330041000	2.815193000	-0.007092000
6	-4.525457000	2.134428000	1.186297000

6	-4.919584000	0.803482000	1.205175000	6	-1.766246000	4.664040000	1.200932000
6	-4.602879000	-2.341944000	-0.004208000	8	-4.277311000	2.823985000	-2.352314000
6	-4.109393000	-2.853868000	1.187500000	6	-4.310055000	2.134291000	-3.579951000
6	-3.154210000	-3.861067000	1.203214000	8	-5.119948000	0.093667000	2.354085000
6	-2.695658000	-4.404699000	0.005477000	6	-4.756058000	0.680452000	3.581737000
6	-3.168780000	-3.873951000	-1.186276000	8	-4.588944000	-2.289336000	-2.349512000
6	-4.098195000	-2.842892000	-1.202016000	6	-4.011921000	-2.662798000	-3.578924000
6	-1.727020000	-5.562896000	-0.002623000	8	-2.639389000	-4.392347000	2.350673000
6	2.467244000	-4.536948000	0.004344000	6	-2.965540000	-3.786834000	3.579838000
6	1.772069000	-4.678654000	-1.188314000	8	-0.309784000	-5.112248000	-2.354024000
6	0.414333000	-4.967379000	-1.205736000	6	0.302626000	-4.795151000	-3.582133000
6	-0.272810000	-5.156655000	-0.008897000	8	4.589421000	2.289299000	-2.348676000
6	0.416257000	-4.987677000	1.183763000	6	4.012672000	2.662796000	-3.578207000
6	1.766043000	-4.664038000	1.201200000	8	2.638866000	4.392264000	2.351112000
6	5.681687000	1.285575000	0.005411000	6	2.964841000	3.786828000	3.580360000
6	4.602898000	2.341913000	-0.003371000	8	0.310191000	5.112061000	-2.353959000
6	4.109155000	2.853816000	1.188241000	6	-0.302119000	4.795278000	-3.582196000
6	3.153960000	3.861008000	1.203765000	8	-2.482849000	4.485190000	2.349161000
6	2.695660000	4.404653000	0.005938000	6	-1.794294000	4.466806000	3.577720000
6	3.169030000	3.873919000	-1.185722000	8	2.482453000	-4.485193000	2.349548000
6	4.098452000	2.842863000	-1.201277000	6	1.793646000	-4.466649000	3.577963000
6	1.727018000	5.562845000	-0.002345000	6	3.954635000	-4.277657000	-0.001721000
6	-3.954638000	4.277640000	-0.002345000	6	5.162678000	-0.132285000	0.009988000
6	-2.467246000	4.536915000	0.003963000	6	4.940569000	-0.804694000	-1.183455000
6	-1.771869000	4.678554000	-1.188586000	6	4.511922000	-2.124894000	-1.202472000
6	-0.414130000	4.967270000	-1.205794000	6	4.330039000	-2.815209000	-0.006351000
6	0.272810000	5.156605000	-0.008845000	6	4.525420000	-2.134522000	1.187088000
6	-0.416456000	4.987676000	1.183705000	6	4.919565000	-0.803580000	1.206066000

8	4.277414000	-2.823840000	-2.351576000	1	-2.929170000	-2.489969000	-3.547746000
6	4.310428000	-2.134100000	-3.579177000	1	-4.174570000	-3.730930000	-3.767708000
8	5.119958000	-0.093870000	2.355039000	1	-2.708771000	-2.720902000	3.547471000
6	4.755700000	-0.680614000	3.582603000	1	-4.042918000	-3.865480000	3.769724000
1	-6.305859000	-1.436337000	0.884784000	1	0.695381000	-3.771579000	-3.546988000
1	-6.314430000	-1.419178000	-0.872703000	1	1.145636000	-5.470229000	-3.773483000
1	-5.116180000	0.262070000	-2.102466000	1	2.929907000	2.490011000	-3.547257000
1	-4.361085000	2.680834000	2.104320000	1	4.175404000	3.730923000	-3.766945000
1	-4.498902000	-2.440316000	2.107076000	1	2.708084000	2.720892000	3.548017000
1	-2.788035000	-4.295602000	-2.105849000	1	4.042190000	3.865492000	3.770398000
1	-1.926981000	-6.174501000	-0.882071000	1	-0.694942000	3.771722000	-3.547325000
1	-1.909036000	-6.182080000	0.875444000	1	-1.145069000	5.470453000	-3.773471000
1	2.328578000	-4.558003000	-2.107081000	1	-1.324533000	5.439854000	3.766161000
1	-0.137415000	-5.119940000	2.102610000	1	-0.998737000	3.712316000	3.545332000
1	6.314608000	1.419159000	-0.871550000	1	0.998145000	-3.712112000	3.545333000
1	6.305734000	1.436322000	0.885935000	1	1.323783000	-5.439650000	3.766397000
1	4.498477000	2.440254000	2.107892000	1	4.385328000	-4.756012000	-0.881003000
1	2.788463000	4.295567000	-2.105371000	1	4.398490000	-4.745660000	0.876619000
1	1.927120000	6.174439000	-0.881769000	1	5.116306000	-0.261963000	-2.101533000
1	1.908889000	6.182041000	0.875745000	1	4.361023000	-2.680986000	2.105073000
1	-4.385188000	4.756032000	-0.881675000	1	3.620100000	-1.282382000	-3.544302000
1	-4.398624000	4.745609000	0.875946000	1	5.316720000	-1.741130000	-3.768871000
1	-2.328230000	4.557862000	-2.107438000	1	5.362977000	-1.573801000	3.773473000
1	0.137070000	5.119966000	2.102633000	1	3.704643000	-0.992378000	3.546856000
1	-3.619653000	1.282638000	-3.544990000	6	-3.908628000	3.103925000	-4.666894000
1	-5.316278000	1.741234000	-3.769830000	1	-2.900401000	3.481551000	-4.490975000
1	-5.363522000	1.573539000	3.772480000	1	-3.927152000	2.607151000	-5.638045000
1	-3.705042000	0.992387000	3.546237000	1	-4.594003000	3.951454000	-4.698173000

6	0.739796000	4.931525000	-4.667847000
1	1.131379000	5.948730000	-4.699233000
1	1.570534000	4.247125000	-4.490281000
1	0.301612000	4.698635000	-5.639446000
6	3.909086000	-3.103655000	-4.666221000
1	3.927832000	-2.606855000	-5.637354000
1	4.594372000	-3.951259000	-4.697404000
1	2.900787000	-3.481173000	-4.490483000
6	-0.739164000	-4.931228000	-4.667927000
1	-0.300880000	-4.698129000	-5.639431000
1	-1.130692000	-5.948447000	-4.699557000
1	-1.569957000	-4.246905000	-4.490327000
6	-4.655206000	-1.831439000	-4.664186000
1	-4.478222000	-0.769252000	-4.489604000
1	-4.237422000	-2.096223000	-5.636604000
1	-5.731862000	-2.001936000	-4.691627000
6	4.656147000	1.831413000	-4.663339000
1	5.732812000	2.001879000	-4.690568000
1	4.479100000	0.769230000	-4.488787000
1	4.238564000	2.096201000	-5.635842000
6	4.972114000	0.346554000	4.669278000
1	4.705625000	-0.073180000	5.640393000
1	6.016649000	0.657921000	4.701296000
1	4.354330000	1.228102000	4.492433000
6	2.184979000	4.492114000	4.665335000
1	1.112424000	4.398885000	4.489285000
1	2.415066000	4.053997000	5.637632000
1	2.439092000	5.552121000	4.694083000
6	-2.793560000	4.144139000	4.664055000
1	-3.248305000	3.168043000	4.489423000
1	-2.298064000	4.125800000	5.635858000
1	-3.585230000	4.893439000	4.692904000
6	-4.972544000	-0.346776000	4.668341000
1	-6.017036000	-0.658308000	4.700135000
1	-4.354585000	-1.228223000	4.491599000
1	-4.706320000	0.072971000	5.639523000
6	-2.185846000	-4.492067000	4.664967000
1	-2.439979000	-5.552069000	4.693735000
1	-1.113265000	-4.398863000	4.489072000
1	-2.416076000	-4.053892000	5.637204000
6	2.792703000	-4.143955000	4.664482000
1	3.584341000	-4.893282000	4.693530000
1	3.247517000	-3.167886000	4.489894000
1	2.297012000	-4.125551000	5.636185000

P6Q1

Sum of electronic and zero-point Energies= -3308.746876

Sum of electronic and thermal Energies= -3308.672280

Sum of electronic and thermal Enthalpies= -3308.671335

Sum of electronic and thermal Free Energies= -3308.862368

6	-5.829547000	0.293621000	0.293384000
6	-5.012889000	1.562470000	0.249765000
6	-4.683217000	2.143296000	-0.966026000
6	-3.975589000	3.335721000	-1.029491000
6	-3.623394000	3.994328000	0.145896000
6	-3.923438000	3.399881000	1.363337000

6	-4.592483000	2.184861000	1.424462000
6	-5.007473000	-0.971861000	0.250378000
6	-4.570101000	-1.566614000	1.425462000
6	-3.855449000	-2.756626000	1.412176000
6	-3.595455000	-3.398957000	0.203413000
6	-4.008551000	-2.789490000	-0.972744000
6	-4.690665000	-1.580581000	-0.961619000
6	-2.905052000	-4.741238000	0.168499000
6	1.405219000	-4.663009000	-0.075195000
6	0.627956000	-4.648705000	-1.224685000
6	-0.758715000	-4.633166000	-1.161878000
6	-1.400141000	-4.664417000	0.074291000
6	-0.622901000	-4.650344000	1.223779000
6	0.763756000	-4.633409000	1.160987000
6	5.829687000	0.299497000	-0.292288000
6	5.011611000	1.567451000	-0.249034000
6	4.680694000	2.147846000	0.966620000
6	3.971709000	3.339480000	1.029803000
6	3.619404000	3.997797000	-0.145706000
6	3.920699000	3.403732000	-1.363031000
6	4.591077000	2.189436000	-1.423896000
6	2.950180000	5.351605000	-0.091652000
6	-2.955573000	5.348817000	0.091573000
6	-1.459274000	5.272222000	-0.013049000
6	-0.801826000	5.254724000	-1.173352000
6	0.669870000	5.219082000	-1.259990000
6	1.453961000	5.273624000	0.013019000
6	0.796522000	5.256205000	1.173326000

6	-0.675128000	5.219003000	1.259973000
8	-3.615242000	3.940284000	-2.197056000
6	-3.818892000	3.244424000	-3.408201000
8	-4.898876000	1.551036000	2.590689000
6	-4.415478000	2.086761000	3.803609000
8	-5.110377000	-0.942113000	-2.093334000
6	-4.698290000	-1.444635000	-3.343425000
8	-3.400992000	-3.374372000	2.540927000
6	-3.535235000	-2.708842000	3.775616000
8	-1.561792000	-4.619017000	-2.265719000
6	-0.964748000	-4.462715000	-3.532020000
8	4.898652000	1.555940000	-2.589993000
6	4.415080000	2.091067000	-3.803109000
8	3.610196000	3.943595000	2.197232000
6	3.813169000	3.247475000	3.408346000
8	1.224965000	5.176841000	-2.338281000
8	-1.230193000	5.176912000	2.338290000
8	1.566722000	-4.619539000	2.264916000
6	0.969448000	-4.463171000	3.531110000
6	2.910196000	-4.738316000	-0.169469000
6	5.008901000	-0.966837000	-0.249820000
6	4.572692000	-1.561873000	-1.425181000
6	3.859198000	-2.752591000	-1.412384000
6	3.599277000	-3.395349000	-0.203825000
6	4.011239000	-2.785629000	0.972597000
6	4.692137000	-1.576035000	0.961942000
8	3.405914000	-3.370600000	-2.541472000
6	3.539022000	-2.704181000	-3.775811000

8	5.110647000	-0.937283000	2.093954000	1	-3.067011000	-1.718724000	3.716729000
6	4.697741000	-1.439952000	3.343712000	1	-4.595367000	-2.563513000	4.016180000
1	-6.426080000	0.288744000	1.205021000	1	-0.355551000	-3.550636000	-3.544454000
1	-6.518567000	0.297147000	-0.550789000	1	-0.302552000	-5.309817000	-3.748224000
1	-4.998982000	1.633445000	-1.865214000	1	3.327598000	2.215121000	-3.749893000
1	-3.617926000	3.914731000	2.263458000	1	4.856188000	3.078569000	-3.982261000
1	-4.808520000	-1.069462000	2.355264000	1	3.349293000	2.256050000	3.345611000
1	-3.781441000	-3.293139000	-1.901681000	1	4.885881000	3.105692000	3.586370000
1	-3.284365000	-5.302661000	-0.685049000	1	0.359574000	-3.551542000	3.543094000
1	-3.166977000	-5.296507000	1.068828000	1	0.307834000	-5.310665000	3.747583000
1	1.142797000	-4.653099000	-2.175136000	1	3.172667000	-5.292939000	-1.070031000
1	-1.137694000	-4.656103000	2.174248000	1	3.290074000	-5.299766000	0.683817000
1	6.426707000	0.295314000	-1.203611000	1	4.811043000	-1.064383000	-2.354816000
1	6.518236000	0.303732000	0.552254000	1	3.784232000	-3.289663000	1.901355000
1	4.996606000	1.638337000	1.865955000	1	3.069727000	-1.714615000	-3.716159000
1	3.615086000	3.918284000	-2.263285000	1	4.598909000	-2.557535000	-4.016650000
1	3.205926000	5.904304000	-0.995160000	1	5.104910000	-2.445716000	3.503128000
1	3.329207000	5.903435000	0.767783000	1	3.603722000	-1.514027000	3.369951000
1	-3.335115000	5.900069000	-0.767996000	6	3.191123000	4.058160000	4.520646000
1	-3.211895000	5.901454000	0.994953000	1	2.117899000	4.171789000	4.362546000
1	-1.329607000	5.275429000	-2.118810000	1	3.346554000	3.557940000	5.477622000
1	1.324277000	5.278011000	2.118770000	1	3.642296000	5.049596000	4.572845000
1	-3.353969000	2.253429000	-3.346360000	6	-4.790240000	1.127486000	4.909030000
1	-4.891660000	3.101644000	-3.585070000	1	-5.872287000	1.002077000	4.960136000
1	-4.857544000	3.073821000	3.982819000	1	-4.336857000	0.149907000	4.737424000
1	-3.328138000	2.211942000	3.750044000	1	-4.439193000	1.507298000	5.869383000
1	-3.604234000	-1.517707000	-3.370792000	6	-2.864555000	-3.555048000	4.832381000
1	-5.104712000	-2.450794000	-3.502235000	1	-2.951134000	-3.074590000	5.807991000

1	-3.329436000	-4.539817000	4.888592000
1	-1.805763000	-3.685427000	4.604129000
6	2.074623000	-4.380682000	4.557949000
1	1.650637000	-4.263658000	5.556310000
1	2.681434000	-5.286540000	4.543219000
1	2.723312000	-3.527230000	4.355936000
6	5.195280000	-0.493922000	4.411477000
1	4.774577000	0.502436000	4.268552000
1	4.900715000	-0.854350000	5.398126000
1	6.282558000	-0.417286000	4.381022000
6	2.868976000	-3.550511000	-4.832882000
1	3.334883000	-4.534756000	-4.889738000
1	1.810377000	-3.682131000	-4.604455000
1	2.954824000	-3.069434000	-5.808250000
6	4.791175000	1.132082000	-4.908325000
1	4.338679000	0.154072000	-4.736833000
1	4.440112000	1.511500000	-5.868828000
6	-5.197787000	-0.499233000	-4.410836000
1	-4.903812000	-0.859492000	-5.397724000
1	-6.285109000	-0.423663000	-4.379341000
1	-4.777922000	0.497552000	-4.268432000
6	-3.198889000	4.056133000	-4.520892000
1	-2.125627000	4.170878000	-4.363856000
1	-3.354751000	3.556040000	-5.477863000
1	-3.651180000	5.047098000	-4.572320000
1	5.873362000	1.007718000	-4.959033000
6	-2.070104000	-4.381392000	-4.558762000
1	-2.719469000	-3.528422000	-4.356870000
1	-1.646334000	-4.264250000	-5.557202000
1	-2.676170000	-5.287744000	-4.543740000

P6Q2

Sum of electronic and zero-point Energies= -3150.401959

Sum of electronic and thermal Energies= -3150.333038

Sum of electronic and thermal Enthalpies= -3150.332094

Sum of electronic and thermal Free Energies= -3150.513820

6	5.844562000	0.000345000	0.000125000
6	5.022935000	-1.265729000	-0.013653000
6	4.620742000	-1.827411000	-1.216625000
6	3.906787000	-3.017248000	-1.255444000
6	3.624131000	-3.692596000	-0.070439000
6	3.996391000	-3.116372000	1.135622000
6	4.669662000	-1.902965000	1.174884000
6	5.022790000	1.266321000	0.013752000
6	4.620428000	1.828016000	1.216667000
6	3.906330000	3.017767000	1.255372000
6	3.623686000	3.693024000	0.070307000
6	3.996139000	3.116796000	-1.135689000
6	4.669563000	1.903467000	-1.174839000
6	2.951796000	5.046138000	0.103536000
6	-1.452675000	4.967467000	-0.109233000
6	-0.720493000	4.948231000	-1.223822000
6	0.753789000	4.915784000	-1.212946000
6	1.452097000	4.967684000	0.109256000
6	0.719916000	4.948719000	1.223849000
6	-0.754356000	4.915968000	1.212976000

6	-5.844581000	-0.000340000	0.000195000	8	-1.379156000	-4.878972000	-2.252433000
6	-5.022798000	-1.266309000	0.013788000	8	1.379735000	-4.878885000	2.252064000
6	-4.620527000	-1.828097000	1.216682000	8	-1.379669000	4.879000000	2.252301000
6	-3.906421000	-3.017852000	1.255348000	6	-2.952382000	5.045772000	-0.103532000
6	-3.623683000	-3.693007000	0.070257000	6	-5.022963000	1.265741000	-0.013551000
6	-3.996041000	-3.116679000	-1.135730000	6	-4.620886000	1.827533000	-1.216505000
6	-4.669468000	-1.903358000	-1.174832000	6	-3.906927000	3.017376000	-1.255286000
6	-2.951787000	-5.046118000	0.103425000	6	-3.624151000	3.692602000	-0.070244000
6	2.952384000	-5.045777000	-0.103810000	6	-3.996312000	3.116271000	1.135801000
6	1.452679000	-4.967484000	-0.109466000	6	-4.669587000	1.902868000	1.175018000
6	0.720468000	-4.948395000	-1.224039000	8	-3.474786000	3.603227000	-2.407225000
6	-0.753811000	-4.915897000	-1.213128000	6	-3.624341000	2.898032000	-3.621463000
6	-1.452087000	-4.967661000	0.109093000	8	-5.041705000	1.284300000	2.330525000
6	-0.719880000	-4.948538000	1.223667000	6	-4.629703000	1.838795000	3.561798000
6	0.754394000	-4.915870000	1.212757000	1	6.487580000	-0.004929000	0.879517000
8	3.474524000	-3.602976000	-2.407395000	1	6.487733000	0.005690000	-0.879159000
6	3.623993000	-2.897660000	-3.621570000	1	4.884761000	-1.304969000	-2.125339000
8	5.041887000	-1.284506000	2.330420000	1	3.742909000	-3.643988000	2.044359000
6	4.630026000	-1.839135000	3.561677000	1	4.884372000	1.305615000	2.125428000
8	5.042020000	1.285022000	-2.330309000	1	3.742679000	3.644335000	-2.044478000
6	4.629814000	1.839283000	-3.561617000	1	3.265312000	5.613552000	-0.772342000
8	3.473913000	3.603493000	2.407261000	1	3.274081000	5.583484000	0.994617000
6	3.623539000	2.898302000	3.621489000	1	-1.184628000	4.967467000	-2.202260000
8	1.379107000	4.878842000	-2.252265000	1	1.184044000	4.968324000	2.202281000
8	-5.041801000	-1.284810000	-2.330281000	1	-6.487755000	-0.005669000	-0.879087000
6	-4.629740000	-1.839158000	-3.561597000	1	-6.487599000	0.004907000	0.879588000
8	-3.474097000	-3.603687000	2.407227000	1	-4.884535000	-1.305776000	2.125469000
6	-3.623574000	-2.898519000	3.621488000	1	-3.742494000	-3.644147000	-2.044538000

1	-3.265338000	-5.613512000	-0.772452000	1	-3.048027000	-3.185697000	5.666490000
1	-3.274032000	-5.583485000	0.994507000	1	-3.378306000	-4.688175000	4.791992000
1	3.274688000	-5.582972000	-0.994975000	6	5.062093000	-0.893088000	4.657425000
1	3.266002000	-5.613283000	0.771974000	1	6.144503000	-0.761090000	4.648344000
1	1.184573000	-4.967754000	-2.202489000	1	4.593597000	0.083786000	4.527120000
1	-1.183990000	-4.968007000	2.202111000	1	4.768964000	-1.288858000	5.630627000
1	3.173990000	-1.902348000	-3.528030000	6	2.938499000	3.693574000	4.707489000
1	4.688000000	-2.764916000	-3.851011000	1	3.048143000	3.185387000	5.666545000
1	5.085998000	-2.826123000	3.701557000	1	3.378236000	4.687912000	4.792051000
1	3.542036000	-1.969456000	3.567602000	1	1.874061000	3.800475000	4.494738000
1	3.541788000	1.969312000	-3.567389000	6	-5.061648000	0.892628000	4.657489000
1	5.085501000	2.826360000	-3.701786000	1	-4.593067000	-0.084194000	4.527109000
1	3.173741000	1.902887000	3.528035000	1	-4.768544000	1.288357000	5.630715000
1	4.687574000	2.765800000	3.850930000	1	-6.144048000	0.760533000	4.648414000
1	-3.541729000	-1.969307000	-3.567440000	6	-2.939538000	3.693477000	-4.707486000
1	-5.085544000	-2.826193000	-3.701690000	1	-3.379414000	4.687768000	-4.791899000
1	-3.173716000	-1.903130000	3.528028000	1	-1.875089000	3.800498000	-4.494861000
1	-4.687578000	-2.765950000	3.851032000	1	-3.049248000	3.185369000	-5.666575000
1	-3.274707000	5.583047000	-0.994642000	6	-5.061861000	-0.892997000	-4.657223000
1	-3.265960000	5.613210000	0.772309000	1	-4.593463000	0.083906000	-4.526782000
1	-4.884975000	1.305171000	-2.125244000	1	-4.768676000	-1.288610000	-5.630471000
1	-3.742737000	3.643807000	2.044559000	6	5.061968000	0.893138000	-4.657246000
1	-3.174325000	1.902711000	-3.528058000	1	4.768558000	1.288633000	-5.630475000
1	-4.688361000	2.765305000	-3.850846000	1	6.144417000	0.761453000	-4.648323000
1	-5.085658000	2.825768000	3.701837000	1	4.593777000	-0.083847000	-4.526673000
1	-3.541713000	1.969115000	3.567608000	6	2.939099000	-3.692983000	-4.707624000
6	-2.938503000	-3.693868000	4.707413000	1	1.874683000	-3.800088000	-4.494875000
1	-1.874090000	-3.800843000	4.494571000	1	3.048656000	-3.184721000	-5.666649000

1 3.379015000 -4.687238000 -4.792246000

1 -6.144284000 -0.761102000 -4.648142000

P6Q3

Sum of electronic and zero-point Energies= -2992.055574

Sum of electronic and thermal Energies= -2991.992730

Sum of electronic and thermal Enthalpies= -2991.991786

Sum of electronic and thermal Free Energies= -2992.161175

6	3.190617000	-4.912224000	-0.047241000
6	3.798745000	-3.529195000	-0.023351000
6	4.098295000	-2.910237000	1.181317000
6	4.714473000	-1.666456000	1.215537000
6	5.080417000	-1.044241000	0.022966000
6	4.749419000	-1.646991000	-1.181674000
6	4.093156000	-2.870057000	-1.215883000
6	1.689123000	-4.902516000	-0.084427000
6	0.980098000	-4.913170000	-1.213956000
6	-0.494194000	-4.948041000	-1.232049000
6	-1.215144000	-5.042759000	0.075563000
6	-0.508071000	-4.991618000	1.205226000
6	0.962706000	-4.884549000	1.223528000
6	-2.708799000	-5.196092000	0.037914000
6	-4.958536000	-1.525513000	-0.015712000
6	-4.568039000	-2.097195000	1.186221000
6	-3.798544000	-3.252556000	1.215001000
6	-3.446335000	-3.877408000	0.019684000
6	-3.807149000	-3.286561000	-1.182290000
6	-4.538817000	-2.106902000	-1.211111000
6	-3.145606000	4.942263000	0.039388000
6	-1.634902000	4.921289000	0.018629000
6	-0.945036000	4.933802000	-1.184667000
6	0.442362000	4.977879000	-1.216129000
6	1.157880000	5.055345000	-0.022268000
6	0.469659000	5.007403000	1.181060000
6	-0.915579000	4.918328000	1.212675000
6	2.659632000	5.220908000	-0.043377000
6	5.854264000	0.253362000	0.046653000
6	4.975102000	1.470575000	0.082710000
6	4.572557000	2.055479000	1.211664000
6	3.745132000	3.276206000	1.228505000
6	3.402510000	3.916070000	-0.079867000
6	3.771077000	3.309277000	-1.208861000
6	4.538424000	2.049951000	-1.225778000
8	5.018911000	-1.003383000	2.363549000
6	4.613889000	-1.560059000	3.599254000
8	3.728972000	-3.502159000	-2.363979000
6	3.945134000	-2.847732000	-3.599277000
8	1.564798000	-4.813538000	2.274575000
8	-1.100691000	-4.929865000	-2.282805000
8	-3.372544000	-3.850588000	2.360069000
6	-3.645464000	-3.223234000	3.598192000
8	-1.644604000	4.852137000	2.359197000
6	-0.963042000	4.778453000	3.596545000
8	1.174459000	4.976938000	-2.362538000
6	0.502958000	4.832763000	-3.599171000
8	3.378975000	3.761074000	2.278766000

8	4.829930000	1.517150000	-2.276214000	1	6.501644000	0.266676000	0.922388000
8	-4.908454000	-1.472524000	-2.356211000	1	6.478862000	0.303946000	-0.844691000
6	-4.452669000	-1.982105000	-3.594589000	1	4.848766000	1.658819000	2.181005000
6	-5.852183000	-0.307277000	-0.033660000	1	3.527664000	3.726766000	-2.178220000
6	-3.759868000	3.572205000	0.079753000	1	3.534636000	-1.746872000	3.586594000
6	-4.063289000	2.933933000	1.210797000	1	5.119458000	-2.519106000	3.759609000
6	-4.706084000	1.606758000	1.231821000	1	5.019408000	-2.704745000	-3.762317000
6	-5.092755000	0.987897000	-0.074390000	1	3.473073000	-1.859413000	-3.583512000
6	-4.753099000	1.607464000	-1.205508000	1	-3.269169000	-2.194613000	3.584257000
6	-4.044580000	2.900711000	-1.226685000	1	-4.727883000	-3.183166000	3.765071000
8	-4.938995000	1.048536000	2.283676000	1	-0.261098000	3.937640000	3.584423000
8	-3.728335000	3.415845000	-2.278778000	1	-0.386266000	5.695955000	3.759606000
1	3.555894000	-5.447354000	-0.922701000	1	-0.115492000	3.928828000	-3.582791000
1	3.511335000	-5.450292000	0.844251000	1	-0.158892000	5.690168000	-3.766119000
1	3.836150000	-3.428550000	2.092937000	1	-3.360618000	-2.066521000	-3.582124000
1	5.019679000	-1.132952000	-2.093337000	1	-4.865581000	-2.983698000	-3.760117000
1	1.464222000	-4.906806000	-2.182871000	1	-6.473292000	-0.316371000	0.861480000
1	-0.990270000	-5.037043000	2.174062000	1	-6.503287000	-0.355313000	-0.905457000
1	-3.021055000	-5.767306000	0.911111000	1	-3.856213000	3.373857000	2.178708000
1	-2.976508000	-5.758710000	-0.855926000	1	-4.994856000	1.185397000	-2.173319000
1	-4.882848000	-1.613074000	2.099965000	6	1.550099000	4.745213000	-4.683822000
1	-3.499832000	-3.775266000	-2.096094000	1	2.193039000	3.877484000	-4.530534000
1	-3.482495000	5.499446000	0.912478000	1	1.068849000	4.648302000	-5.657803000
1	-3.500321000	5.454496000	-0.854444000	1	2.171288000	5.641524000	-4.693424000
1	-1.523720000	4.908404000	-2.097294000	6	3.348123000	-3.708733000	-4.686884000
1	1.047839000	5.041593000	2.093661000	1	3.812218000	-4.695577000	-4.695895000
1	2.963518000	5.767093000	0.849113000	1	2.274466000	-3.830105000	-4.537384000
1	2.941731000	5.805534000	-0.917968000	1	3.508900000	-3.242731000	-5.659824000

6	-4.903646000	-1.031073000	-4.677556000
1	-4.583034000	-1.399699000	-5.652718000
1	-5.990415000	-0.940315000	-4.683124000
1	-4.472193000	-0.040778000	-4.525919000
6	-1.997603000	4.596196000	4.681514000
1	-2.554263000	3.670050000	4.532444000
1	-1.509732000	4.550540000	5.655950000
6	4.973821000	-0.576258000	4.687027000
1	4.451285000	0.369880000	4.540049000
1	4.689376000	-0.978122000	5.660282000
1	6.047260000	-0.383898000	4.693315000
6	-2.965888000	-4.027147000	4.681040000
1	-1.886045000	-4.045662000	4.527992000
1	-3.166951000	-3.581569000	5.656059000
1	-3.334261000	-5.053590000	4.688041000
1	-2.701842000	5.428857000	4.686636000

MeP5 with one $AgOCOCF_3$

Sum of electronic and zero-point Energies= -3169.668757

Sum of electronic and thermal Energies= -3169.605909

Sum of electronic and thermal Enthalpies= -3169.604964

Sum of electronic and thermal Free Energies= -3169.772682

9	-1.913809000	0.624162000	5.236073000
9	-0.189404000	-0.681616000	5.269384000
9	0.054342000	1.432994000	4.925042000
8	-0.062865000	0.629875000	2.377001000
6	-0.867302000	0.111055000	3.141569000
6	-0.731137000	0.386678000	4.664432000
47	-1.672855000	-0.946309000	0.693985000
8	1.405554000	4.174832000	1.708733000
8	-0.797600000	3.768330000	-3.321459000
8	3.861772000	-0.037258000	2.605675000
8	4.276736000	2.194703000	-2.413725000
8	0.583628000	-3.642461000	1.371894000
8	4.955861000	-2.862900000	-1.885709000
8	-4.426271000	-3.927371000	-0.417630000
8	0.379233000	-1.874884000	-2.122772000
8	-3.412598000	2.950517000	1.137081000
8	-4.000019000	0.085557000	-3.524092000
6	-2.572137000	4.199715000	-1.208164000
1	-2.784960000	4.687560000	-2.158889000
1	-2.993473000	4.814630000	-0.413970000
6	-1.076061000	4.096590000	-1.011455000
6	-0.531016000	4.189180000	0.260621000
1	-1.208752000	4.336695000	1.088594000
6	0.835864000	4.083780000	0.476821000
6	1.697166000	3.900570000	-0.603077000
6	1.151844000	3.792351000	-1.875170000
1	1.834577000	3.634488000	-2.698872000
6	-0.217673000	3.877321000	-2.087528000
6	0.563593000	4.095285000	2.835779000
1	-0.042811000	3.186817000	2.805231000
1	-0.081949000	4.975816000	2.919431000
6	0.035589000	3.526035000	-4.424583000
1	0.588419000	2.586700000	-4.314234000
1	0.750003000	4.340943000	-4.580263000

6	3.191272000	3.811783000	-0.402534000	6	4.941231000	-3.363371000	-3.196630000
1	3.689908000	4.210273000	-1.284548000	1	4.120197000	-2.936393000	-3.783195000
1	3.474338000	4.433796000	0.446311000	1	4.859129000	-4.455262000	-3.212429000
6	3.672746000	2.401868000	-0.150327000	6	0.391600000	-4.603255000	-1.229986000
6	3.561524000	1.846507000	1.115976000	1	0.152744000	-5.497541000	-0.649915000
1	3.105638000	2.449965000	1.886648000	1	0.584175000	-4.933394000	-2.253971000
6	3.989892000	0.554459000	1.386388000	6	-0.827617000	-3.709523000	-1.222617000
6	4.585218000	-0.200598000	0.378545000	6	-2.058035000	-4.226868000	-0.826310000
6	4.691804000	0.350782000	-0.891771000	1	-2.091406000	-5.262866000	-0.518510000
1	5.133046000	-0.261898000	-1.665729000	6	-3.210360000	-3.457043000	-0.783100000
6	4.225796000	1.628709000	-1.169331000	6	-3.162554000	-2.098735000	-1.126074000
6	3.166969000	0.666935000	3.612325000	6	-1.923372000	-1.573418000	-1.528230000
1	2.158605000	0.933441000	3.286284000	1	-1.886211000	-0.576105000	-1.949854000
1	3.708762000	1.570128000	3.911953000	6	-0.770443000	-2.372279000	-1.603747000
6	5.013201000	-1.629900000	0.622208000	6	-4.531307000	-5.267535000	0.001948000
1	5.226121000	-1.776686000	1.679507000	1	-3.907809000	-5.460980000	0.880088000
1	5.919991000	-1.844173000	0.059537000	1	-4.254961000	-5.960347000	-0.798801000
6	3.901318000	-2.554466000	0.190947000	6	0.908092000	-0.690495000	-1.542193000
6	2.817836000	-2.765767000	1.030701000	1	0.989963000	-0.796437000	-0.454708000
1	2.857274000	-2.323320000	2.015643000	1	0.306229000	0.190860000	-1.781069000
6	1.692704000	-3.457049000	0.603209000	6	-4.439850000	-1.278376000	-1.152213000
6	1.643899000	-3.961429000	-0.693552000	1	-5.024387000	-1.590296000	-2.019987000
6	2.749726000	-3.797229000	-1.513738000	1	-5.032332000	-1.528359000	-0.271793000
1	2.690986000	-4.178717000	-2.523611000	6	-4.164908000	0.196330000	-1.193163000
6	3.869586000	-3.097244000	-1.091673000	6	-4.040379000	0.911038000	-0.005679000
6	0.582479000	-3.090055000	2.670510000	1	-4.308110000	0.416306000	0.919595000
1	0.768725000	-2.012604000	2.644738000	6	-3.569491000	2.218545000	0.007352000
1	1.334468000	-3.567359000	3.306299000	6	-3.215496000	2.835264000	-1.195596000

6	-3.397598000	2.140209000	-2.380291000
1	-3.113312000	2.634494000	-3.297552000
6	-3.866245000	0.832630000	-2.392135000
6	-3.736661000	2.355113000	2.372945000
1	-3.160298000	1.443395000	2.547786000
1	-4.804350000	2.120522000	2.432481000
6	-3.584153000	0.648297000	-4.743505000
1	-2.524578000	0.924215000	-4.716197000
1	-4.176284000	1.531389000	-5.003443000
8	-1.810412000	-0.674469000	2.858163000
6	4.836448000	1.443113000	-3.457331000
1	5.884549000	1.193674000	-3.260974000
1	4.279768000	0.515276000	-3.631936000
1	-3.735985000	-0.118574000	-5.499797000
1	1.909568000	-0.562821000	-1.947813000
1	-0.617642000	3.457683000	-5.292826000
1	4.780352000	2.065815000	-4.348297000
1	5.889274000	-3.072979000	-3.645116000
1	1.219743000	4.055952000	3.702824000
1	-3.489883000	3.088459000	3.137159000
1	-5.575578000	-5.421167000	0.262821000
1	-0.408928000	-3.266494000	3.079940000
1	3.105688000	-0.006665000	4.464906000

EtP5 with one $AgOCOCF_3$

Sum of electronic and zero-point Energies= -3562.603384

Sum of electronic and thermal Energies= -3562.527792

Sum of electronic and thermal Enthalpies= -3562.526847

Sum of electronic and thermal Free Energies= -3562.720517

9	-4.312378000	2.946109000	-0.938350000
9	-5.238238000	1.618372000	0.480990000
9	-5.059516000	1.030352000	-1.588806000
8	-3.196946000	-0.186942000	0.130814000
8	-2.032485000	1.622231000	-0.479608000
6	-3.060279000	0.993988000	-0.288276000
6	-4.431342000	1.666877000	-0.587561000
47	-1.238950000	-1.110882000	0.614568000
8	2.352508000	-3.360689000	-3.252629000
8	-1.156856000	0.753556000	-4.282783000
8	1.407542000	-4.540111000	1.528598000
8	-2.803575000	-3.383660000	-1.825998000
8	2.314248000	-0.547824000	4.408324000
8	-2.889970000	-1.811225000	3.133745000
8	4.018926000	3.223997000	1.670149000
8	-1.295476000	2.993909000	3.088435000
8	3.929419000	1.394235000	-3.119192000
8	-0.082745000	4.747704000	-1.391779000
6	1.550499000	1.336927000	-4.566628000
1	0.835831000	1.801825000	-5.244847000
1	2.492879000	1.225267000	-5.102406000
6	1.048148000	-0.032689000	-4.174869000
6	1.941506000	-1.062046000	-3.917805000
1	2.995522000	-0.840131000	-4.004854000
6	1.505477000	-2.325933000	-3.542927000
6	0.142589000	-2.590790000	-3.453758000
6	-0.754329000	-1.559228000	-3.700430000

1	-1.809019000	-1.776716000	-3.607684000	6	1.043162000	-0.881388000	4.058366000
6	-0.317825000	-0.286586000	-4.043388000	6	0.075859000	0.117880000	4.190352000
6	3.730970000	-3.089544000	-3.148132000	6	-1.238598000	-0.183734000	3.869442000
1	3.895318000	-2.279531000	-2.426573000	1	-1.975497000	0.599133000	3.977009000
1	4.129457000	-2.759335000	-4.114753000	6	-1.608752000	-1.450991000	3.417330000
6	-2.535841000	0.586157000	-4.026762000	6	3.344479000	-1.486903000	4.198416000
1	-2.680488000	0.243172000	-2.996040000	1	3.388041000	-1.755436000	3.135355000
1	-2.951938000	-0.174875000	-4.698422000	1	3.144306000	-2.402318000	4.768059000
6	-0.362358000	-3.952251000	-3.035423000	6	-3.906087000	-0.820252000	3.177773000
1	-1.303709000	-4.160466000	-3.540826000	1	-3.607443000	0.036789000	2.569036000
1	0.354360000	-4.718223000	-3.327046000	1	-4.047114000	-0.488031000	4.212235000
6	-0.562417000	-3.996309000	-1.541792000	6	0.459314000	1.506221000	4.642452000
6	0.501192000	-4.302124000	-0.706936000	1	1.254621000	1.438218000	5.384155000
1	1.442320000	-4.549628000	-1.176934000	1	-0.402378000	1.974037000	5.117029000
6	0.385315000	-4.255452000	0.676076000	6	0.927283000	2.365352000	3.490962000
6	-0.832097000	-3.901515000	1.256382000	6	2.271316000	2.417397000	3.145478000
6	-1.914481000	-3.606182000	0.417960000	1	2.965065000	1.836749000	3.736176000
1	-2.872167000	-3.406156000	0.880748000	6	2.711681000	3.171804000	2.067422000
6	-1.791608000	-3.652876000	-0.973329000	6	1.804218000	3.928332000	1.330404000
6	2.693094000	-4.769731000	0.990750000	6	0.462923000	3.881397000	1.680127000
1	3.001464000	-3.905748000	0.389563000	1	-0.230311000	4.447122000	1.075531000
1	2.675841000	-5.646818000	0.333665000	6	0.014580000	3.096745000	2.733510000
6	-0.989567000	-3.818255000	2.756485000	6	4.917184000	2.277866000	2.195802000
1	-0.343551000	-4.555939000	3.229988000	1	4.506436000	1.267860000	2.068673000
1	-2.019345000	-4.050715000	3.022496000	1	5.064970000	2.444404000	3.270316000
6	-0.632696000	-2.442584000	3.270521000	6	-2.257435000	3.676484000	2.305761000
6	0.684131000	-2.138465000	3.599382000	1	-2.176971000	3.356999000	1.261867000
1	1.419453000	-2.920581000	3.480010000	1	-2.072686000	4.756476000	2.351259000

6	2.246187000	4.720278000	0.122278000	6	-3.621177000	3.350321000	2.864243000
1	1.646741000	5.626443000	0.048173000	1	-3.700800000	3.666312000	3.905202000
1	3.288733000	5.013521000	0.233929000	1	-4.392090000	3.858199000	2.283980000
6	2.084912000	3.898237000	-1.133957000	1	-3.812941000	2.278083000	2.810202000
6	3.107428000	3.062662000	-1.560898000	6	-2.274685000	5.606963000	-1.196279000
1	4.024363000	3.062837000	-0.988418000	1	-3.284265000	5.570336000	-1.606522000
6	2.951729000	2.236647000	-2.664641000	1	-2.328330000	5.343135000	-0.139322000
6	1.752974000	2.242488000	-3.374639000	1	-1.898617000	6.627278000	-1.282171000
6	0.734015000	3.083250000	-2.950689000	6	-3.211057000	1.917726000	-4.247668000
1	-0.197372000	3.060752000	-3.497715000	1	-3.041377000	2.269707000	-5.265916000
6	0.885271000	3.905388000	-1.842233000	1	-4.284291000	1.823287000	-4.083564000
6	5.117893000	1.281792000	-2.374899000	1	-2.823602000	2.660107000	-3.550065000
1	4.888577000	0.960624000	-1.350572000	6	4.643731000	-0.861613000	4.647752000
1	5.622352000	2.253804000	-2.312656000	1	4.854271000	0.042937000	4.076702000
6	-1.383258000	4.640897000	-1.939240000	1	4.597857000	-0.599630000	5.704979000
1	-1.742183000	3.614941000	-1.819587000	1	5.465978000	-1.563114000	4.499929000
1	-1.361038000	4.888896000	-3.007389000	6	3.644430000	-4.991227000	2.142317000
6	-4.069108000	-2.993110000	-1.309543000	1	3.673774000	-4.116046000	2.792659000
1	-4.513987000	-3.832022000	-0.761924000	1	3.336562000	-5.852399000	2.735676000
1	-3.946731000	-2.147194000	-0.629385000	1	4.651104000	-5.173227000	1.763918000
6	-5.165552000	-1.429946000	2.616313000	6	4.421845000	-4.356012000	-2.699277000
1	-5.457494000	-2.316218000	3.181141000	1	4.043437000	-4.678638000	-1.727865000
1	-5.977359000	-0.703354000	2.660976000	1	4.256273000	-5.159875000	-3.416949000
1	-5.011766000	-1.698961000	1.571003000	1	5.495839000	-4.185810000	-2.611504000
6	-4.930197000	-2.590302000	-2.481349000	6	6.006218000	0.273171000	-3.065611000
1	-5.045467000	-3.413778000	-3.186958000	1	6.941957000	0.158934000	-2.516341000
1	-5.917079000	-2.290918000	-2.127220000	1	5.515929000	-0.699882000	-3.119079000
1	-4.485937000	-1.740322000	-3.001174000	1	6.237647000	0.597428000	-4.080568000

6	6.228292000	2.419799000	1.458111000
1	6.090717000	2.234520000	0.391741000
1	6.632155000	3.424772000	1.583030000
1	6.955680000	1.703619000	1.843658000

EtP6 with one $AgOCOCF_3$

Sum of electronic and zero-point Energies= -4140.452649

Sum of electronic and thermal Energies= -4140.362407

Sum of electronic and thermal Enthalpies= -4140.361463

Sum of electronic and thermal Free Energies= -4140.587229

6	-6.028177000	0.618176000	0.265736000
6	-5.073312000	1.787339000	0.254701000
6	-4.602957000	2.294400000	-0.947121000
6	-3.749672000	3.388068000	-0.989292000
6	-3.384939000	4.028545000	0.191968000
6	-3.834582000	3.506908000	1.397297000
6	-4.651427000	2.385588000	1.439955000
6	-5.344314000	-0.727474000	0.271477000
6	-5.044521000	-1.356416000	1.471716000
6	-4.453263000	-2.611482000	1.502791000
6	-4.177505000	-3.280482000	0.312561000
6	-4.452167000	-2.639864000	-0.886855000
6	-5.014195000	-1.371013000	-0.919121000
6	-3.619686000	-4.683217000	0.319896000
6	0.678949000	-5.055047000	0.396470000
6	-0.002688000	-4.947720000	-0.807494000
6	-1.381065000	-4.784139000	-0.847963000
6	-2.111963000	-4.756578000	0.338424000
6	-1.426454000	-4.831390000	1.542456000
6	-0.045520000	-4.964184000	1.582180000
6	5.797972000	-0.780258000	0.732307000
6	5.123799000	0.562256000	0.627450000
6	4.628595000	1.189048000	1.760503000
6	3.963493000	2.408247000	1.700807000
6	3.793533000	3.048890000	0.471240000
6	4.283859000	2.411489000	-0.686217000
6	4.948489000	1.175835000	-0.614793000
6	3.232890000	4.451171000	0.393904000
6	-2.526464000	5.269953000	0.168521000
6	-1.047041000	4.982422000	0.247225000
6	-0.324716000	4.714093000	-0.904461000
6	1.041652000	4.477876000	-0.862362000
6	1.726499000	4.559130000	0.349031000
6	0.997052000	4.792815000	1.507931000
6	-0.377624000	4.979222000	1.470106000
8	-3.252026000	3.906338000	-2.149087000
6	-3.449826000	3.177421000	-3.342664000
8	-5.103429000	1.826617000	2.601162000
6	-4.571087000	2.283766000	3.821238000
8	-5.298516000	-0.703616000	-2.073862000
6	-4.876774000	-1.265010000	-3.298238000
8	-4.139910000	-3.268232000	2.659519000
6	-4.292648000	-2.588041000	3.882122000
8	-2.092494000	-4.678209000	-2.004110000
6	-1.389149000	-4.620428000	-3.226184000
8	5.477762000	0.553956000	-1.688476000

6	5.092408000	0.966904000	-2.992249000	1	0.578049000	-5.003343000	-1.717386000
8	3.483976000	3.059399000	2.789607000	1	-2.010383000	-4.788491000	2.451020000
6	3.583403000	2.434763000	4.054310000	1	6.520680000	-0.888628000	-0.074720000
8	1.786638000	4.171687000	-1.968634000	1	6.339032000	-0.833910000	1.675586000
6	1.104021000	3.945902000	-3.192964000	1	4.763880000	0.678603000	2.703800000
8	-1.137785000	5.196587000	2.580246000	1	4.274312000	2.972591000	-1.612556000
6	-0.570014000	4.951458000	3.846205000	1	3.643867000	4.931896000	-0.494174000
8	0.670939000	-5.049881000	2.743732000	1	3.591089000	5.005650000	1.261330000
6	-0.006113000	-4.877463000	3.965716000	1	-2.723023000	5.821098000	-0.750721000
6	2.165893000	-5.315078000	0.413191000	1	-2.810409000	5.908795000	1.003648000
6	4.799868000	-1.911603000	0.657874000	1	-0.867716000	4.686864000	-1.837885000
6	4.371133000	-2.387974000	-0.573984000	1	1.537015000	4.832363000	2.443540000
6	3.472651000	-3.442024000	-0.668688000	1	-3.094650000	2.150460000	-3.209292000
6	3.029107000	-4.079235000	0.491245000	1	-4.518030000	3.139315000	-3.587736000
6	3.428549000	-3.576194000	1.720440000	1	-4.854685000	3.329615000	3.993680000
6	4.281767000	-2.484928000	1.814291000	1	-3.475363000	2.235537000	3.790095000
8	3.009993000	-3.929430000	-1.848306000	1	-3.801221000	-1.463207000	-3.261783000
6	3.333887000	-3.237475000	-3.040808000	1	-5.391831000	-2.217267000	-3.473761000
8	4.676646000	-1.933201000	3.002551000	1	-3.720813000	-1.651933000	3.861133000
6	4.044430000	-2.366426000	4.185298000	1	-5.346299000	-2.331378000	4.047289000
1	-6.667117000	0.688041000	1.145397000	1	-0.645164000	-3.819586000	-3.187285000
1	-6.669083000	0.686834000	-0.613033000	1	-0.861248000	-5.565798000	-3.401423000
1	-4.919796000	1.799378000	-1.853680000	1	3.999274000	0.985366000	-3.055862000
1	-3.526963000	4.006975000	2.305273000	1	5.478731000	1.971716000	-3.195306000
1	-5.291175000	-0.832845000	2.384876000	1	3.066779000	1.468329000	4.029509000
1	-4.208540000	-3.164034000	-1.799956000	1	4.635531000	2.249648000	4.299181000
1	-3.986280000	-5.204373000	-0.564227000	1	0.309391000	3.208980000	-3.035119000
1	-4.001677000	-5.210805000	1.193780000	1	0.646358000	4.880778000	-3.534568000

1	0.230307000	5.672275000	4.053439000	6	-5.112942000	1.401346000	4.921799000
1	-0.129578000	3.947120000	3.861064000	1	-6.202054000	1.446386000	4.949035000
1	-0.517020000	-3.906723000	3.973862000	1	-4.814468000	0.364146000	4.762112000
1	-0.768138000	-5.655833000	4.093084000	1	-4.728180000	1.727819000	5.889174000
1	2.428107000	-5.866938000	-0.489126000	6	-3.792198000	-3.495167000	4.982178000
1	2.401481000	-5.951276000	1.266546000	1	-3.898218000	-3.004701000	5.951000000
1	4.762248000	-1.913803000	-1.462847000	1	-4.360486000	-4.425647000	5.000795000
1	3.050564000	-4.066855000	2.606300000	1	-2.739380000	-3.737177000	4.830035000
1	3.054845000	-2.182650000	-2.951050000	6	1.015130000	-4.952991000	5.076858000
1	4.414825000	-3.299510000	-3.220469000	1	0.526639000	-4.829589000	6.044551000
1	4.281836000	-3.418832000	4.381075000	1	1.523942000	-5.917422000	5.066085000
1	2.956361000	-2.285268000	4.074432000	1	1.763434000	-4.167045000	4.965063000
8	1.779429000	-0.143672000	-3.319768000	6	4.533814000	-1.494857000	5.318282000
6	0.681350000	0.009550000	-2.799779000	1	4.277824000	-0.449325000	5.137359000
47	2.180519000	1.486563000	-0.940812000	1	4.071707000	-1.804983000	6.256523000
8	0.379386000	0.653581000	-1.759932000	1	5.616378000	-1.571715000	5.423622000
6	-0.557205000	-0.625970000	-3.492138000	6	2.571728000	-3.882987000	-4.172348000
9	-1.307442000	0.332419000	-4.067011000	1	2.834242000	-4.937407000	-4.269221000
9	-0.218757000	-1.487240000	-4.452593000	1	1.498247000	-3.800972000	-4.002408000
9	-1.332699000	-1.277204000	-2.623888000	1	2.803096000	-3.375003000	-5.109162000
6	2.954766000	3.353844000	5.073642000	6	5.649425000	-0.031349000	-3.975987000
1	1.904309000	3.526372000	4.838545000	1	5.232203000	-1.021059000	-3.789045000
1	3.017706000	2.906853000	6.066496000	1	5.378596000	0.264900000	-4.989788000
1	3.468917000	4.315187000	5.093845000	6	-5.196403000	-0.279039000	-4.396638000
6	-1.667441000	5.069812000	4.877642000	1	-4.894978000	-0.687470000	-5.362241000
1	-2.111563000	6.065239000	4.853588000	1	-6.265608000	-0.066017000	-4.427198000
1	-2.452450000	4.336582000	4.687435000	1	-4.658862000	0.656527000	-4.238772000
1	-1.264012000	4.894463000	5.876007000	6	-2.683202000	3.866893000	-4.445800000

1	-1.616224000	3.881421000	-4.221469000
1	-2.825881000	3.332664000	-5.386027000
1	-3.026462000	4.894239000	-4.573201000
6	2.103822000	3.432501000	-4.200067000
1	2.485076000	2.455344000	-3.900522000
1	1.620092000	3.314855000	-5.170437000
1	2.936711000	4.128510000	-4.310223000
6	-2.388707000	-4.353776000	-4.325652000
1	-2.889926000	-3.399866000	-4.158892000
1	-1.878669000	-4.307956000	-5.288645000
1	-3.141302000	-5.142252000	-4.365578000
1	6.736201000	-0.086560000	-3.905691000

P6Q1 with one AgOCOCF$_3$

Sum of electronic and zero-point Energies= -3982.098896

Sum of electronic and thermal Energies= -3982.015577

Sum of electronic and thermal Enthalpies= -3982.014633

Sum of electronic and thermal Free Energies= -3982.225438

6	-5.823387000	1.415300000	0.105338000
6	-5.365142000	-0.003269000	0.342330000
6	-5.118721000	-0.449537000	1.632127000
6	-4.722625000	-1.755176000	1.881572000
6	-4.615308000	-2.657027000	0.826858000
6	-4.836709000	-2.208729000	-0.467460000
6	-5.185843000	-0.888386000	-0.720674000
6	-4.690213000	2.406042000	-0.010615000
6	-4.127841000	2.684738000	-1.247695000
6	-3.108225000	3.616329000	-1.386329000
6	-2.654535000	4.320510000	-0.272708000
6	-3.198513000	4.021896000	0.968347000
6	-4.194056000	3.065815000	1.110983000
6	-1.629846000	5.419893000	-0.414594000
6	2.542005000	4.309972000	-0.298033000
6	1.837951000	4.557628000	0.873844000
6	0.486042000	4.871563000	0.857110000
6	-0.190439000	4.967902000	-0.358470000
6	0.503960000	4.688480000	-1.524633000
6	1.851844000	4.354996000	-1.506249000
6	5.674409000	-1.542679000	0.082636000
6	4.577004000	-2.550159000	0.306655000
6	4.325108000	-3.559987000	-0.596738000
6	3.297037000	-4.485245000	-0.422294000
6	2.479187000	-4.416487000	0.696435000
6	2.699419000	-3.373013000	1.627902000
6	3.762844000	-2.471783000	1.451095000
6	1.458339000	-5.495225000	0.973798000
6	-4.280198000	-4.105953000	1.092230000
6	-2.802262000	-4.366367000	1.095508000
6	-2.030827000	-4.218071000	2.174443000
6	-0.589800000	-4.505361000	2.169635000
6	0.019571000	-5.060418000	0.923645000
6	-0.735637000	-5.165671000	-0.169171000
6	-2.176336000	-4.830047000	-0.182972000
8	-4.445957000	-2.237150000	3.129076000
6	-4.439900000	-1.332771000	4.211859000
8	-5.389753000	-0.389521000	-1.967767000

6	-5.128013000	-1.216194000	-3.084337000	1	-4.506846000	2.142585000	-2.102612000
8	-4.757851000	2.739488000	2.313177000	1	-2.821664000	4.566053000	1.822836000
6	-4.198124000	3.278694000	3.487089000	1	-1.798038000	6.150030000	0.377220000
8	-2.520934000	3.918784000	-2.578037000	1	-1.790842000	5.926837000	-1.366117000
6	-2.921424000	3.207555000	-3.729524000	1	2.383714000	4.514727000	1.807014000
8	-0.244353000	5.133407000	1.978533000	1	-0.041181000	4.747320000	-2.455954000
8	4.098366000	-1.542588000	2.360203000	1	6.412504000	-1.631344000	0.882916000
6	3.158883000	-1.158506000	3.350276000	1	6.179794000	-1.798546000	-0.850910000
8	3.103649000	-5.468766000	-1.347624000	1	4.926947000	-3.629159000	-1.494533000
6	2.344741000	-5.052143000	-2.487572000	1	2.158780000	-3.410836000	2.565360000
8	0.088150000	-4.307959000	3.160794000	1	1.658480000	-5.900861000	1.967419000
8	-2.833976000	-4.984382000	-1.186161000	1	1.608135000	-6.290340000	0.246836000
8	2.572554000	4.091179000	-2.635812000	1	-4.688240000	-4.398621000	2.058850000
6	4.025857000	4.040692000	-0.241977000	1	-4.739294000	-4.722315000	0.320607000
6	5.205665000	-0.108440000	0.024185000	1	-2.442930000	-3.875048000	3.115281000
6	5.669858000	0.821419000	0.942072000	1	-0.343494000	-5.537560000	-1.107908000
6	5.277419000	2.152981000	0.895162000	1	-3.760559000	-0.499870000	3.994551000
6	4.406668000	2.583285000	-0.105785000	1	-5.443334000	-0.915929000	4.359831000
6	3.929217000	1.647685000	-1.011891000	1	-5.869044000	-2.023139000	-3.131497000
6	4.312079000	0.315486000	-0.956557000	1	-4.138085000	-1.670525000	-2.991370000
8	5.699964000	3.089917000	1.793182000	1	-3.129030000	3.036980000	3.532446000
6	6.619404000	2.709507000	2.791577000	1	-4.292992000	4.371275000	3.488920000
8	3.871642000	-0.630436000	-1.829908000	1	-2.806352000	2.132556000	-3.561348000
6	2.966652000	-0.233246000	-2.846550000	1	-3.978615000	3.405811000	-3.944150000
1	-6.412246000	1.450518000	-0.810080000	1	2.186478000	-0.978706000	2.872614000
1	-6.473459000	1.712043000	0.928182000	1	3.030345000	-1.963381000	4.080858000
1	-5.241632000	0.260655000	2.437303000	1	1.485011000	-4.457792000	-2.157841000
1	-4.729238000	-2.920463000	-1.274379000	1	2.967826000	-4.408716000	-3.117501000

1	4.449905000	4.594815000	0.592444000
1	4.489926000	4.428699000	-1.151069000
1	6.340638000	0.475421000	1.715906000
1	3.256789000	1.994187000	-1.781574000
1	6.193150000	1.912337000	3.412998000
1	7.534753000	2.320155000	2.329801000
1	3.453018000	0.498245000	-3.501961000
1	2.087304000	0.235630000	-2.396892000
9	-1.662323000	-1.197647000	-3.787540000
9	-0.453392000	0.500848000	-3.257621000
9	-2.235668000	0.045614000	-2.123643000
8	0.214252000	-0.692436000	-0.884663000
8	-0.330278000	-2.587477000	-1.950705000
6	-0.359704000	-1.379133000	-1.777205000
6	-1.192040000	-0.501591000	-2.753335000
47	1.399634000	-1.979941000	0.321059000
6	1.901960000	-6.280365000	-3.241279000
1	1.269458000	-6.911886000	-2.615179000
1	1.328766000	-5.985387000	-4.120863000
1	2.761506000	-6.868454000	-3.564516000
6	-5.192765000	-0.349717000	-4.319176000
1	-6.172624000	0.119882000	-4.414075000
1	-4.433473000	0.432103000	-4.270498000
1	-5.007187000	-0.953484000	-5.208275000
6	-2.049685000	3.658784000	-4.877128000
1	-2.338725000	3.139675000	-5.791972000
1	-2.149562000	4.732603000	-5.040516000
1	-1.003052000	3.432837000	-4.670456000

6	2.568239000	-1.466144000	-3.617938000
1	2.069696000	-2.181543000	-2.964143000
1	1.871308000	-1.197254000	-4.412720000
1	3.440718000	-1.943974000	-4.066648000
6	6.922958000	3.928283000	3.630771000
1	7.353058000	4.719754000	3.016569000
1	6.013995000	4.309033000	4.097814000
1	7.635383000	3.672669000	4.416399000
6	3.669603000	0.102126000	4.004020000
1	3.769545000	0.897538000	3.264919000
1	2.972428000	0.424680000	4.778180000
6	-4.931374000	2.684302000	4.667004000
1	-4.529753000	3.085409000	5.598731000
1	-5.994575000	2.921141000	4.617649000
1	-4.820510000	1.599091000	4.682379000
6	-3.988218000	-2.082881000	5.442649000
1	-2.977895000	-2.472235000	5.309367000
1	-3.988192000	-1.416145000	6.306165000
1	-4.657540000	-2.917975000	5.651770000
1	4.642173000	-0.071961000	4.466123000
6	0.377576000	5.024809000	3.237342000
1	0.787192000	4.015786000	3.367490000
1	1.209063000	5.736125000	3.311541000
6	1.874062000	3.883267000	-3.845255000
1	1.360018000	4.801743000	-4.151492000
1	1.114671000	3.105872000	-3.700473000
6	2.877625000	3.468643000	-4.894840000
1	3.390946000	2.553788000	-4.596649000

1 2.370953000 3.288198000 -5.843944000

1 3.625113000 4.248588000 -5.042512000

6 -0.664586000 5.316670000 4.291853000

1 -1.484719000 4.600352000 4.227726000

1 -0.222295000 5.248088000 5.286722000

1 -1.071762000 6.319887000 4.161854000

P6Q2 with one $AgOCOCF_3$

Sum of electronic and zero-point Energies= -3823.752236

Sum of electronic and thermal Energies= -3823.674025

Sum of electronic and thermal Enthalpies= -3823.673081

Sum of electronic and thermal Free Energies= -3823.875602

6 -5.951990000 0.767873000 0.342773000

6 -5.259811000 -0.561938000 0.519683000

6 -4.895564000 -0.996052000 1.785565000

6 -4.286592000 -2.227198000 1.979269000

6 -4.080281000 -3.072994000 0.893102000

6 -4.418097000 -2.631377000 -0.378020000

6 -4.980972000 -1.377374000 -0.576790000

6 -5.001056000 1.933155000 0.215291000

6 -4.539388000 2.324733000 -1.032082000

6 -3.689094000 3.412076000 -1.179491000

6 -3.321102000 4.156892000 -0.061440000

6 -3.759375000 3.750906000 1.190766000

6 -4.577338000 2.640181000 1.339828000

6 -2.496462000 5.412901000 -0.218411000

6 1.882279000 4.872076000 -0.259296000

6 1.219236000 4.934826000 0.895676000

6 -0.250886000 5.059486000 0.973227000

6 -1.018147000 5.164740000 -0.305633000

6 -0.360377000 5.053259000 -1.460310000

6 1.097906000 4.865536000 -1.535062000

6 5.851283000 -0.440687000 -0.251227000

6 4.935772000 -1.611048000 -0.003437000

6 4.770547000 -2.612704000 -0.935266000

6 3.912591000 -3.693643000 -0.731429000

6 3.183099000 -3.792060000 0.443647000

6 3.316079000 -2.764941000 1.408586000

6 4.214893000 -1.704794000 1.202440000

6 2.347343000 -5.015991000 0.734601000

6 -3.519671000 -4.460229000 1.100824000

6 -2.020704000 -4.500415000 1.041121000

6 -1.235237000 -4.265247000 2.094218000

6 0.230490000 -4.347537000 2.029108000

6 0.860593000 -4.789977000 0.748772000

6 0.084703000 -4.977425000 -0.317838000

6 -1.387369000 -4.841477000 -0.271802000

8 -3.887172000 -2.690417000 3.199857000

6 -3.980796000 -1.824389000 4.310409000

8 -5.307146000 -0.883768000 -1.799956000

6 -4.945197000 -1.621000000 -2.950947000

8 -5.027900000 2.193284000 2.547428000

6 -4.569226000 2.829434000 3.720014000

8 -3.195087000 3.830974000 -2.376010000

6 -3.463653000 3.058418000 -3.529792000

8 -0.807661000 5.116848000 2.049202000

8	4.484639000	-0.775308000	2.133548000	1	6.634253000	-0.429873000	0.510029000
6	3.604639000	-0.611865000	3.233950000	1	6.339985000	-0.593270000	-1.215337000
8	3.802322000	-4.665479000	-1.681709000	1	5.305323000	-2.553534000	-1.875124000
6	2.941681000	-4.322577000	-2.772860000	1	2.856472000	-2.920477000	2.376357000
8	0.913962000	-4.075796000	2.998631000	1	2.644500000	-5.407046000	1.709357000
8	-2.055745000	-5.058045000	-1.256297000	1	2.579702000	-5.765445000	-0.018810000
8	1.651228000	4.733512000	-2.607248000	1	-3.839299000	-4.834639000	2.072507000
6	3.382257000	4.833373000	-0.351010000	1	-3.914131000	-5.119218000	0.328604000
6	5.163285000	0.903741000	-0.246562000	1	-1.653564000	-4.004040000	3.058404000
6	5.478116000	1.851740000	0.715037000	1	0.487657000	-5.273790000	-1.278719000
6	4.884515000	3.107172000	0.724732000	1	-3.455096000	-0.886915000	4.093761000
6	3.973305000	3.443420000	-0.275132000	1	-5.031098000	-1.580664000	4.510003000
6	3.647842000	2.492119000	-1.230494000	1	-5.539503000	-2.540857000	-3.005583000
6	4.216257000	1.225990000	-1.217289000	1	-3.889803000	-1.901746000	-2.899590000
8	5.144701000	4.054369000	1.670880000	1	-3.473524000	2.824203000	3.744884000
6	6.165166000	3.811469000	2.615494000	1	-4.898784000	3.874984000	3.735458000
8	3.917814000	0.260377000	-2.123351000	1	-3.185388000	2.016404000	-3.348843000
6	2.891825000	0.526209000	-3.069236000	1	-4.536018000	3.089419000	-3.757007000
1	-6.574975000	0.727768000	-0.549610000	1	2.571078000	-0.570216000	2.865694000
1	-6.607075000	0.935106000	1.197409000	1	3.685719000	-1.468323000	3.910512000
1	-5.099647000	-0.336967000	2.617237000	1	2.028785000	-3.856458000	-2.385136000
1	-4.230353000	-3.295486000	-1.210413000	1	3.443821000	-3.586938000	-3.409407000
1	-4.857114000	1.746894000	-1.888467000	1	3.795850000	5.439570000	0.451301000
1	-3.440317000	4.327761000	2.047212000	1	3.677776000	5.282296000	-1.300862000
1	-2.686755000	6.063558000	0.635060000	1	6.192991000	1.579416000	1.478612000
1	-2.810077000	5.934220000	-1.122645000	1	2.953634000	2.773799000	-2.008350000
1	1.735907000	4.931190000	1.849038000	1	5.919423000	2.928442000	3.216953000
1	-0.878020000	5.102051000	-2.410006000	1	7.111101000	3.612357000	2.098132000

1	3.214401000	1.324211000	-3.746751000
1	1.991517000	0.860406000	-2.547110000
9	-1.530741000	-0.960009000	-3.656964000
9	-0.640370000	0.896899000	-3.036508000
9	-2.270199000	0.047726000	-1.900798000
8	0.365335000	-0.285995000	-0.804341000
8	0.026912000	-2.212438000	-1.895498000
6	-0.159004000	-1.022848000	-1.687400000
6	-1.163470000	-0.253374000	-2.588310000
47	1.764285000	-1.480110000	0.269891000
6	2.628294000	-5.578766000	-3.545232000
1	2.115359000	-6.305261000	-2.912737000
1	1.979768000	-5.339901000	-4.388766000
1	3.541242000	-6.038614000	-3.924929000
6	-5.197166000	-0.743571000	-4.153615000
1	-6.244812000	-0.444406000	-4.204159000
1	-4.579395000	0.154179000	-4.102192000
1	-4.943597000	-1.282114000	-5.067453000
6	-2.653882000	3.630568000	-4.668377000
1	-2.851530000	3.069239000	-5.582625000
1	-2.913606000	4.675692000	-4.842137000
1	-1.587384000	3.565151000	-4.450271000
6	2.616917000	-0.749393000	-3.824427000
1	2.262163000	-1.525065000	-3.146038000
1	1.841244000	-0.576645000	-4.571477000
1	3.515511000	-1.103420000	-4.332529000
6	6.282157000	5.033076000	3.495731000
1	6.536222000	5.911756000	2.902387000
1	5.341900000	5.225012000	4.013649000
1	7.063226000	4.881085000	4.241874000
6	3.971703000	0.675320000	3.930081000
1	3.857882000	1.519260000	3.249158000
1	3.319987000	0.828700000	4.790727000
6	-5.135421000	2.077562000	4.901961000
1	-4.811252000	2.544575000	5.832904000
1	-6.225511000	2.079813000	4.873761000
1	-4.792398000	1.041739000	4.898273000
6	-3.361079000	-2.522030000	5.498098000
1	-2.307902000	-2.738299000	5.313366000
1	-3.430979000	-1.886003000	6.381673000
1	-3.877160000	-3.459827000	5.706316000
1	5.004234000	0.641888000	4.280197000

P6Q3 with one $AgOCOCF_3$

Sum of electronic and zero-point Energies= -3665.409885

Sum of electronic and thermal Energies= -3665.338006

Sum of electronic and thermal Enthalpies= -3665.337062

Sum of electronic and thermal Free Energies= -3665.524423

6	0.216617000	4.756059000	-0.996218000
6	1.564966000	4.229609000	-0.605187000
6	2.255716000	3.362487000	-1.435455000
6	3.508819000	2.870910000	-1.085287000
6	4.076699000	3.241330000	0.133875000
6	3.382372000	4.105764000	0.969477000
6	2.144094000	4.611105000	0.607416000
6	-0.938941000	4.228736000	-0.195923000

6	-0.857450000	3.351535000	0.803442000	6	3.700301000	1.579888000	-3.096278000
6	-2.034779000	2.923419000	1.582818000	8	1.425636000	5.473768000	1.366631000
6	-3.382724000	3.400452000	1.144753000	6	1.794871000	5.663961000	2.718974000
6	-3.461113000	4.285863000	0.152659000	8	-2.395266000	5.555071000	-1.495709000
6	-2.278900000	4.762469000	-0.584337000	8	-1.906756000	2.240965000	2.576839000
6	-4.584355000	2.835274000	1.843141000	8	-5.808171000	2.401688000	-0.598613000
6	-5.399801000	-1.127457000	0.357950000	6	-6.139766000	2.290076000	-1.970799000
6	-5.755113000	-0.007631000	-0.378850000	8	1.511794000	-3.340109000	-2.863295000
6	-5.498823000	1.272572000	0.089207000	6	2.437208000	-2.473000000	-3.501387000
6	-4.889171000	1.446585000	1.331813000	8	1.908198000	-4.288925000	2.541746000
6	-4.528042000	0.327894000	2.064461000	6	0.797742000	-4.836406000	3.232683000
6	-4.769065000	-0.953244000	1.588003000	8	5.878324000	-1.997842000	-0.587848000
6	-0.496889000	-4.907550000	-1.727706000	8	4.204845000	1.557799000	2.913044000
6	0.651611000	-4.337672000	-0.922157000	8	-4.427307000	-2.082164000	2.267538000
6	0.742156000	-4.574851000	0.440450000	6	-3.451494000	-1.979450000	3.288145000
6	1.809332000	-4.110754000	1.198985000	6	-5.670465000	-2.514146000	-0.177623000
6	2.858015000	-3.447752000	0.564981000	6	-1.829156000	-4.290082000	-1.406866000
6	2.768126000	-3.199745000	-0.805695000	6	-2.378208000	-3.309976000	-2.125590000
6	1.659772000	-3.599054000	-1.546509000	6	-3.711992000	-2.744659000	-1.821682000
6	4.081599000	-3.021778000	1.347332000	6	-4.403246000	-3.206964000	-0.579409000
6	5.409677000	2.670648000	0.563079000	6	-3.867819000	-4.208021000	0.117652000
6	5.246022000	1.204668000	0.815245000	6	-2.589283000	-4.836680000	-0.240396000
6	5.721957000	0.263534000	-0.001824000	8	-4.239001000	-1.960161000	-2.578340000
6	5.391111000	-1.158267000	0.135687000	8	-2.162628000	-5.773102000	0.407016000
6	4.389995000	-1.556291000	1.194392000	1	0.198902000	5.845831000	-0.910725000
6	3.935255000	-0.604656000	2.050458000	1	0.021937000	4.543529000	-2.048936000
6	4.430894000	0.799893000	2.000572000	1	1.793785000	3.065775000	-2.366376000
8	4.242445000	2.038287000	-1.865937000	1	3.828501000	4.369452000	1.917812000

1	0.084512000	2.923525000	1.122747000	1	-6.325658000	-2.434967000	-1.045509000
1	-4.412604000	4.675743000	-0.185311000	1	-6.174138000	-3.116548000	0.579307000
1	-5.437449000	3.491870000	1.673525000	1	-1.884349000	-2.895055000	-2.996090000
1	-4.388723000	2.786081000	2.914061000	1	-4.342865000	-4.594020000	1.010699000
1	-6.213476000	-0.158518000	-1.345534000	8	0.146559000	0.195418000	0.198794000
1	-4.026482000	0.479265000	3.009560000	6	0.209387000	0.251919000	-1.041548000
1	-0.290522000	-4.770359000	-2.786039000	47	2.293150000	-0.616122000	0.330321000
1	-0.557287000	-5.978640000	-1.527084000	8	1.201635000	-0.030133000	-1.737879000
1	-0.044512000	-5.154099000	0.900357000	6	-1.049045000	0.713193000	-1.819111000
1	3.610920000	-2.740884000	-1.304220000	9	-1.359118000	-0.169754000	-2.772269000
1	4.946616000	-3.589002000	1.000891000	9	-2.113010000	0.863146000	-1.040325000
1	3.923409000	-3.244303000	2.399799000	9	-0.812304000	1.888489000	-2.422801000
1	6.162847000	2.820210000	-0.210953000	6	1.095196000	-4.778159000	4.711145000
1	5.738700000	3.170880000	1.473738000	1	1.239563000	-3.747192000	5.036303000
1	6.332087000	0.517405000	-0.858724000	1	0.261270000	-5.203246000	5.271062000
1	3.355892000	-0.864255000	2.930588000	1	1.994587000	-5.347341000	4.947943000
1	2.714622000	1.140353000	-2.926528000	6	0.699739000	6.461064000	3.386086000
1	3.596748000	2.425084000	-3.786020000	1	0.569460000	7.424368000	2.892205000
1	2.751684000	6.196308000	2.774855000	1	-0.245058000	5.918484000	3.344508000
1	1.919335000	4.690272000	3.206032000	1	0.953023000	6.638645000	4.432091000
1	-5.394243000	1.669282000	-2.478835000	6	-3.007582000	-3.377805000	3.650135000
1	-7.116481000	1.804398000	-2.079732000	1	-2.268988000	-3.335275000	4.452536000
1	2.541905000	-1.559056000	-2.911507000	1	-3.852359000	-3.976567000	3.992917000
1	3.413862000	-2.965769000	-3.573462000	1	-2.559007000	-3.878145000	2.790530000
1	-0.103433000	-4.261313000	2.994930000	6	1.889678000	-2.147145000	-4.868900000
1	0.631264000	-5.869748000	2.910859000	1	0.941603000	-1.616983000	-4.777778000
1	-2.606798000	-1.381449000	2.929935000	1	2.592650000	-1.507698000	-5.404502000
1	-3.874844000	-1.474115000	4.163606000	6	4.652106000	0.546486000	-3.650047000

1 4.735593000 -0.305724000 -2.972831000

1 4.285026000 0.183363000 -4.610734000

1 5.647057000 0.967875000 -3.799629000

6 -6.169011000 3.681724000 -2.556939000

1 -5.186759000 4.151451000 -2.494323000

1 -6.459947000 3.634532000 -3.607193000

1 -6.889821000 4.307096000 -2.028476000

1 1.735008000 -3.054015000 -5.454556000

MeP5 with two $AgOCOCF_3$

Sum of electronic and zero-point Energies= -3843.072873

Sum of electronic and thermal Energies= -3843.001856

Sum of electronic and thermal Enthalpies= -3843.000912

Sum of electronic and thermal Free Energies= -3843.183185

9 -0.111426000 4.380197000 0.872660000

9 -0.535997000 3.544452000 2.815697000

9 1.498551000 3.751206000 2.154214000

9 -1.527192000 -4.226518000 -2.783822000

9 -0.087574000 -5.058583000 -1.413321000

9 0.574806000 -4.083906000 -3.222038000

8 1.254327000 1.464116000 0.898280000

8 0.919325000 -2.387006000 -1.115787000

8 -1.305863000 -2.084996000 -1.294865000

6 0.173326000 2.032625000 1.103719000

6 0.267834000 3.443860000 1.749873000

6 -0.234828000 -2.684102000 -1.465541000

6 -0.329006000 -4.031875000 -2.239317000

47 1.443086000 -0.494377000 -0.083661000

47 -1.475835000 -0.176842000 -0.192159000

8 2.164538000 4.173906000 -1.952628000

8 -1.017074000 0.209429000 -4.067471000

8 4.816269000 1.597571000 1.789403000

8 3.688183000 -0.882850000 -2.997275000

8 0.688728000 0.035940000 4.457206000

8 3.321124000 -3.568103000 1.234465000

8 -3.979111000 1.197324000 3.163120000

8 -1.816342000 -3.673460000 1.776740000

8 -2.993223000 3.857927000 -0.936503000

8 -4.759884000 -1.328219000 -1.487915000

6 -2.312506000 2.558285000 -3.302500000

1 -2.607129000 2.151213000 -4.270616000

1 -2.514326000 3.628455000 -3.318823000

6 -0.825332000 2.351278000 -3.120519000

6 -0.030251000 3.347689000 -2.572326000

1 -0.520388000 4.244730000 -2.220167000

6 1.351319000 3.214335000 -2.480470000

6 1.969265000 2.062727000 -2.960572000

6 1.171047000 1.046752000 -3.470949000

1 1.667272000 0.159373000 -3.834862000

6 -0.207682000 1.174346000 -3.547278000

6 1.653131000 5.478701000 -1.819445000

1 0.884602000 5.538804000 -1.045936000

1 1.240135000 5.839648000 -2.766872000

6 -0.420594000 -0.976150000 -4.528328000

1 0.198740000 -1.441457000 -3.756950000

1 0.200239000 -0.795717000 -5.412025000

6	3.472783000	1.902602000	-2.942454000
1	3.786843000	1.408558000	-3.861536000
1	3.939801000	2.885150000	-2.911783000
6	3.924786000	1.097536000	-1.751137000
6	4.241625000	1.731666000	-0.560586000
1	4.192182000	2.811004000	-0.541895000
6	4.538470000	1.015132000	0.592663000
6	4.514232000	-0.376784000	0.563836000
6	4.230599000	-1.017770000	-0.640468000
1	4.225037000	-2.098435000	-0.643841000
6	3.951440000	-0.299156000	-1.797615000
6	4.858040000	3.003182000	1.847617000
1	3.894918000	3.442195000	1.578661000
1	5.637418000	3.405421000	1.192201000
6	4.669264000	-1.195019000	1.822796000
1	5.241396000	-0.635504000	2.560306000
1	5.209028000	-2.113721000	1.599231000
6	3.305846000	-1.524637000	2.385724000
6	2.651356000	-0.606087000	3.195741000
1	3.166280000	0.316947000	3.419688000
6	1.370895000	-0.843539000	3.672576000
6	0.721860000	-2.038132000	3.360056000
6	1.369728000	-2.949466000	2.537579000
1	0.846883000	-3.861790000	2.288698000
6	2.646611000	-2.705515000	2.043860000
6	1.321560000	1.244855000	4.799395000
1	1.608972000	1.814015000	3.911555000
1	2.210783000	1.070407000	5.414044000

6	2.658955000	-4.735748000	0.810139000
1	1.743793000	-4.493903000	0.266210000
1	2.423635000	-5.391993000	1.654175000
6	-0.637430000	-2.355610000	3.936343000
1	-0.680955000	-1.978328000	4.958324000
1	-0.748282000	-3.438863000	3.980417000
6	-1.798865000	-1.777411000	3.165119000
6	-2.340125000	-0.550447000	3.522547000
1	-1.872480000	-0.018727000	4.337794000
6	-3.430727000	-0.009697000	2.855211000
6	-4.018143000	-0.714000000	1.805922000
6	-3.476362000	-1.942761000	1.444661000
1	-3.932887000	-2.467124000	0.617994000
6	-2.375254000	-2.475925000	2.104277000
6	-3.365982000	1.965225000	4.169118000
1	-2.321109000	2.174218000	3.927227000
1	-3.421886000	1.468718000	5.143452000
6	-2.498522000	-4.486981000	0.850402000
1	-2.567277000	-4.008469000	-0.130079000
1	-3.505663000	-4.729349000	1.204557000
6	-5.151548000	-0.120603000	0.999951000
1	-5.814971000	-0.914681000	0.661494000
1	-5.727253000	0.566180000	1.617552000
6	-4.572897000	0.617321000	-0.183235000
6	-4.163090000	1.936083000	-0.047063000
1	-4.354670000	2.421440000	0.898141000
6	-3.452480000	2.584263000	-1.048285000
6	-3.149448000	1.908497000	-2.229001000

6	-3.604478000	0.604398000	-2.386609000
1	-3.362351000	0.096909000	-3.307661000
6	-4.310893000	-0.047414000	-1.383146000
6	-3.232492000	4.537407000	0.272372000
1	-2.795859000	4.002782000	1.119915000
1	-4.304677000	4.679875000	0.443585000
6	-4.408505000	-2.062202000	-2.638469000
1	-3.323134000	-2.134124000	-2.741972000
1	-4.841902000	-1.620550000	-3.541524000
8	-0.992107000	1.675481000	0.869267000
6	3.692020000	-2.292029000	-3.067560000
1	4.678201000	-2.697917000	-2.820942000
1	2.937241000	-2.724667000	-2.406290000
1	-4.821881000	-3.058910000	-2.498334000
1	-1.916496000	-5.401271000	0.761886000
1	-1.234109000	-1.649812000	-4.789530000
1	3.452243000	-2.541966000	-4.098879000
1	3.348078000	-5.249625000	0.143086000
1	2.493678000	6.106924000	-1.530815000
1	-2.752839000	5.508738000	0.176113000
1	-3.918454000	2.901550000	4.216162000
1	0.595901000	1.817825000	5.372804000
1	5.093961000	3.256774000	2.879036000

EtP5 with two AgOCOCF$_3$

Sum of electronic and zero-point Energies= -4236.016655

Sum of electronic and thermal Energies= -4235.931715

Sum of electronic and thermal Enthalpies= -4235.930771

Sum of electronic and thermal Free Energies= -4236.143694

9	4.263834000	0.505087000	-0.958404000
9	4.197839000	1.540619000	0.929962000
9	4.447186000	-0.590288000	0.885016000
9	-5.210988000	0.574830000	-0.913148000
9	-5.172030000	-0.949181000	0.604429000
9	-4.845318000	-1.475860000	-1.459975000
8	1.872225000	-0.916844000	0.349697000
8	-2.449743000	-1.405587000	-0.118449000
8	-2.658103000	0.819905000	-0.386963000
6	2.267944000	0.255198000	0.286981000
6	3.814232000	0.427016000	0.300504000
6	-3.065772000	-0.348660000	-0.329356000
6	-4.598019000	-0.543429000	-0.535168000
47	-0.246405000	-1.510388000	0.184313000
47	-0.538420000	1.401991000	-0.083140000
8	3.311621000	-1.764068000	-3.298786000
8	-1.681317000	0.444044000	-3.998988000
8	2.920902000	-4.185355000	1.276781000
8	-1.348236000	-4.172852000	-2.197111000
8	1.979537000	-0.154496000	4.221822000
8	-2.176068000	-3.431064000	2.714086000
8	1.670724000	4.450743000	2.113772000
8	-2.868401000	1.538856000	3.205063000
8	2.502544000	3.302548000	-2.721440000
8	-2.644456000	4.257065000	-1.022504000
6	0.476610000	2.179921000	-4.245884000
1	-0.348236000	2.289045000	-4.950213000

1	1.381827000	2.536205000	-4.735752000	1	4.561674000	-4.650322000	0.117994000
6	0.648568000	0.714266000	-3.915737000	6	0.393670000	-4.466787000	2.414625000
6	1.910007000	0.166306000	-3.734484000	1	1.274218000	-4.896071000	2.889219000
1	2.759455000	0.832692000	-3.789140000	1	-0.452997000	-5.121324000	2.615615000
6	2.089310000	-1.192257000	-3.495801000	6	0.128178000	-3.097932000	2.999570000
6	0.985646000	-2.040299000	-3.456208000	6	1.185897000	-2.281022000	3.377013000
6	-0.281194000	-1.486996000	-3.598806000	1	2.185718000	-2.669130000	3.247936000
1	-1.125884000	-2.158418000	-3.555191000	6	0.973261000	-1.000106000	3.866650000
6	-0.459340000	-0.129251000	-3.820126000	6	-0.327136000	-0.522198000	4.024417000
6	4.460730000	-1.089136000	-3.769503000	6	-1.384446000	-1.334547000	3.639754000
1	4.586034000	-0.135749000	-3.250310000	1	-2.384877000	-0.945278000	3.760054000
1	4.348180000	-0.877624000	-4.839393000	6	-1.172412000	-2.605738000	3.117604000
6	-2.819748000	-0.388315000	-3.974662000	6	3.311525000	-0.610203000	4.109381000
1	-2.870189000	-0.922007000	-3.019346000	1	3.505013000	-0.944910000	3.086281000
1	-2.748171000	-1.142176000	-4.768111000	1	3.469976000	-1.464886000	4.778307000
6	1.141752000	-3.533100000	-3.269136000	6	-3.499176000	-2.934115000	2.718618000
1	0.407766000	-4.046022000	-3.889817000	1	-3.531660000	-1.974764000	2.197112000
1	2.134028000	-3.834830000	-3.599558000	1	-3.833651000	-2.777301000	3.751215000
6	0.955661000	-3.926505000	-1.826179000	6	-0.584483000	0.841791000	4.619978000
6	2.043442000	-3.967445000	-0.969128000	1	0.185033000	1.055856000	5.361069000
1	3.017564000	-3.761919000	-1.388927000	1	-1.544860000	0.820512000	5.134765000
6	1.888936000	-4.185463000	0.394419000	6	-0.596433000	1.954707000	3.600702000
6	0.613455000	-4.370897000	0.923782000	6	0.552115000	2.687396000	3.341846000
6	-0.479366000	-4.371523000	0.057865000	1	1.448965000	2.417075000	3.880199000
1	-1.459935000	-4.525569000	0.485704000	6	0.563857000	3.720519000	2.413108000
6	-0.320462000	-4.165778000	-1.309219000	6	-0.603078000	4.047330000	1.725508000
6	4.213706000	-3.866446000	0.801362000	6	-1.757370000	3.312027000	1.981923000
1	4.180125000	-2.922220000	0.252550000	1	-2.649370000	3.567646000	1.427794000

6	-1.764681000	2.275069000	2.907942000
6	2.908278000	4.049011000	2.662451000
1	3.065253000	2.985891000	2.464658000
1	2.903147000	4.198885000	3.748855000
6	-4.105118000	1.889901000	2.617147000
1	-4.049480000	1.759832000	1.530999000
1	-4.328019000	2.944069000	2.819719000
6	-0.601559000	5.115167000	0.655133000
1	-1.565130000	5.621617000	0.644257000
1	0.168811000	5.852629000	0.871962000
6	-0.334279000	4.481700000	-0.689395000
6	0.969284000	4.271901000	-1.118306000
1	1.766972000	4.632676000	-0.485870000
6	1.244358000	3.554686000	-2.274551000
6	0.197303000	3.030720000	-3.031568000
6	-1.109201000	3.273953000	-2.624890000
1	-1.908176000	2.859863000	-3.222097000
6	-1.386697000	3.995392000	-1.468491000
6	3.594609000	3.726051000	-1.931926000
1	3.534173000	3.257685000	-0.944389000
1	3.559104000	4.813787000	-1.796346000
6	-3.739235000	3.655130000	-1.686687000
1	-3.584765000	2.574501000	-1.730128000
1	-3.813888000	4.043359000	-2.709360000
8	1.642418000	1.320501000	0.172554000
6	-2.668849000	-4.343154000	-1.714763000
1	-2.760000000	-5.317718000	-1.220779000
1	-2.896592000	-3.559405000	-0.986885000

6	-4.374425000	-3.940803000	2.013243000
1	-4.321698000	-4.913586000	2.503462000
1	-5.410778000	-3.601804000	2.020204000
1	-4.059375000	-4.052527000	0.974983000
6	-3.601142000	-4.255754000	-2.898827000
1	-3.372853000	-5.029792000	-3.632204000
1	-4.632868000	-4.380020000	-2.568247000
1	-3.514609000	-3.281699000	-3.382274000
6	-5.163402000	0.998048000	3.221071000
1	-5.247518000	1.173378000	4.294016000
1	-6.129921000	1.196955000	2.756781000
1	-4.917601000	-0.051568000	3.058760000
6	-4.986035000	3.975174000	-0.898601000
1	-5.857065000	3.538719000	-1.388579000
1	-4.917164000	3.558116000	0.107208000
1	-5.132451000	5.053027000	-0.820661000
6	-4.038528000	0.479216000	-4.172104000
1	-3.990571000	0.997098000	-5.130552000
1	-4.939390000	-0.134710000	-4.150937000
1	-4.113827000	1.221678000	-3.377934000
6	4.226462000	0.534018000	4.474029000
1	4.080253000	1.372848000	3.792764000
1	4.034955000	0.873772000	5.492462000
1	5.267068000	0.214855000	4.403479000
6	5.127672000	-3.753800000	1.997714000
1	4.786377000	-2.960152000	2.663887000
1	5.153991000	-4.689720000	2.556754000
1	6.140628000	-3.514638000	1.671380000

6	5.652747000	-1.978604000	-3.507858000
1	5.756089000	-2.167933000	-2.438441000
1	5.543576000	-2.934319000	-4.021624000
1	6.564753000	-1.495880000	-3.861680000
6	4.863659000	3.327777000	-2.646048000
1	5.731296000	3.618556000	-2.052248000
1	4.897061000	2.248597000	-2.794287000
1	4.928484000	3.815982000	-3.619033000
6	3.993501000	4.873627000	2.013348000
1	4.010753000	4.700285000	0.936546000
1	3.833136000	5.937269000	2.192542000
1	4.966287000	4.595136000	2.420851000

EtP6 with two $AgOCOCF_3$

Sum of electronic and zero-point Energies= -4813.851519

Sum of electronic and thermal Energies= -4813.752279

Sum of electronic and thermal Enthalpies= -4813.751334

Sum of electronic and thermal Free Energies= -4813.997205

6	-2.623461000	4.898085000	-0.090942000
6	-3.404746000	3.611263000	-0.082158000
6	-3.713279000	2.993186000	1.125105000
6	-4.454831000	1.821078000	1.167679000
6	-4.928106000	1.260897000	-0.019607000
6	-4.597069000	1.863389000	-1.224250000
6	-3.829543000	3.019832000	-1.270894000
6	-1.129864000	4.686388000	-0.084786000
6	-0.444528000	4.502884000	-1.281977000
6	0.940939000	4.432024000	-1.317157000
6	1.671803000	4.601394000	-0.139789000
6	0.984264000	4.703654000	1.060009000
6	-0.404514000	4.721574000	1.104387000
6	3.171756000	4.762250000	-0.180398000
6	5.631897000	1.227156000	-0.061843000
6	5.155049000	1.800202000	1.108613000
6	4.317364000	2.904840000	1.083111000
6	3.975756000	3.488325000	-0.135164000
6	4.437165000	2.904041000	-1.305113000
6	5.244025000	1.774913000	-1.281018000
6	3.151854000	-4.772657000	0.180381000
6	1.652494000	-4.606606000	0.138938000
6	0.965147000	-4.707371000	-1.061092000
6	-0.423655000	-4.720791000	-1.106131000
6	-1.149461000	-4.682501000	0.082641000
6	-0.464072000	-4.500515000	1.280062000
6	0.921635000	-4.434191000	1.315874000
6	-2.643789000	-4.889035000	0.088157000
6	-5.770048000	0.011028000	-0.001975000
6	-4.933540000	-1.242483000	0.015955000
6	-4.605417000	-1.846294000	1.220731000

6	-3.842905000	-3.006039000	1.267646000	6	5.087732000	1.460220000	-3.637847000
6	-3.420324000	-3.599357000	0.079051000	6	6.489982000	-0.012115000	0.001069000
6	-3.725953000	-2.980104000	-1.128297000	6	3.960643000	-3.501736000	0.135781000
6	-4.462430000	-1.804840000	-1.171144000	6	4.424142000	-2.919646000	1.305997000
8	-4.766989000	1.175913000	2.323491000	6	5.235744000	-1.793903000	1.282393000
6	-4.228533000	1.677263000	3.531485000	6	5.626418000	-1.247608000	0.063470000
8	-3.473195000	3.642754000	-2.425548000	6	5.147432000	-1.818324000	-1.107258000
6	-3.586543000	2.919529000	-3.635230000	6	4.304941000	-2.919309000	-1.082248000
8	-1.120971000	4.834670000	2.256666000	8	5.705501000	-1.183524000	2.410501000
6	-0.479802000	4.504496000	3.473559000	6	5.079278000	-1.478336000	3.639069000
8	1.650856000	4.253697000	-2.462138000	8	3.793011000	-3.500527000	-2.204788000
6	0.938233000	3.950016000	-3.645689000	6	3.986156000	-2.850075000	-3.441364000
8	3.807826000	3.488645000	2.205401000	1	-2.889480000	5.473373000	-0.976416000
6	3.997930000	2.837739000	3.442201000	1	-2.902360000	5.484507000	0.783064000
8	1.631545000	-4.257605000	2.461101000	1	-3.350715000	3.461332000	2.028852000
6	0.919207000	-3.951526000	3.644238000	1	-4.950608000	1.397806000	-2.132727000
8	-1.139856000	-4.832217000	-2.258721000	1	-1.030463000	4.447948000	-2.188213000
6	-0.497294000	-4.503398000	-3.475254000	1	1.566383000	4.795889000	1.966120000
8	-3.489582000	-3.630358000	2.422497000	1	3.465444000	5.390323000	0.661070000
6	-3.600924000	-2.906677000	3.632095000	1	3.433737000	5.298811000	-1.092601000
8	-4.771578000	-1.158416000	-2.327092000	1	5.448682000	1.343361000	2.042764000
6	-4.234490000	-1.661806000	-3.534812000	1	4.145156000	3.358352000	-2.241590000
8	5.711611000	1.162333000	-2.408828000	1	3.411409000	-5.310302000	1.092646000

1	3.443783000	-5.401661000	-0.661010000	1	-3.111919000	-1.935213000	3.512092000
1	1.547332000	-4.802023000	-1.966917000	1	-4.656550000	-2.737792000	3.875206000
1	-1.050225000	-4.443302000	2.186013000	1	-4.626273000	-2.668004000	-3.725673000
1	-2.912218000	-5.463425000	0.973495000	1	-3.145252000	-1.735517000	-3.449927000
1	-2.924343000	-5.474414000	-0.786010000	1	4.004701000	1.319626000	-3.539995000
1	-6.412019000	0.019215000	0.878409000	1	5.265658000	2.505304000	-3.916888000
1	-6.411757000	0.005637000	-0.882570000	1	7.129981000	0.036558000	0.880602000
1	-4.957187000	-1.379145000	2.129082000	1	7.130274000	-0.063586000	-0.878097000
1	-3.365002000	-3.449744000	-2.031915000	1	4.130025000	-3.372948000	2.242308000
1	-3.138989000	1.746770000	3.447082000	1	5.443353000	-1.362555000	-2.041204000
1	-4.616558000	2.684955000	3.722130000	1	3.996964000	-1.333009000	3.540259000
1	-4.642653000	2.754930000	-3.879169000	1	5.252358000	-2.524115000	3.918550000
1	-3.101577000	1.946071000	-3.514939000	1	5.052282000	-2.842382000	-3.699575000
1	-0.004957000	3.523071000	3.372803000	1	3.649810000	-1.809096000	-3.369627000
1	0.294363000	5.244245000	3.707529000	9	-0.925956000	0.950926000	-4.824522000
1	0.266467000	3.104246000	-3.463593000	9	-0.820411000	-1.195452000	-4.769975000
1	0.329436000	4.812406000	-3.944144000	9	0.921725000	-0.021158000	-4.274284000
1	3.656132000	1.798483000	3.370992000	8	-0.945982000	-1.072790000	2.176562000
1	5.064062000	2.824559000	3.700156000	8	-0.865470000	-1.181748000	-2.118355000
1	0.249706000	-3.104135000	3.461361000	8	-0.940419000	1.074746000	-2.173232000
1	0.308083000	-4.812236000	3.942791000	6	-0.797960000	0.064074000	2.652417000
1	-0.019708000	-3.523378000	-3.373848000	6	-0.795498000	-0.062586000	-2.648842000
1	0.274889000	-5.245204000	-3.709235000	6	-0.412310000	-0.083828000	-4.157651000

47	-1.165212000	-1.570836000	0.036132000	6	5.657587000	0.536221000	-4.688182000
47	-1.160881000	1.574018000	-0.033079000	1	5.195413000	0.741157000	-5.654946000
8	-0.863611000	1.183413000	2.121811000	1	6.734714000	0.677487000	-4.781469000
6	-0.416495000	0.083745000	4.161699000	1	5.468118000	-0.506314000	-4.429687000
9	-0.936826000	-0.947857000	4.828334000	6	3.192353000	-3.591037000	-4.490664000
9	0.917020000	0.013496000	4.279930000	1	2.128148000	-3.560174000	-4.256736000
9	-0.818956000	1.197853000	4.773161000	1	3.342199000	-3.128016000	-5.466958000
6	-1.545323000	-4.479143000	-4.561245000	1	3.508098000	-4.633391000	-4.549030000
1	-2.300319000	-3.722620000	-4.346335000	6	5.652304000	-0.556555000	4.689625000
1	-1.082529000	-4.236278000	-5.518483000	1	6.728725000	-0.702508000	4.783837000
1	-2.035965000	-5.449331000	-4.647366000	1	5.467611000	0.486729000	4.430683000
6	-4.622581000	-0.723480000	-4.651302000	1	5.188440000	-0.759202000	5.656061000
1	-5.707167000	-0.643520000	-4.731756000	6	1.921531000	-3.618358000	4.722257000
1	-4.212397000	0.271630000	-4.479349000	1	2.509014000	-2.745272000	4.439435000
1	-4.229690000	-1.095016000	-5.598450000	1	1.400960000	-3.393095000	5.653939000
6	-2.910221000	3.717747000	-4.722549000	6	-1.528315000	4.483585000	4.559148000
1	-3.368297000	4.702210000	-4.823192000	1	-1.066573000	4.239730000	5.516640000
1	-1.850774000	3.844304000	-4.499132000				
1	-2.997234000	3.193721000	-5.675118000	1	-2.016264000	5.455176000	4.644760000
6	1.940170000	3.614765000	-4.723441000	1	-2.285314000	3.729074000	4.344210000
1	1.419435000	3.391299000	-5.655464000	6	-4.620747000	0.740436000	4.647791000
1	2.619902000	4.450260000	-4.895921000	1	-4.214177000	-0.256208000	4.476126000
1	2.525311000	2.740045000	-4.440791000	1	-4.226980000	1.110544000	5.595136000

1	-5.705666000	0.664526000	4.727660000
6	-2.928638000	-3.707542000	4.719964000
1	-1.869475000	-3.838089000	4.497509000
1	-3.014501000	-3.183238000	5.672483000
1	-3.390554000	-4.690259000	4.820137000
6	3.208406000	3.583437000	4.491378000
1	2.144051000	3.558935000	4.257363000
1	3.355433000	3.119616000	5.467721000
1	3.530278000	4.623914000	4.549679000
1	2.599037000	-4.455510000	4.895452000

P6Q1 with two $AgOCOCF_3$

Sum of electronic and zero-point Energies= -4655.496526

Sum of electronic and thermal Energies= -4655.402736

Sum of electronic and thermal Enthalpies= -4655.401792

Sum of electronic and thermal Free Energies= -4655.638469

6	-3.589361000	-4.121032000	0.560609000
6	-4.128113000	-2.745867000	0.267208000
6	-4.177285000	-2.285812000	-1.045362000
6	-4.715240000	-1.043548000	-1.351433000
6	-5.273598000	-0.268928000	-0.334750000
6	-5.188287000	-0.708070000	0.977181000
6	-4.592809000	-1.921619000	1.293305000
6	-2.084811000	-4.172803000	0.659236000
6	-1.452524000	-3.885101000	1.867790000
6	-0.084897000	-4.068253000	2.028160000
6	0.669047000	-4.597179000	0.977940000
6	0.050755000	-4.806690000	-0.245085000
6	-1.307064000	-4.574536000	-0.425539000
6	2.107320000	-5.003238000	1.187773000
6	5.135366000	-1.991661000	0.609414000
6	4.619205000	-2.695206000	-0.468685000
6	3.615009000	-3.637564000	-0.303706000
6	3.127685000	-3.914920000	0.971627000
6	3.633771000	-3.200745000	2.048109000
6	4.619240000	-2.238297000	1.878708000
6	3.737691000	4.221942000	-0.737130000
6	2.231853000	4.312799000	-0.766094000
6	1.525905000	4.652238000	0.378633000
6	0.159123000	4.899217000	0.341401000
6	-0.507897000	4.866063000	-0.881925000
6	0.178338000	4.445555000	-2.015409000
6	1.530990000	4.136424000	-1.961054000
6	-1.934005000	5.351582000	-0.980972000
6	-5.938328000	1.044723000	-0.664284000
6	-4.920433000	2.134494000	-0.803197000

6	-4.440460000	2.541746000	-1.978550000	6	6.186208000	-0.932799000	0.385908000
6	-3.414837000	3.589782000	-2.095576000	6	4.307186000	2.858371000	-0.442107000
6	-2.933790000	4.246411000	-0.839519000	6	4.664961000	2.006927000	-1.476929000
6	-3.391713000	3.818309000	0.339224000	6	5.263034000	0.779089000	-1.229091000
6	-4.406739000	2.758066000	0.455835000	6	5.544291000	0.398031000	0.080407000
8	-4.749151000	-0.525291000	-2.604892000	6	5.166090000	1.240094000	1.116329000
6	-4.016889000	-1.180969000	-3.626260000	6	4.533541000	2.449311000	0.870004000
8	-4.443799000	-2.379794000	2.561377000	8	5.621560000	-0.094317000	-2.214263000
6	-4.600563000	-1.462929000	3.631492000	6	5.134277000	0.131337000	-3.518958000
8	-1.954432000	-4.768035000	-1.606126000	8	4.126921000	3.300773000	1.855693000
6	-1.178429000	-4.791975000	-2.790084000	6	4.197768000	2.860531000	3.193474000
8	0.571924000	-3.804012000	3.186187000	1	-4.010620000	-4.476481000	1.499387000
6	-0.147704000	-3.178562000	4.232950000	1	-3.914429000	-4.798270000	-0.227642000
8	3.073610000	-4.346172000	-1.335508000	1	-3.791687000	-2.935516000	-1.818739000
6	3.445902000	-3.997534000	-2.651372000	1	-5.584685000	-0.068635000	1.752554000
8	2.245214000	3.712173000	-3.034041000	1	-2.073098000	-3.554833000	2.689084000
6	1.540003000	3.362046000	-4.212331000	1	0.658347000	-5.179140000	-1.057523000
8	-0.579308000	5.239750000	1.429600000	1	2.326718000	-5.829785000	0.511314000
6	-0.035476000	4.987986000	2.713028000	1	2.214526000	-5.378146000	2.205739000
8	-2.976247000	3.912371000	-3.179290000	1	5.018982000	-2.470584000	-1.446870000
8	-4.833636000	2.423803000	1.540553000	1	3.232388000	-3.418669000	3.027851000
8	5.143589000	-1.507800000	2.905984000	1	4.119806000	4.551178000	-1.703152000
6	4.495103000	-1.543738000	4.156665000	1	4.102513000	4.923237000	0.013567000

1	2.079969000	4.748341000	1.301729000	1	4.540864000	-2.552375000	4.583823000
1	-0.368329000	4.395198000	-2.946671000	1	6.827515000	-1.231501000	-0.441901000
1	-2.074538000	5.828567000	-1.949965000	1	6.808224000	-0.836435000	1.274575000
1	-2.112558000	6.089596000	-0.200989000	1	4.461926000	2.333698000	-2.487219000
1	-6.494034000	0.953530000	-1.596659000	1	5.371590000	0.912524000	2.125302000
1	-6.634007000	1.302519000	0.133558000	1	4.043511000	0.232857000	-3.489627000
1	-4.779479000	2.105794000	-2.909477000	1	5.547360000	1.061819000	-3.925665000
1	-3.047702000	4.252703000	1.269731000	1	5.243996000	2.707409000	3.485233000
1	-3.025269000	-1.451432000	-3.251370000	1	3.677069000	1.900765000	3.296103000
1	-4.532520000	-2.105135000	-3.912893000	9	-1.431307000	0.085014000	4.815859000
1	-5.659586000	-1.209708000	3.753270000	9	-2.497597000	1.884777000	4.303370000
1	-4.052011000	-0.543880000	3.406702000	9	-0.338966000	1.896673000	4.426678000
1	-0.495560000	-3.936667000	-2.785905000	8	-0.395156000	0.887032000	-2.291195000
1	-0.585337000	-5.712622000	-2.833331000	8	-1.051471000	1.808062000	1.814229000
1	-0.639447000	-2.276358000	3.854975000	8	-1.556255000	-0.351258000	2.235393000
1	-0.922468000	-3.858260000	4.608078000	6	-0.463527000	-0.321313000	-2.581252000
1	3.254101000	-2.931653000	-2.819459000	6	-1.324268000	0.827649000	2.532723000
1	4.518169000	-4.174870000	-2.798249000	6	-1.396724000	1.173376000	4.048313000
1	0.726803000	2.674698000	-3.961272000	47	-0.743855000	1.652682000	-0.310995000
1	1.104476000	4.259867000	-4.665908000	47	-1.525868000	-1.326296000	0.219258000
1	0.350988000	3.964721000	2.744017000	8	-0.830362000	-1.295210000	-1.909920000
1	0.791280000	5.680316000	2.910554000	6	0.037824000	-0.656308000	-4.016154000
1	3.437735000	-1.283198000	4.028402000	9	-0.234887000	0.314511000	-4.884126000

9	1.368486000	-0.823175000	-3.990324000
9	-0.500353000	-1.781387000	-4.487580000
6	-1.138584000	5.168460000	3.726491000
1	-1.942033000	4.454213000	3.546433000
1	-0.750491000	4.995708000	4.730972000
1	-1.545969000	6.179204000	3.680531000
6	-4.056964000	-2.116315000	4.878346000
1	-4.585851000	-3.045753000	5.092310000
1	-2.995217000	-2.333664000	4.757815000
1	-4.172778000	-1.444653000	5.729566000
6	0.829245000	-2.834385000	5.330493000
1	0.300990000	-2.366689000	6.162091000
1	1.333872000	-3.729161000	5.697128000
1	1.581485000	-2.134586000	4.965936000
6	5.184651000	-0.555964000	5.068118000
1	4.708417000	-0.558054000	6.049590000
1	6.236054000	-0.816569000	5.192231000
1	5.125947000	0.453223000	4.658299000
6	3.552166000	3.910308000	4.066443000
1	2.500959000	4.033168000	3.804271000
1	3.611375000	3.612359000	5.114150000
1	4.055581000	4.870723000	3.950402000
6	5.540891000	-1.041778000	-4.378985000

1	6.626379000	-1.141047000	-4.407569000
1	5.119584000	-1.969619000	-3.990170000
1	5.177905000	-0.899530000	-5.397819000
6	2.518035000	2.709958000	-5.159330000
1	2.924463000	1.799153000	-4.719159000
1	2.012223000	2.444170000	-6.088201000
6	-2.120974000	-4.716752000	-3.966268000
1	-1.554803000	-4.752878000	-4.897716000
1	-2.824670000	-5.549773000	-3.953884000
1	-2.682583000	-3.782380000	-3.945975000
6	-3.902460000	-0.238381000	-4.799816000
1	-3.357973000	0.664342000	-4.519196000
1	-3.360643000	-0.724945000	-5.611702000
1	-4.888926000	0.048373000	-5.166198000
1	3.342826000	3.384695000	-5.392584000
6	2.636460000	-4.838433000	-3.609018000
1	1.572510000	-4.631346000	-3.496001000
1	2.921902000	-4.609445000	-4.636638000
1	2.807517000	-5.900613000	-3.430098000

P6Q2 with two $AgOCOCF_3$

Sum of electronic and zero-point Energies= -4497.148406

Sum of electronic and thermal Energies= -4497.060727

Sum of electronic and thermal Enthalpies= -4497.059783

Sum of electronic and thermal Free Energies= -4497.283987

6	4.514259000	3.032708000	-0.152991000
6	4.581594000	1.536835000	-0.314780000
6	4.355520000	0.962403000	-1.562961000
6	4.475388000	-0.407552000	-1.755976000
6	4.880055000	-1.216449000	-0.693818000
6	5.070742000	-0.650325000	0.556988000
6	4.899047000	0.711287000	0.765736000
6	3.122701000	3.563937000	0.087718000
6	2.594128000	3.581216000	1.375964000
6	1.384372000	4.206061000	1.648415000
6	0.721911000	4.891721000	0.629318000
6	1.201574000	4.797859000	-0.667992000
6	2.374145000	4.110308000	-0.955841000
6	-0.459797000	5.774975000	0.954370000
6	-4.373227000	3.784745000	0.653829000
6	-3.802030000	4.319084000	-0.426556000
6	-2.480170000	4.965150000	-0.390963000
6	-1.776687000	5.060179000	0.925432000
6	-2.344974000	4.524859000	2.005893000
6	-3.656180000	3.853686000	1.965051000
6	-5.059047000	-2.668138000	-0.053409000
6	-3.664415000	-3.238740000	-0.127833000
6	-3.009448000	-3.645453000	1.025351000
6	-1.791037000	-4.311758000	0.970050000
6	-1.243740000	-4.632415000	-0.272214000
6	-1.856637000	-4.155280000	-1.425331000
6	-3.040095000	-3.432208000	-1.361614000
6	-0.046911000	-5.549686000	-0.359418000
6	5.079098000	-2.696903000	-0.905033000
6	3.772296000	-3.425387000	-0.825575000
6	3.067774000	-3.784472000	-1.899113000
6	1.773484000	-4.477995000	-1.802711000
6	1.250539000	-4.806689000	-0.438904000
6	1.940717000	-4.420314000	0.636771000
6	3.236528000	-3.726840000	0.538321000
8	4.233312000	-1.026576000	-2.937869000
6	3.684394000	-0.265197000	-4.000619000
8	5.039023000	1.312246000	1.973015000
6	5.074076000	0.497044000	3.132804000
8	2.882808000	3.978711000	-2.207737000
6	2.014437000	4.184467000	-3.310305000
8	0.813517000	4.235029000	2.878435000
6	1.360030000	3.405070000	3.890949000
8	-1.982207000	5.418431000	-1.400794000

8	-3.669605000	-2.928720000	-2.453320000	1	0.645571000	5.294502000	-1.451135000
6	-2.982632000	-2.949229000	-3.693313000	1	-0.490844000	6.589190000	0.230877000
8	-1.109549000	-4.737526000	2.064360000	1	-0.317107000	6.201904000	1.946234000
6	-1.464297000	-4.202098000	3.327167000	1	-4.285079000	4.283326000	-1.394699000
8	1.153604000	-4.778160000	-2.800678000	1	-1.866740000	4.569548000	2.976716000
8	3.861500000	-3.435808000	1.535805000	1	-5.587370000	-2.928502000	-0.969975000
8	-4.145264000	3.393306000	2.975338000	1	-5.578830000	-3.151499000	0.773426000
6	-5.690061000	3.068716000	0.622142000	1	-3.492730000	-3.453568000	1.973114000
6	-5.137010000	-1.174860000	0.131448000	1	-1.395903000	-4.387784000	-2.375153000
6	-5.191220000	-0.335393000	-0.970791000	1	-0.148265000	-6.172223000	-1.247506000
6	-5.332980000	1.037552000	-0.824499000	1	-0.030086000	-6.194309000	0.517364000
6	-5.478776000	1.582793000	0.448971000	1	5.528189000	-2.873648000	-1.881419000
6	-5.395010000	0.748092000	1.554059000	1	5.750148000	-3.079859000	-0.136846000
6	-5.196870000	-0.617141000	1.407588000	1	3.417706000	-3.574370000	-2.901741000
8	-5.360045000	1.909152000	-1.869412000	1	1.583799000	-4.620956000	1.639575000
6	-4.996525000	1.431706000	-3.150441000	1	2.843013000	0.327657000	-3.630638000
8	-5.075844000	-1.478254000	2.455757000	1	4.441296000	0.427883000	-4.386208000
6	-4.987024000	-0.947415000	3.762455000	1	6.021537000	-0.051614000	3.173489000
1	5.144108000	3.327199000	0.684937000	1	4.257743000	-0.229518000	3.091444000
1	4.917159000	3.497677000	-1.051165000	1	1.084880000	3.630570000	-3.147137000
1	4.096477000	1.622696000	-2.379047000	1	1.772060000	5.248597000	-3.403611000
1	5.341704000	-1.304342000	1.372847000	1	1.515963000	2.396931000	3.495079000
1	3.176421000	3.124479000	2.164311000	1	2.332165000	3.801928000	4.206915000

1	-1.979364000	-2.534019000	-3.561583000	8	0.869805000	0.890769000	-2.087964000
1	-2.884294000	-3.981997000	-4.046823000	6	-0.299730000	0.301308000	-4.062198000
1	-1.511255000	-3.110867000	3.255158000	9	-0.336768000	-0.804030000	-4.804018000
1	-2.450748000	-4.575051000	3.626467000	9	-1.536034000	0.817509000	-4.031675000
1	-6.288937000	3.455486000	-0.200918000	9	0.488734000	1.177325000	-4.682320000
1	-6.219950000	3.249463000	1.556646000	6	-0.409363000	-4.624989000	4.319302000
1	-5.120735000	-0.787677000	-1.950128000	1	0.565251000	-4.234410000	4.027532000
1	-5.476703000	1.198087000	2.533082000	1	-0.650958000	-4.232016000	5.307501000
1	-4.051327000	0.884021000	-3.081374000	1	-0.350703000	-5.712288000	4.382481000
1	-5.761182000	0.740334000	-3.523499000	6	4.923812000	1.399994000	4.332205000
1	-5.933548000	-0.468361000	4.038188000	1	5.723297000	2.141014000	4.363670000
1	-4.201244000	-0.185028000	3.802536000	1	3.964289000	1.916806000	4.297907000
9	1.845900000	0.049589000	4.697033000	1	4.959329000	0.808836000	5.247773000
9	2.114597000	-2.054883000	4.330501000	6	0.390669000	3.382221000	5.047560000
9	0.133375000	-1.252401000	4.655703000	1	0.789330000	2.759652000	5.849211000
8	-0.251195000	-1.067623000	-2.149431000	1	0.225852000	4.387147000	5.438208000
8	0.507304000	-1.699140000	1.996796000	1	-0.569066000	2.967092000	4.737051000
8	1.712248000	0.207034000	2.086235000	6	-4.671685000	-2.087505000	4.701688000
6	0.170900000	0.009861000	-2.607548000	1	-3.711399000	-2.538516000	4.447436000
6	1.164707000	-0.800553000	2.554289000	1	-4.617706000	-1.721728000	5.727761000
6	1.318133000	-1.011517000	4.088292000	1	-5.442857000	-2.856743000	4.647856000
47	0.022113000	-1.684713000	-0.104186000	6	-4.854069000	2.619939000	-4.070938000
47	1.747255000	0.904021000	-0.034385000	1	-5.786540000	3.183216000	-4.123030000

1	-4.061598000	3.284335000	-3.724172000
1	-4.598750000	2.280268000	-5.075545000
6	-3.779351000	-2.125095000	-4.675555000
1	-3.851590000	-1.091356000	-4.336264000
1	-3.286221000	-2.130599000	-5.648195000
6	2.723666000	3.690813000	-4.547791000
1	2.091209000	3.841248000	-5.423225000
1	3.660587000	4.228387000	-4.698237000
1	2.938996000	2.625197000	-4.462562000
6	3.230141000	-1.223944000	-5.074118000
1	2.459942000	-1.895086000	-4.691758000
1	2.810200000	-0.666722000	-5.912185000
1	4.065325000	-1.822445000	-5.439774000
1	-4.786763000	-2.526301000	-4.793543000

P6Q3 with two $AgOCOCF_3$

Sum of electronic and zero-point Energies= -4338.792638

Sum of electronic and thermal Energies= -4338.710817

Sum of electronic and thermal Enthalpies= -4338.709873

Sum of electronic and thermal Free Energies= -4338.924367

6	-1.273297000	5.641344000	0.145025000
6	-2.239789000	4.528089000	-0.178102000
6	-3.016046000	3.952144000	0.818714000
6	-3.974490000	2.986965000	0.519863000
6	-4.167270000	2.604781000	-0.812172000
6	-3.364528000	3.155497000	-1.801322000
6	-2.391023000	4.096044000	-1.495831000
6	0.153060000	5.192386000	0.277746000
6	1.037281000	5.255123000	-0.717348000
6	2.447763000	4.861443000	-0.552837000
6	2.922409000	4.497645000	0.818835000
6	2.026311000	4.381682000	1.800274000
6	0.591926000	4.662719000	1.604976000
6	4.394089000	4.266642000	1.004456000
6	5.633427000	0.157909000	0.715340000
6	5.362784000	0.727219000	1.950524000
6	4.909102000	2.034878000	2.054247000
6	4.776376000	2.809606000	0.903468000
6	5.011348000	2.230070000	-0.334608000
6	5.411185000	0.904855000	-0.440680000
6	2.118611000	-5.457673000	-0.136879000
6	0.725958000	-4.960802000	-0.439693000
6	0.363393000	-4.629946000	-1.735889000
6	-0.928492000	-4.224295000	-2.044141000
6	-1.901624000	-4.216420000	-1.044303000
6	-1.528108000	-4.498235000	0.269646000

6	-0.216298000	-4.844887000	0.586551000	8	5.634289000	0.274260000	-1.623156000
6	-3.349947000	-3.970036000	-1.396440000	6	5.249049000	0.923569000	-2.822089000
6	-5.247629000	1.610085000	-1.165676000	6	6.182766000	-1.245697000	0.618963000
6	-4.729718000	0.202055000	-1.151717000	6	3.092752000	-4.342725000	0.108646000
6	-4.749135000	-0.578084000	-0.068003000	6	3.353168000	-3.846099000	1.318423000
6	-4.243394000	-1.962916000	-0.080109000	6	4.359490000	-2.790424000	1.545805000
6	-3.743862000	-2.522352000	-1.374563000	6	5.118931000	-2.272804000	0.365785000
6	-3.673231000	-1.725592000	-2.441637000	6	4.827101000	-2.741377000	-0.848117000
6	-4.130017000	-0.324850000	-2.418615000	6	3.802671000	-3.772987000	-1.079262000
8	-4.779591000	2.404971000	1.440350000	8	4.583921000	-2.383316000	2.665798000
6	-4.538735000	2.657793000	2.817476000	8	3.560256000	-4.166529000	-2.201208000
8	-1.567984000	4.649790000	-2.417022000	1	-1.325276000	6.392635000	-0.642752000
6	-1.479747000	4.037079000	-3.695552000	1	-1.578733000	6.106659000	1.081952000
8	-0.203832000	4.499874000	2.506668000	1	-2.861658000	4.280280000	1.836518000
8	3.204061000	4.857299000	-1.501056000	1	-3.513926000	2.821779000	-2.817973000
8	4.594607000	2.635976000	3.234053000	1	0.755923000	5.605321000	-1.702111000
6	4.662519000	1.873408000	4.423859000	1	2.320108000	4.085417000	2.800488000
8	0.212658000	-5.110944000	1.842002000	1	4.686964000	4.647135000	1.981336000
6	-0.704370000	-4.981404000	2.922213000	1	4.932039000	4.825124000	0.239398000
8	-1.323359000	-3.847219000	-3.283376000	1	5.507329000	0.116439000	2.829976000
6	-0.319149000	-3.517921000	-4.235091000	1	4.879876000	2.845012000	-1.214440000
8	-4.244969000	-2.634535000	0.929740000	1	2.087062000	-6.097654000	0.742779000
8	-4.044824000	0.369795000	-3.408250000	1	2.470620000	-6.046542000	-0.983417000

1	1.122190000	-4.691382000	-2.502989000	1	5.343966000	-2.384036000	-1.729846000
1	-2.293716000	-4.463597000	1.032088000	9	-0.149148000	0.657851000	-4.371559000
1	-3.976036000	-4.509685000	-0.686793000	9	1.616396000	-0.410369000	-3.745695000
1	-3.539933000	-4.365250000	-2.393193000	9	1.396242000	1.672445000	-3.252401000
1	-6.065120000	1.694499000	-0.452181000	8	-1.353678000	-1.692666000	2.284580000
1	-5.625588000	1.836483000	-2.161741000	8	0.121315000	-1.023550000	-1.683353000
1	-5.139160000	-0.223968000	0.878581000	8	-0.642892000	1.096179000	-1.569031000
1	-3.296187000	-2.086065000	-3.390588000	6	-2.078716000	-0.797695000	2.744778000
1	-3.495531000	2.430530000	3.052327000	6	-0.001442000	0.151883000	-2.061971000
1	-4.727881000	3.715078000	3.033760000	6	0.739116000	0.518926000	-3.378906000
1	-2.406357000	4.211834000	-4.253511000	47	-0.690569000	-1.782399000	0.198933000
1	-1.351720000	2.958712000	-3.569598000	47	-1.771026000	0.975685000	0.285238000
1	4.045442000	0.973778000	4.324242000	8	-2.467849000	0.262498000	2.223478000
1	5.695710000	1.554612000	4.602916000	6	-2.613577000	-1.037929000	4.184254000
1	-1.156831000	-3.987015000	2.902765000	9	-3.882082000	-1.455158000	4.127601000
1	-1.496947000	-5.730694000	2.816912000	9	-1.908734000	-1.960786000	4.833174000
1	0.428936000	-2.876908000	-3.760708000	9	-2.580618000	0.083588000	4.904237000
1	0.176293000	-4.431657000	-4.580086000	6	-0.987913000	-2.803807000	-5.384218000
1	4.214136000	1.269972000	-2.738524000	1	-1.445897000	-1.872819000	-5.048352000
1	5.885513000	1.799113000	-2.992253000	1	-0.247229000	-2.560221000	-6.146657000
1	6.688371000	-1.489443000	1.553133000	1	-1.756769000	-3.430782000	-5.837617000
1	6.911491000	-1.292492000	-0.189598000	6	-0.298788000	4.637651000	-4.418303000
1	2.851755000	-4.219260000	2.203779000	1	-0.413968000	5.717631000	-4.520738000

1	0.628285000	4.430807000	-3.883275000
1	-0.220115000	4.203550000	-5.415750000
6	5.388243000	-0.069414000	-3.950912000
1	5.117247000	0.404453000	-4.895114000
1	6.415787000	-0.427394000	-4.026813000
1	4.728816000	-0.924087000	-3.796321000
6	0.065658000	-5.186638000	4.203242000
1	0.838236000	-4.424608000	4.311488000
1	-0.611742000	-5.106886000	5.053980000
6	-5.463416000	1.769178000	3.610808000
1	-5.257216000	0.720914000	3.396595000
1	-5.308551000	1.933842000	4.677473000
1	-6.506638000	1.981822000	3.373676000
6	4.166475000	2.740297000	5.556349000
1	3.131318000	3.039932000	5.388144000
1	4.215402000	2.187657000	6.495366000
1	4.777714000	3.638378000	5.651238000
1	0.536336000	-6.170327000	4.224382000

----

7. References:

[1] Boinski, T.; Szumna, A., A facile, moisture-insensitive method for synthesis of pillar[5]arenes—the solvent templation by halogen bonds. *Tetrahedron* **2012,** *68*, 9419-9422.

[2] Cao, J.; Shang, Y.; Qi, B.; Sun, X.; Zhang, L.; Liu, H.; Zhang, H.; Zhou, X., Synthesis of pillar[n]arenes (n = 5 and 6) with deep eutectic solvent choline chloride 2FeCl3. *RSC Adv.* **2015,** *5*, 9993-9996.

[3] Rashvand Avei, M.; Etezadi, S.; Captain, B.; Kaifer, A. E., Visualization and quantitation of electronic communication pathways in a series of redox-active pillar[6]arene-based macrocycles. *Chem. Comm.* **2020,** *3*, 117.

[4] Hua, B.; Shao, L.; Zhang, Z.; Liu, J.; Huang, F., Cooperative Silver Ion-Pair Recognition by Peralkylated Pillar[5]arenes. *J. Am. Chem. Soc.* **2019,** *141*, 15008-15012.

[5] Thordarson, P., Determining association constants from titration experiments in supramolecular chemistry. *Chem. Soc. Rev.* **2011,** *40*, 1305-1323.

[6] Brynn Hibbert, D.; Thordarson, P., The death of the Job plot, transparency, open science and online tools, uncertainty estimation methods and other developments in supramolecular chemistry data analysis. *Chem. Comm.* **2016,** *52*, 12792-12805.

[7] Ulatowski, F.; Dąbrowa, K.; Bałakier, T.; Jurczak, J., Recognizing the Limited Applicability of Job Plots in Studying Host–Guest Interactions in Supramolecular Chemistry. *J. Org. Chem.* **2016,** *81*, 1746-1756.

[8] Chao, Y.; Thikekar, T. U.; Fang, W.; Chang, R.; Xu, J.; Ouyang, N.; Xu, J.; Gao, Y.; Guo, M.; Zuilhof, H.; Sue, A. C.-H., "Rim-Differentiated" Pillar[6]arenes. *Chem. Int. Ed.* **2022,** *61*, e202204589.

[9] Hristova, Y. R.; Smulders, M. M. J.; Clegg, J. K.; Breiner, B.; Nitschke, J. R., Selective anion binding by a "Chameleon" capsule with a dynamically reconfigurable exterior. *Chem. Sci.* **2011,** *2*, 638-641.

[10] Gaussian 16, Revision B.01, M. J. Frisch, G. W. Trucks, H. B. Schlegel, et al., Gaussian, Inc., Wallingford CT, 2016.

[11] F. Weigend, R. Ahlrichs, PCCP, 7(2005) 3297-3305.
